# Locomotion in Extinct Giant Kangaroos: Were Sthenurines Hop-Less Monsters?

**DOI:** 10.1371/journal.pone.0109888

**Published:** 2014-10-15

**Authors:** Christine M. Janis, Karalyn Buttrill, Borja Figueirido

**Affiliations:** 1 Department of Ecology and Evolutionary Biology, Brown University, Providence, Rhode Island, United States of America; 2 Departamento de Ecología y Geología, Facultad de Ciencias, Universidad de Málaga, Málaga, Spain; New York Institute of Technology College of Osteopathic Medicine, United States of America

## Abstract

Sthenurine kangaroos (Marsupialia, Diprotodontia, Macropodoidea) were an extinct subfamily within the family Macropodidae (kangaroos and rat-kangaroos). These “short-faced browsers” first appeared in the middle Miocene, and radiated in the Plio-Pleistocene into a diversity of mostly large-bodied forms, more robust than extant forms in their build. The largest (*Procoptodon goliah*) had an estimated body mass of 240 kg, almost three times the size of the largest living kangaroos, and there is speculation whether a kangaroo of this size would be biomechanically capable of hopping locomotion. Previously described aspects of sthenurine anatomy (specialized forelimbs, rigid lumbar spine) would limit their ability to perform the characteristic kangaroo pentapedal walking (using the tail as a fifth limb), an essential gait at slower speeds as slow hopping is energetically unfeasible. Analysis of limb bone measurements of sthenurines in comparison with extant macropodoids shows a number of anatomical differences, especially in the large species. The scaling of long bone robusticity indicates that sthenurines are following the “normal” allometric trend for macropodoids, while the large extant kangaroos are relatively gracile. Other morphological differences are indicative of adaptations for a novel type of locomotor behavior in sthenurines: they lacked many specialized features for rapid hopping, and they also had anatomy indicative of supporting their body with an upright trunk (e.g., dorsally tipped ischiae), and of supporting their weight on one leg at a time (e.g., larger hips and knees, stabilized ankle joint). We propose that sthenurines adopted a bipedal striding gait (a gait occasionally observed in extant tree-kangaroos): in the smaller and earlier forms, this gait may have been employed as an alternative to pentapedal locomotion at slower speeds, while in the larger Pleistocene forms this gait may have enabled them to evolve to body sizes where hopping was no longer a feasible form of more rapid locomotion.

## Introduction

### Kangaroo diversity past and present

**Figure 1:**
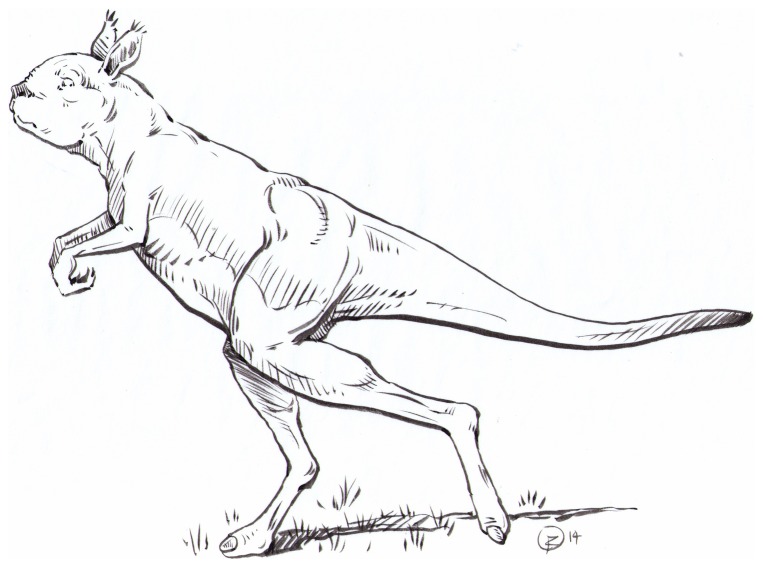
Reconstruction of *Sthenurus stirlingi*. By Brian Regal.

Kangaroos are famous for their style of locomotion – bipedal hopping (also known as ricochetal or saltatory locomotion), which is unique among relatively large mammals (i.e., over around 5 kg in body mass). While the popular notion of a kangaroo is of a fairly large animal, such as the grey kangaroo (*Macropus* [*Macropus*] *giganteus*) or the red kangaroo (*Macropus* [*Osphranter*] *rufus*), members of the superfamily Macropodoidea (“kangaroos” in the broadest sense) contain animals of a diversity of sizes and habits, including the secondarily arboreal tree-kangaroos (*Dendrolagus* spp.). However, the recent past diversity of kangaroos, persisting to perhaps as recently as 30,000 years ago, included several kinds of kangaroos much larger than any known at present. The largest of the extant kangaroos (red kangaroo males) can weigh up to 90 kg, although the average weight for males of this species is only around 55 kg, with females averaging around 25 kg [1], [2]. However, Pleistocene kangaroos existed that weighed up to 240 kg [2], a size that calls into question their biomechanical abilities for a hopping gait [3]. Three different lineages of macropodids, two of them extinct, attained masses of greater than any extant kangaroo (i.e., >90 kg); kangaroos larger than extant kangaroos are commonly referred to as “giant kangaroos” [2]. In this paper we specifically address the locomotor abilities of the extinct subfamily Sthenurinae, and propose that they employed a bipedal striding type of gait (see Figure 1). We propose that this gait would have been used at only at slow speeds in the smaller sthenurines, with hopping employed at faster speeds, but in the very large sthenurine species this may have been their sole mode of locomotion.

There are three families within the Macropodoidea (taxonomy following Prideaux and Warburton [4], see Table S1): the Balbaridae (an extinct family, considered to be basal to both extant families [5]); the Hypsiprymnodontidae (the extant musky rat-kangaroo, *Hypsiprymnodon moschatus*, plus a number of extinct genera); and the Macropodidae. The family Macropodidae is usually divided into four subfamilies: Potoroinae (rat-kangaroos), Lagostrophinae (containing the extant banded hare-wallaby, *Lagostrophus fasciatus*, and the extinct genus *Troposodon*), Macropodinae (containing all other extant macropodids, olus several extinct genera including *Protemnodon*), and Sthenurinae (containing the extinct genera *Archaeosimus*, *Hadronomas, Metasthenurus, Procoptodon*, *Rhizosthenurus*, *Sthenurus, Simosthenurus*, and *Wanburoo*, see Prideaux [6]). A possible fifth subfamily is the Bulungamayinae, which is a paraphyletic assemblage of extinct taxa basal to the Macropodinae plus Sthenurinae [5]). Molecular phylogenies (e.g., [7]) show similar relationships among the living taxa, although the higher level taxonomic terminology differs (e.g., the rat-kangaroos are considered to be a separate family, Potoroidae). “Giant” forms can be found within the Sthenurinae (among the genera *Sthenurus, Simosthenurus*, and *Procoptodon*) [6], and within the Macropodinae in the extant genus *Macropus* (extinct species *M. titan* and *M. ferragus*), and the extinct genus *Protemnodon* (*P. brehus, P. roechus*, and *P. anak*).

The genus *Macropus* has species of a diversity of body sizes, the smallest today being *Macropus parma* (the parma wallaby) with an average body mass of around 4 kg [8]. *Macropus titan* (related to the extant grey kangaroo) had an estimated body mass of up to 150 kg [2]. A similar estimate has been obtained for *M. ferragus* (related to the extant red kangaroo) [9], although this animal appears to have been somewhat larger in linear dimensions than *M. titan*. Within the genus *Protemnodon*, all species were fairly large, although not all were larger than extant kangaroos. *Protemnodon hopei* had an estimated mass of 45 kg [2]. Other smaller *Protemnodon* species, such as *P. otibandus* and *P. snewini*, were of a similar size (as estimated by measurements of foot bones). The largest species was *P. roechus*, with an estimated mass of around 166 kg [2]. Protemnodon spp. are commonly referred to as “giant wallabies”: but while they might be somewhat wallaby-like in their skull and dentition, their postcranial elements are unlike any other terrestrial macropodid; they have relatively short tibiae, short feet and (at least in *P. anak*) a relatively long neck. There has been speculation that at least some may have been secondarily quadrupedal in their locomotion [10], [11].

**Figure 2:**
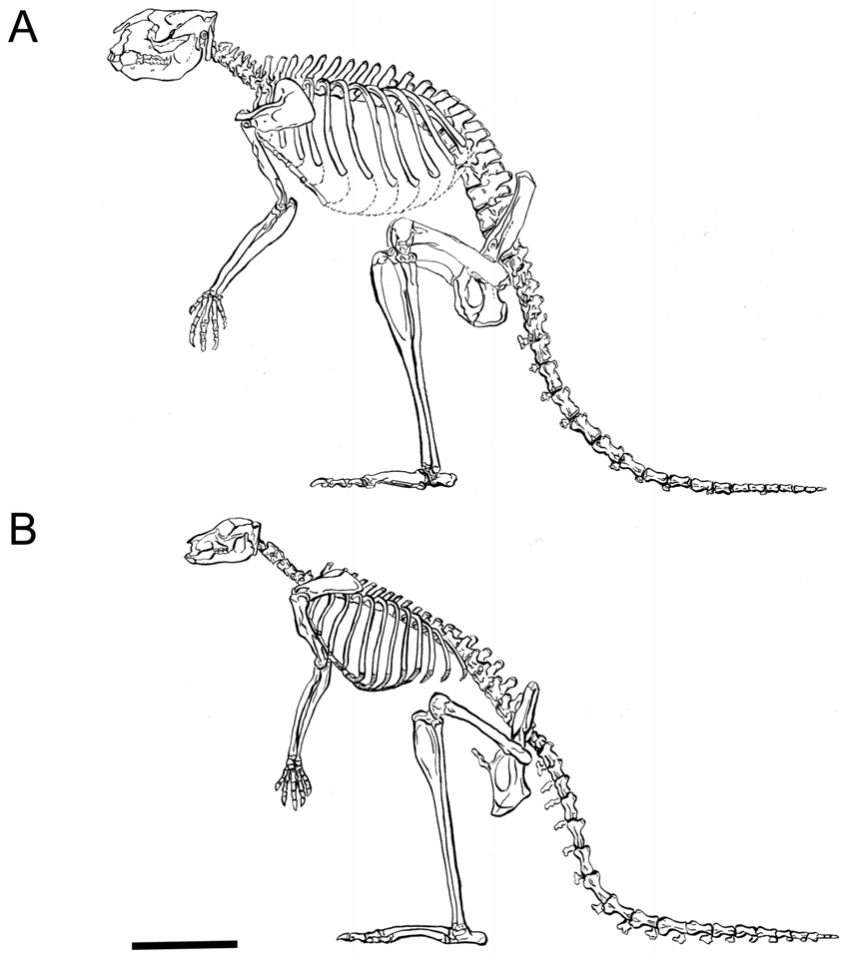
Skeletons of (A) *Sthenurus stirlingi* and (B) *Macropus giganteus*. Modified from Wells and Tedford, 1995. Original artist Lorraine Meeker, American Museum of Natural History (reproduced here by permission).

Sthenurines are the kangaroos that are usually spoken of as being the “giant kangaroos”. They are distinguished not only by their size, but by many other features, such as their relatively short faces, the loss of the fifth digit in the foot in the Pleistocene forms, and their specialized arms and hands that have been interpreted as an adaptation for browsing [12]. Sthenurines have also been noted as having especially robust limb bones, but the bones of the larger species of the extinct genus *Protemnodon* are similarly robust (see later discussion). Sthenurines, like Protemnodon spp., were all fairly large, but not all species were larger than extant kangaroos. Figure 2 shows the difference in the skeletons of Pleistocene sthenurines and large macropodines.

A few smaller sthenurines are known from the Miocene, and these forms retained the fifth toe. The middle Miocene *Wanburoo hilarus* ([13]), originally described as a bulungamayine, was the smallest known sthenurine [4]), with an estimated body mass of 7–8 kg [14]. Unfortunately, postcranial materials from this animal are unknown. The slightly larger (9–15 kg [14]) middle [15] and early late Miocene *Rhizosthenurus flanneryi* [16] is known from a partial skeleton as well as cranial material. The late late Miocene [17] *Hadronomas* had estimated body mass of around 30 kg [4]. Note that Prideaux [6] has determined that several species of *Sthenurus* are on the stem lineage of the genus *Procoptodon*, although lacking the specialties of this highly derived taxon, and has renamed them as “*Procoptodon*”. These include two smaller species (body mass of around 50 kg [2]) included here; *“P. gilli* and *“P”. browneorum*.

### Locomotion in macropodoids

Hopping is the quintessential kangaroo gait, seen in all extant macropodoids with the exception of the musky rat-kangaroo (*Hypsiprymnodon moschatus*), where it is considered to be primarily absent (although this is conjectural because there are inadequate data on this animal locomoting at high speeds [18]. Some earlier, extinct macropodoids (Balbaridae) may have been quadrupedal bounders rather than hoppers [11]. All extant kangaroos have a digitigrade hind foot posture while locomoting, although they may rest with the hind feet in a plantigrade stance. Among the potoroines (rat-kangaroos), potoroos (*Potorous* spp.) habitually bound and only hop at high speed when alarmed [19], while bettongs (*Bettongia* spp.) habitually hop like macropodines [20]. Within the macropodines, all species use the form of slow, pentapedal progression while foraging on the ground [21], a gait that is actually used more frequently than hopping during the course of the day [22]. In the pentapedal “walk”, the forefeet are placed together on the ground, the hind feet are lifted, and the tail is used as a “fifth leg” to propel the animal through the space between the fore feet [22]. A quadrupedal bound is seen in the quokka (*Setonix brachyurus*) and tree-kangaroos (*Dendrolagus* spp.), and tree-kangaroos uniquely perform (along tree branches) a quadrupedal walk, with alternate movement of the limbs [21]. Tree-kangaroos have also been observed walking bipedally along branches [23].

The adaptive reasons for adopting the hopping gait are not entirely clear. Hopping is clearly a very efficient form of locomotion in larger kangaroos (see discussion below), but it would have first evolved in relatively small forms, likely of a mass of less than 5 kg [24]. Baudinette [25], Bennett [26], and McGowan et al. [3] summarize much recent information about kangaroo hopping, and the discussion below is derived from their papers. Hopping has evolved several times in rodents, but most of these are very small, with the largest being the springhare (*Pedetes capensis*) with a body mass of around 4 kg (the size of a small wallaby). These rodents, like small macropodids, use quadrupedal gaits at slow speeds [18]. However, the ankle extensor tendons of hopping rodents are more robust for their size than in kangaroos, which may reflect the need to withstand relatively high forces during acceleration. Hopping can also be seen in some lemuriform primates (e.g., sifakas moving on the ground) and occasionally in Arctic hares (Lagomorpha): again, these animals are no more than around 5 kg in weight, below the weight where hopping becomes efficient in terms of storage of elastic energy in the extensor tendons (see below).

It is well known that, in larger kangaroos (e.g., *Macropus rufus*), hopping is an extremely efficient gait: unlike the situation in placentals, where the energy costs of locomotion increase linearly with speed, in the larger kangaroos energy costs and speed become decoupled, and thus the daily expenditure of locomotor foraging is much less for the large kangaroos than for similarly-sized cursorial placentals. (Note: although the term “cursorial” usually refers to quadrupedal locomotion, we use the term here in relation to kangaroo locomotion, where “more cursorial” equals “more specialized for fast hopping”.) Even in a medium-sized wallaby, the energy savings from elastic energy storage during hopping have been estimated at around 40% [27]. However, while much of the research focus has been on the spectacular performance of the large kangaroos, this cannot explain the initial reason for adopting a hopping gait. This decoupling of energy costs and speeds is only true in kangaroos above around 6 kg, and the smaller potoroines (e.g., *Potorous*, *Bettongia*) show no such advantage, although hopping in *Bettongia* (at least at relatively high speeds, above 3 m/sec) is nevertheless less expensive than locomotion in a quadruped of similar size [28]. However, one distinct advantage of hopping, over and above any storage of elastic energy, is that speed can be attained simply by increasing stride length without concomitant increase in stride frequency, which reduces the energetic costs of limb recycling [28].

The pentapedal walk, which is employed at low speeds in kangaroos, is energetically very expensive, more so than hopping at higher speeds [28]. Kangaroos have been shown to progress from a pentapedal gait to hopping at a Froude number of 0.5 [25], which is similar to the transition to the gallop in quadrupeds (the Froude number relates size, speed, and stride length, and is used in the analysis of vertebrate gaits). There must thus be some biomechanical or energetic reason why hopping cannot be employed at low speeds: Dawson [29] proposed (p. 68) that, due to the specialized limb morphology of kangaroos, hopping would likely be even more expensive than pentapedal locomotion at slow speeds. The role of the tail is also important in hopping in kangaroos: in bipedal or quadrupedal running there is no net torsion on the body that would cause the head to pitch, as the angular momentum of the legs cancel each other out. But in bipedal hopping, where the legs act together in phase, their action creates a moment around the center of mass, such that the body would tend to pitch with each hop. Motion of the tail reduces this tendency: the tail swings forwards as the hind legs swing backwards, thus cancelling out the moment produced by the limbs, and reducing the effective pitch of the head to around ten degrees [27].

However, larger kangaroos pay a price for their locomotor efficiency in terms of bone and tendon stress. Placental mammalian cursors change their posture with increasing body size, thereby reducing the torque of the ground reaction force around their limbs [30], but large kangaroos hop with the same flexed limb posture as smaller ones. There is no evidence that there is any difference in the properties of the ankle extensor muscles and their tendons that power hopping in kangaroos (gastrocnemius, flexor digitorum longus [ =  profundus], and plantaris [ =  flexor digitorum superficialis]) in comparison with similar placentals, although enzyme levels in these muscles in kangaroos may indicate higher levels of aerobic work, and 86% of the total body mitochondrial volume in *M. rufus* is in the upper hind limb musculature [28]. In addition, bone stresses on the tibia appear to be greater in large kangaroos (but not in smaller kangaroos) than in similar-sized placentals, although there is some evidence that tibial bone cortical thickness increases with increasing body size in kangaroos [3].

Certain allometric scaling relationships differ between kangaroos and placental mammals, which likely relates to the fact that large kangaroos maintain the crouched posture of smaller ones, rather than altering their posture with increasing body size, see [30]. The overall hind limb length of macropodoids scales with positive allometry, largely due to strong positive allometry of tibia length [3]. The size of the hind limb, the limb muscles, and the cross sectional area of the foot extensors all scale with isometry in placentals. All of the extensor muscles of the hind limb in macropodines, with the exception of the sartorius, scale with strong positive allometry, while fascicle length tends to scale with negative allometry, resulting in extremely strong positive allometry for hind limb muscle physiological cross sectional area [3]. However, note that in potoroines, elastic energy saving is primarily in the plantaris muscle, with the gastrocnemius and flexor digitorum longus being more involved in joint control and acceleration capacity [3]. This greater muscle power in kangaroos is required to offset the relatively greater torques around the ankle joint in the absence of changing posture with increasing size. In contrast, the tendon cross sectional areas of the gastrocnemius and flexor digitorum longus scale with negative allometry, although the cross sectional areas of the plantaris tendon scales with positive allometry [3]. While this anatomy allows for greater proportional muscle power and elastic energy storage in larger kangaroos, accounting in part for their superior locomotor performance, it appears that larger macropodids operate with unusually high musculoskeletal stresses, and tendon safety factor (estimated from the ratio of the tendon cross sectional area to the physiological cross sectional area of the attached muscle (see [3]: at safety factors below one tendon failure becomes likely [31]) might be a limiting factor for body mass and/or locomotor performance.

The mechanical tensile stress of tendons plays a limiting factor in body size and speed in all mammals. In placental mammals maximum locomotor performance peaks at a body mass of around 50 kg (cheetah or pronghorn size), and there is evidence that this is the size at which strain on the locomotor tendons becomes an issue. How does this relate to the condition in kangaroos? While larger species will be better able to use elastic energy recovery to assist in their locomotion, the larger the animal, the lower the tendon safety factor, and the greater the danger of tendon rupture: while smaller kangaroos have estimated tendon safety factors of around ten (typical for non-hopping mammals), the estimated safety factor of the Achilles tendon in large kangaroos approaches one. McGowan et al. [3] estimate that, at a projected body mass of around 140 kg, a fast-hopping kangaroo would have a tendon safety factor of less than one. However, a large male red kangaroo (*Macropus rufus*), weighing around 80–90 kg, would still be operating with a very low tendon safety factor of 1.1, and many extinct kangaroos have body mass estimates well in excess of 140 kg (although this is around the mass estimate for the largest extinct species of *Macropus*). Few extant kangaroo individuals can be found with a body mass of greater than around 50 kg, and most kangaroos weigh considerably less than this, see [1]. Extrapolations from the data of McGowan et al. [3] would predict that the sthenurine *Procoptodon goliah*, at a body mass of as much as 250 kg, would have had a tendon safety factor of around 0.89; they note that hopping would have been severely limited in this animal, especially during acceleration, if indeed possible at all. However, McGowan et al. [3] propose that relatively thicker tendons in these larger macropodids (as also seen in rock wallabies, *Petrogale* spp.) would have enabled them to exert higher forces at larger sizes. McGowan [32] presented evidence that the greatly enlarged site of attachment of the gastrocnemius (i.e., the Achilles tendon) on the calcaneum in sthenurines was indicative of tendons sufficiently large to withstand the forces of hopping locomotion. However, it is also true that a relatively thicker tendon has a lesser capacity for elastic energy storage and, as noted below, the moment arm for the gastrocnemius in sthenurines is much shorter than in large macropodines. Obviously large sthenurines would have had to have relatively large gastrocnemius muscles to support and propel their body mass during locomotion, but this does not necessarily mean that they could hop well, if at all.

### Descriptive anatomy of sthenurine kangaroos

While sthenurine locomotor performance has rarely been considered as markedly different from that of modern large kangaroos (genus *Macropus*), there are many anatomical differences that reflect the fact that these animals had a rather different functional biology. The classic work on sthenurine anatomy is that of Wells and Tedford [12], in their comparison of species of *Sthenurus* (mainly *S. stirlingi* and *S. tindalai*) with the exant grey kangaroo, *Macropus giganteus*): they note that, while the overall postcranial proportions of *Sthenurus* resembles those of *Macropus*, there are some key differences.

- While the forelimbs of *Macropus* and *Sthenurus* are of similar overall length, the manus of sthenurines is longer, and the radius and ulna are shorter. Modifications of the sthenurine forelimb allow for specialized grasping, and the scapular morphology may allow for elevation of the forelimb over the head, as in humans. Somewhat similar modifications of the forelimb, especially in the scapula, are seen in tree- kangaroos, which do extensive reaching with the forelimbs [33].
- The hind limbs are of similar proportions in both, but Wells and Tedford [12] claim that the limbs are proportionally longer in relation to the vertebral column in *Sthenurus*. However, this is not apparent in their figures (see Figure 2): *Sthenurus* appears to have a somewhat *shorter* trunk than *Macropus*, which would mean that this apparent difference in proportions is due to a shorter trunk rather than to longer hind limbs. (See [34], which concludes that sthenurines have a relatively shorter vertebral column than macropodines, despite the same number of vertebrae, due to a shorter length of the lumbar vertebrae.)
- Pleistocene sthenurines have reduced the fifth digit in the hind limb to a vestigial nubbin of the metatarsal. There is little evidence that they retained the syndactylous small second and third digits in the hind limb typical of other kangaroos (although a remnant of metatarsal III is known in a couple of specimens [12]).
- The tail of *Sthenurus* is slightly shorter than that of *Macropus*. In addition, the anterior caudal vertebrae have shorter (although robust) centra, but with shorter diapophyses and metapophyses, and vestigial anapophyses. This may indicate a reduced capacity for lateral extension of the tail [12].
- The lumbar vertebrae of *Sthenurus* are massive, with huge metapophyses, but the transverse processes have been reduced or lost, and the backbone appears to have been relatively rigid with limited flexion in the dorsoventral plane.
- And, finally, in general the limb elements of *Sthenurus*, especially in the hind limbs, are much more robust than those of *Macropus*: Wells and Tedford [12] note that the cross-sectional area of the femur of *Sthenurus* is almost double that of a *Macropus* of similar linear dimensions.

We present here a description of the hindimb locomotor anatomy of sthenurine kangaroos, which is essential for an understanding of the analyses performed. Much of this anatomical description is drawn from Wells and Tedford [12], but some novel features are also included. Note that the anatomical illustrations here are designed to show features not emphasized in Wells and Tedford [12], and the reader is referred to this publication for additional details. The supplementary information contains a number of bivariate plots (Figures S1–S3), which are separate from the multivariate analyses discussed later, and provide a visual impression of some of the anatomical differences between sthenurines and other kangaroos.

#### Vertebral column

The number of precaudal vertebrae is similar in sthenurines and macropodines (7 cervical, 13 thoracic and 6 lumber), but the overall appearance of the vertebral column is very different. The overall appearance in sthenurines is for massive vertebrae in comparison to macropodines, especially in the lumbar region, where large and laterally expanded metapophyses give the impression of rigidity. Wells and Tedford [12] note that the anticlinal vertebra in *Sthenurus* is more posterior (L1 or L2, versus T10 or T11 in *Macropus*), with no modification of the nature of the zygapophyses (as in *Macropus*). Large metapophyses appear on T11, and increase in size dramatically in the more posterior portion of the trunk. While metapophyses are also present in the posterior trunk of *Macropus*, they are smaller, and less deflected medially. The neural arches are shorter in *Sthenurus*, although broader in the anteroposterior direction, and are directed caudally (while those of *Macropus* are directed cranially). The lumbar vertebrae of *Sthenurus* are also markedly different in having greatly reduced transverse processes, and in the vestigial nature or absence of diapophyses and anapophyses (that are prominent on the lateral surfaces of the lumbar vertebrae in macropodines), and the almost platycoelus centra indicate limited mobility.

Wells and Tedford [12] interpret this anatomy as indicating rigidity to resist rotational stress on the backbone. In *Sthenurus* resistance would have been effected not only by the zygapophyses, but also by by the epaxial muscles (multifidi) and ligaments attached to the greatly enlarged metapophyses. They [12] note that the loss of diapophyses and anapophyses indicate the reduction of the longissimus dorsi component of the epaxial musculature. They [12] interpret the massive dorsal epaxial components (the multifidi) as being used to elevate the front half of the body for the proposed browsing posture. But this interpretation is problematical. The notion of the “elevation of the body” seems to be derived from the function proposed by Elftman [35] in kangaroos for the erector spinae muscles (this being the lumbar region merging of multifidus and longissimus dorsi muscles); but this proposed function was in the context of preventing the front end of the body collapsing under the force of gravity during locomotion. It would be impossible for the erector spinae to raise the body up over the hip, as they insert cranially to the hip joint (along the dorsomedial border of the ilium). The muscles that would be capable of exerting this action of raising the body would be ones that span the hip joint dorsally: the gluteus superficialis and the cranial head of the caudofemoralis ( =  gluteobiceps) muscles (see [36]), that originate from the tuber coxae of the ilium (and/or the thoracolumbar fascia, see below) and run dorsally over the hip socket to insert on the femur.

#### Sacrum

The sacrum of sthenurines is broader and shorter than in macropodines, with more pronounced sacral wings (alae sacrales) for the articulation with the ilium. Wells and Tedford [12] note only two sacral vertebrae in *Sthenurus*, as in *Macropus* (and most other marsupials), but the senior author has observed sthenurine sacra that are composed of three vertebrae, involving the incorporation of the first caudal vertebra (e.g., *Simosthenurus occidentalis* SAM P18308). Wells and Tedford [12] note a more rigid sacrolumbar connection in *Sthenurus stirlingi* than in *Macropus* (whereas the smaller *S. tindalei* is more like *Macropus*). The sthenurine sacral anatomy is again indicative of a resistance to rotational torsion.

All macropoidoids exhibit the derived anatomy of a very distally-positioned attachment of the ilium to the sacrum, with the result that the ilia project dorsally above the sacrum. Personal observation by the senior author shows that this is also true for the musky rat-kangaroo (*Hypsiprymnodon moschatus*, the only extant macropodoid considered to be primarily non-hopping), but to a slightly lesser extent than in other macropodoids, and it is also a feature of koalas and wombats. Elftman [35] interprets this morphology as allowing for a greater area for the insertion of the erector spinae muscles, which insert along the dorsomedial border of the ilium anterior to the sacral attachment, and notes that it also allows for an increased sacroiliac angle.

#### Pelvis (Figure 3)

The ilium of both *Sthenurus* and *Macropus* is long, but in *Sthenurus* the ilia flare more laterally, and have a greatly expanded blade. Elftman [35] interprets flared ilia as allowing for a greater volume of erector spinae musculature. Wells and Tedford [12] note that the areas of insertion for the gluteal muscles (on the lateral side of the blade) and the iliacus muscle (on the dorsomedial side of the blade) in *Sthenurus* are, respectively, 1.8 and 1.6 times the amount of insertion area in *Macropus*. Flared iliac blades, indicating enlarged gluteal muscles, are common among large mammals that engage in bipedal browsing (see [37]). They [12] also note that the iliopectineal tuberosity (at the base of the iliac spine), the area of origin of the rectus femoris, is larger in *Sthenurus*: the acetabulum is also larger in *Sthenurus*, and the acetabulae are placed further apart, resulting in a more wide-legged stance.

Macropodines in general also have a fairly narrow tip of the ilium (tuber coxa), while this is broad in sthenurines, and narrower in species of *Macropus* than in most other kangaroos. The sthenurine condition is approached only by tree-kangaroos (*Dendrolagus* spp.) among macropodines, while other more open-habitat, fast hopping kangaroos such as the nail-tail wallabies (*Onychogalea* spp.) and hare-wallabies (*Lagorchestes* spp.) parallel the *Macropus* species in having narrow tuber coxae (see Figure S1A). While there is debate about the systematic position of the nail-tail wallabies, the molecular data [7] show that both *Onychogalea* and *Lagorchestes* are independent radiations to *Macropus* within the Macropodinae: they can thus serve as a comparison to *Macropus* species for considerations of morphological features related to greater cursoriality. The tuber coxae serve as the area of origin of the gluteus superficialis and the cranial head of the caudofemoralis in *Macropus* [36], but not in *Setonix* and *Dendrolagus*, where these muscles originate from the thoracolumbar fascia [38]. The gluteal and caudofemoralis muscles act to extend the hip: they may be important in elevating the body over the hip when the feet are on the ground, and would also enable supporting the body over a single hind leg. In all kangaroos the sartorious muscle originates from the tuber coxa, and the medial and deep gluteals originate from the iliac blade [38].

The larger species in the genus *Macropus* have elongated ischia (dorsal length of ischium around 65–70% of the length of the ilium), with a pronounced posteroventral projection to the bone. This long ischium provides an elongated moment arm for the muscles that retract the femur, both the hamstring complex and the adductor complex. Elftman [35] notes that the elongation of the ischia effectively turns the action of the adductors into femoral retractors (i.e., hip extenders). This is obviously advantageous for powerful hip extension during rapid locomotion, paralleled among placentals by the extension of the ischia in the cheetah (*Acinonyx jubatus*) in comparison with less cursorial felids [39]. However, this condition is derived in *Macropus* among the other macropodoids, which have a relatively shorter ischium (less than 60% the length of the ilium), although the nail-tail wallabies (*Onychogalea* spp.) have also elongated their ischia, to an even greater percentage of the ilium (>80%) than in *Macropus* (see Figure S1B). This increased length of the pelvis caudal to the acetabulum can be seen even more clearly in a comparison of the length of the puboischiatic symphysis (Figure S1C).

**Figure 3:**
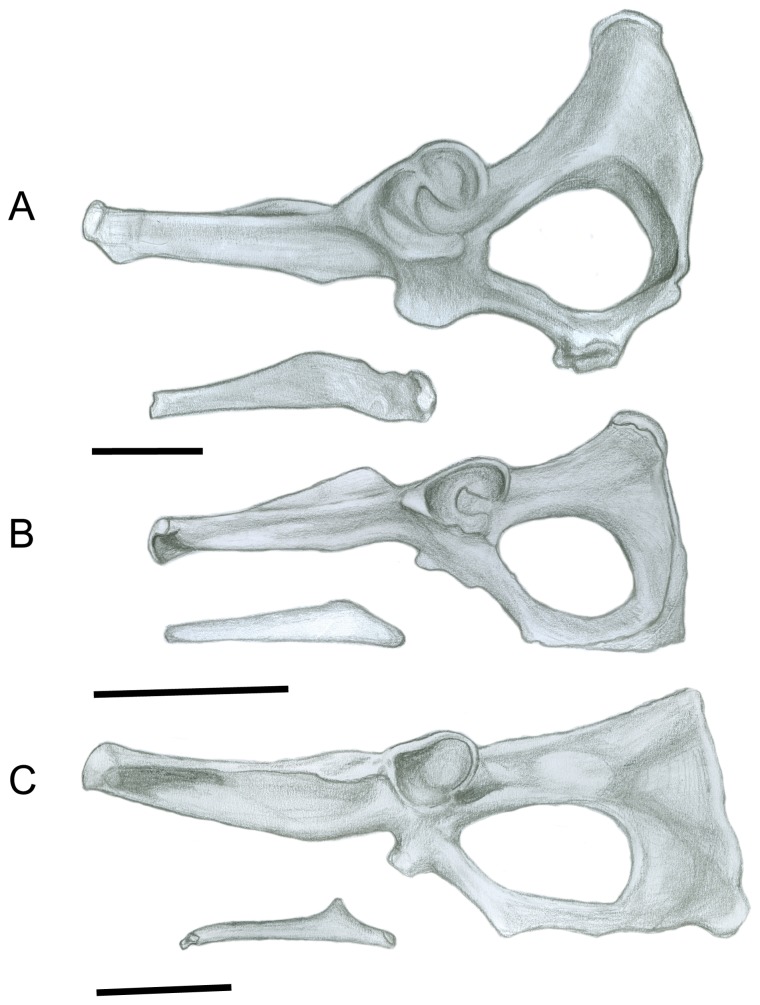
Pelvis. (A) *Simosthenurus occidentalis* (SAM: P17358). (B) *Dendrolagus dorianus* (SAM: M9190) (C). *Macropus robustus* (SAM: M3695). Left lateral view. Scale bar  = 5 cm.

However, sthenurines have ischia that are markedly different to those of other macropodids, approached (convergently) only among the tree-kangaroos. The ischia are only somewhat shorter than in the regular macropodid condition, but are tipped dorsally: Wells and Tedford [12] note that the angle between the ilium and the ischium is 170^o^ in *Macropus*, but 145^o^ in *Sthenurus*. This difference in anatomy results in a markedly different shape of the obturator foramen, which is elongated and ovoid in *Macropus*, moderately oval in most other macropodoids, and circular/triangular in sthenurines (and also in *Dendrolagus* spp.) (see Figure 3). This dorsal tipping of the ischium markedly repositions the moment arm for the hamstring muscles, especially for the biceps femoris, which originates from the ischial tuberosities [36]. Wells and Tedford [12] note that the ischial anatomy in sthenurines would increase the area of origin of the quadratus femoris, which acts as a femoral adductor, and this could be important in preventing the legs from spreading when standing upright.

A notable difference in the pelvic area is in the size of the epipubic bones (see Figure 3). The epipubics of *Sthenurus* are almost as long as the ilium, but are no more than half the length of the ilium in *Macropus* [12]. The epipubics of *Macropus* are possibly somewhat reduced over the primitive macropodoid condition, but in *Dendrolagus* spp. the epipubics are of similar relative size to those of sthenurines (see Figure S1D). The prime function of the marsupial epipubic bones, to which the pectineus, pyramidalis and hypaxial muscles attach, is to stiffen the trunk during locomotion: the epipubics act part of a kinetic linkage between the femur and the hypaxial muscles, resisting torsion and diagonal stress across the trunk [40].

#### Femur (Figure 4)

**Figure 4:**
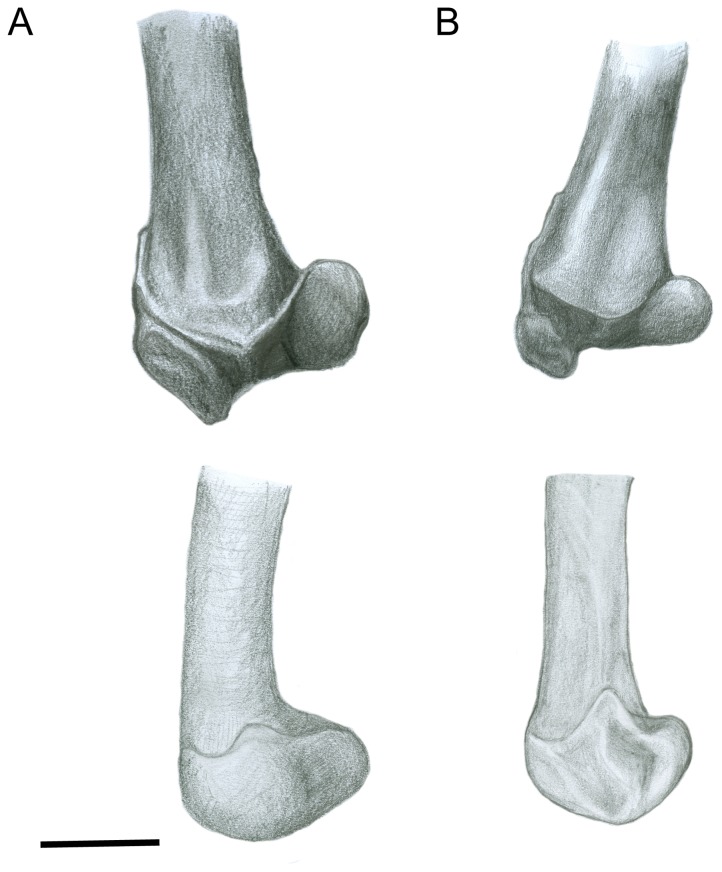
Femur. (A) *Simosthenurus occidentalis* (SAM: P17259). (B) *Macropus* sp. (SAM: P17270). All left side: upper  =  proximal articular view; lower  =  lateral distal view. Scale bar  = 5 cm.

The head of the femur is proportionally large in sthenurines (matching the enlarged acetabulum, see above): tree-kangaroos (*Dendrolagus* spp.) also have relatively large femoral heads, as do Protemnodon spp. (see Figure S2A). The shape of the femoral head is round in sthenurines, rather than ovoid as in *Macropus* (see Figure 4). The more ovoid morphology of *Macropus* is the derived one among macropodoids, and is likely related to restricting femoral motion to a parasaggital plane, as also seen among cursorial bovids [41]. The neck of the femur is also elongated in *Macropus*, which may increase the moment arm for the gluteal muscles, again reflecting cursorial adaptations.

The femoral shaft is curved in both *Macropus* and sthenurines, but the orientation of the femur is slightly different, so that the knee points medially in *Macropus* and laterally in sthenurines [42]. The greater trochanter, the major point of insertion of the gluteal muscles, is large in both *Macropus* and the larger sthenurines: this may relate partly to body size, as smaller sthenurines (e.g., *S. andersoni*) have a proportionally smaller greater trochanter [12]. However, Wells and Tedford [12] also note that the greater trochanter is relatively longer, and more closely aligned with the axis of the femoral shaft, in *Sthenurus*. This echos the point made above, that a larger volume of gluteal musculature would enable sthenurines to balance their body weight over one leg: in humans, larger superficial gluteal muscles are important in preventing collapse of the body medially when the weight is borne on one leg [43].

Wells and Tedford [12] note that the lesser trochanter is “weaker” in *Sthenurus*, but it is placed more distally on the femoral shaft in sthenurines than in other macropodoids (although tree-kangaroos are more like sthenurines), increasing the moment arm for the iliopsoas (see Figure S2B). Note that the area for the origin of the iliacus on the medial surface of the iliac blade is also greater in sthenurines. It is not completely clear what the function might be of a more powerful iliopsoas in sthenurines: it may relate to different mechanics of femur protraction if the femur is being held in a more vertical position (i.e., with an upright trunk), because the moment arm of the iliopsoas would be less favorable with a limb in this position.

As noted by Wells and Tedford [12] the adductor scar on the posterior part of the femur in *Sthenurus* is placed more distally than in *Macropus*. This marks the insertion point of the quadratus femoris ( =  ischiofemoralis), which originates from the ischium and is essentially the most proximal component of the adductor musculature complex. Hopwood and Butterworth [36] note that this muscle is tendinous in *Macropus*, and suggest that it acts as a form of check ligament to prevent overflexion of the hip during jumping (i.e., to limit the forward motion of the femur). Without knowing the more general condition for this muscle in all macropodids it is impossible to even guess whether or not this muscle was tendinous in sthenurines. The longer moment arm afforded by the more distal insertion on the femur might be indicative of more powerful limb retraction and abduction, or it might simply be the case that the different morphology of the sthenurine ischium (see above) changed the previous nature of the moment arm, and the more distal placement of the insertion is merely compensating for this. Tree-kangaroos (*Dendrolagus* spp.) also have a relatively distally placed adductor scar (see Figure S2C), and a correspondingly large quadratus femoris muscle [38].

Wells and Tedford [12] suggested that the articular facets on the *Sthenurus* distal femoral condyles would allow for a greater range of knee motion than in *Macropus*. They also noted that the lateral femoral condyle is markedly larger than the medial one in both *Macropus* and *Sthenurus*. However, both condyles are elongated in the anteroposterior direction in sthenurines, giving them a more elliptical shape than in *Macropus* (see Figure 4). More importantly, sthenurines have a greater width across the distal femoral condyles than do macropodines: that is, they have relatively bigger knees, as well as relatively bigger hip joints (see Figure S2D). Tree-kangaroos also have relatively broader knees than other macropodines (see Figure S2D).

#### Tibia and Fibula (Figure 5)

All macropodids have relatively long tibia, up to twice the length of the femur. Tibial length scales with positive allometry in macropodids in general [3], [11]. Tibia lengths are shorter in *Dendrolagus* spp. and taxa that rely on more quadrupedal (pentapedal) locomotion, such as the New Guinea forest-wallabies (*Dorcopsis* and *Dorcopsulus* spp.). Sthenurines have tibiae of comparable lengths to generalized macropodids, but the extinct *Protemnodon* spp. have relatively short tibiae [11].

On the proximal tibia, Wells and Tedford [12] note that the lateral and medial condyles are of approximately equal size in *Sthenurus* and *Macropus*, but did not comment on the elongation of the tibial tuberosity in *Macropus* (which is derived relative to other macropodoids) (see Figure S3A). The enlarged tibial tuberosity of *Macropus* goes along with the greater size of the proximal portion of the tibial (cnemial) crest. Murray [42] notes that the macropodines and sthenurines differ in tibia diaphysis: macropodines have a sharply defined tibial crest that is limited to the proximal quarter of the bone, terminating in a distinct notch, and the anterior profile of the tibia is straight; sthenurines have a elongated crest that is convex in profile, and blends into the more distal shaft, and the anterior profile of the tibia tends to be sinuous (especially in *Procoptodon*). By comparison with the potoroine condition, Murray [42] concluded that it is the macropodine condition that is the derived one. The tibial crest serves as the insertion point both for the tibalis anterior (which flexes the foot) and for the gracilis, which abducts the leg [36]. A shorter, more prominent tibial crest would concentrate the origin of the tibialis anterior proximally, and may relate to the ability for more rapid foot flexion.

**Figure 5:**
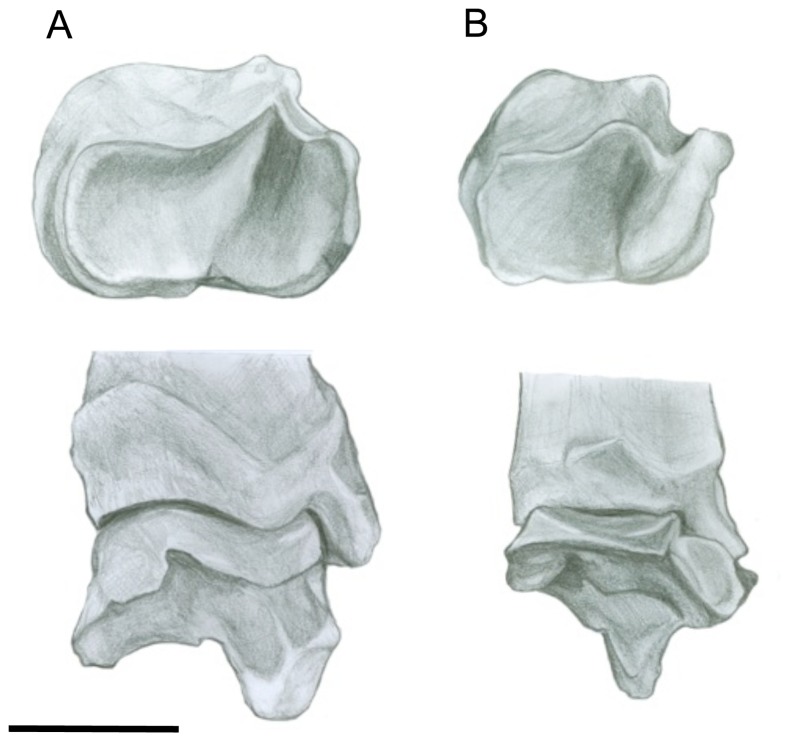
Tibia. (A) *Procoptodon goliah* (NMV2010). (B) *Macropus giganteus* (AMNH 2390). All left side: upper  =  distal articular view (plantar side upwards); lower  =  posterior (plantar) view, showing articulation with tarsus. Scale bar  = 5 cm.

On the distal tibia, Murray [42] notes that the tibioastragalar joint is rotated in an anteriomedial direction in sthenurines, which he ascribes to a compensation for the outwardly-rotated knees, and a morphology which would rotate the feet to be in a more medial position. Wells and Tedford [12] note a longer and more robust medial malleolus in *Sthenurus* than in *Macropus*, and an articular groove that is more of an “oblate cup” in shape than the “shallow, arcuate” form in *Macropus*. They comment that this morphology would mean a more constrained tibioarticular articulation in *Sthenurus*, but they do not specifically note a unique morphology of the sthenurine distal tibia: that is, of a plantar process that fits in a tongue-in-groove linkage into the astragalar trochlea (see Figure 5). This morphology can also be observed in the Miocene sthenurine *Hadronomas* (NT 2469: personal observation of senior author).

With regards to the fibula, Wells and Tedford [12] note that *Sthenurus* lacks the distinctive posterior process of the head of the fibula seen in *Macropus*, which apparently allows for greater flexion of the knee. They conclude that this morphology may relate a longer groove in the proximal tibia for the insertion of the fibula, and interpret this as allowing for a greater internal rotation of the lower limb about the knee. They relate this to the notion of to sthenurines needing to achieve greater limb rotation to position their feet medial when landing while hopping, as their wider pelvis would otherwise result in more lateral placement of the feet. However, this could also relate to a rotation of the body around the knee when the foot was placed on the ground, as would be experienced with bipedal striding.

#### Astragalus and Calcaneum (Figure 6)

**Figure 6:**
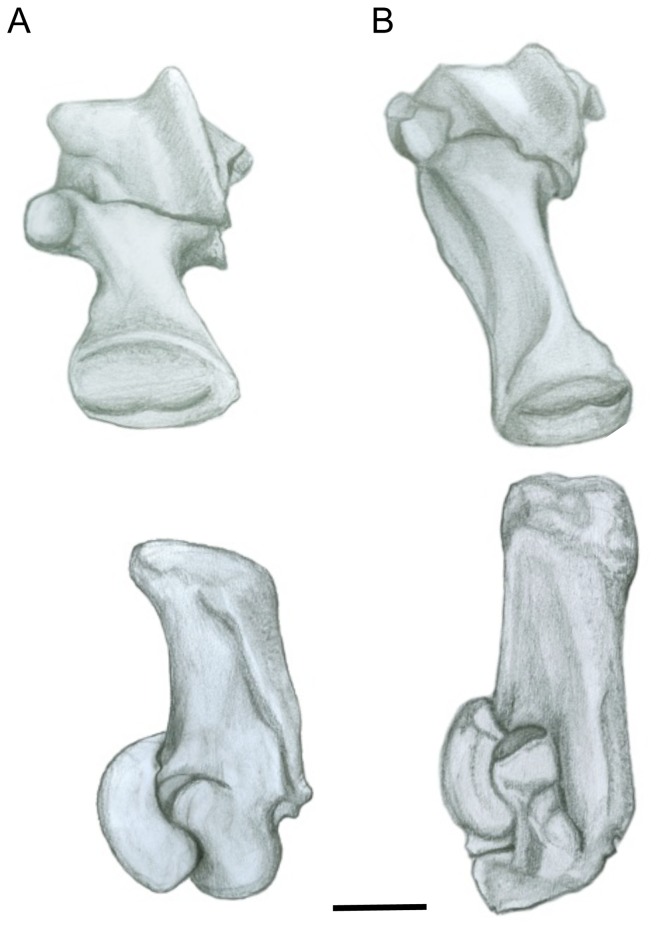
Astragalus and calcaneum. (A) *Simosthenurus occidentalis* (SAM: P17258 [reversed]). (B) *Macropus giganteus* (SAM: P17523 [reversed]). All left side: upper  =  proximodorsal view; lower  =  lateral view. Scale bar  = 2 cm.

In concert with the tongue-in-groove fit of the distal tibia into the astragalus, the astragular trochlea groove is much deeper in sthenurines than in any other macropodoid, with high medial and lateral ridges (see Figure 6). Wells and Tedford [12] note that the axis of the astragalus differs between *Sthenurus* and *Macropus*: the astragalus is at a right angle to the longtitudinal axis of the pes in *Sthenurus*, but is rotated medially in *Macropus* by as much as ten degrees, and with a less robust connection of astragalus and calcaneum than in *Sthenurus*. A greater degree of rotation of the axis of the astragalus is seen in other macropodoids, in particular *Dendrolagus* spp. (see [44], [45]). Both the morpohology and the degree of angulation of the astragalus in sthenurines appear to be derived features among macropodids, also seen in the Miocene sthenurine *Hadronomas* [42], and to a certain extent in the smaller and more primitive Miocene sthenurine *Rhizosthenurus* [16]. The sthenurine orientation of the axis of the astragalus is likely related to a foot more restricted to anteroposterior motion, with compressive stresses being directed more anteroventrally [12]. Bishop [44] also notes a much greater prominence in sthenurines of the points of insertion of the ligaments binding the astragalus and calcaneum.

*Macropus* also has an astragalus that is elongated in the proximodistal direction, which may relate to a greater excursion of the tibia over the foot during rapid hopping. This is not paralleled in other more cursorial kangaroos. Sthenurines have an especially highly raised medial trochlear ridge, paralleling the condition in horses, and a long lateral trochlear ridge (see Figure 6) (both noted as being derived features by Murray [42]). The fibular facet on the astragalus in sthenurines, which is on the side of the lateral trochlear ridge, is also broader in sthenurines than in other macropodids (see Figures 6, S3B).

Macropodines in general also have a fairly broad medial malleolar process (for the reception of the medial malleolus of the tibia). This process is large in *Dendrolagus* spp., but more restricted in *Macropus*, and narrower still in sthenurines: this goes along with the derived sthenurine feature of a larger and more posteriorly-directed navicular facet [42]. This morphology appears to be related to intratarsal mobility, being greater in *Dendrolagus* spp., somewhat restricted in *Macropus*, and even more restricted in sthenurines. Murray [42] also notes the derived condition in sthenurines of a more mesially directed and proximally short astragalar head.

In the calcaneum, *Macropus* has a much longer calcaneal tuber than that of *Sthenurus*, and the orientation differs: the calcaneal tuber in *Macropus* is orientated straight up and slightly posteriorly, while in *Sthenurus* it is curved slightly forwards (see Figure 6). A relatively short calcaneal tuber is the generalized condition in macropodines, and longer tubers have been evolved convergently in more cursorial kangaroos, such as *Petrogale*, *Lagorchestes*, and *Onychogalea* (see Figure S3C). A deep body to the tuber (in the dorsoventral plane) is also a sthenurine feature: Murray [42] notes that most macropodoids have a tuber that is either shallow at the base or, as in *Macropus*, broad at the base but tapering towards the tip, but is unable to determine the polarity of this feature. However, a very distinctive and derived sthenurine feature is the great broadening of the tip of the tuber in the mediolateral direction (which is paralleled to a certain extent in *Dendrolagus* spp.) (see Figure 6). In *Dendrolagus*, this calcaneal morphology is associated with a short Achilles tendon [45]. Bishop [44] notes this feature, but also notes that the more proximal portion of the tuber in sthenurines is relatively narrow in the mediolateral plane, and concludes that this indicates forces acting upon the calcaneal tuber as being largely in the sagittal plane, implying less adaptation to fast locomotion in sthenurines than in *Macropus*.

The calcaneal tuber serves as the insertion of the gastrocnemius muscle, and also for a portion of the semitendinosus (at least in *Macropus giganteus* [36]), via the Achilles tendon. A longer calcaneal heel will increase the moment arm of the gastrocnemius: this will not only provide more power for the hop, but will also allow for a greater amount of elastic energy storage in the gastrocnemius tendon.

The sulcus on the medial side of the calcaneum for the flexor digitorum longus is fairly broad in most macropodids, especially in *Dendrolagus* spp., but is more constricted in sthenurines (see [42]). The transverse plantar sulcus, on the lateral side of the calcaneum, is relatively narrow in sthenurines, reflecting the generalized macropodid condition. This sulcus is broader in *Macropus* and other cursorial kangaroos such as *Onychogalea*; it houses the tendon of the peroneus longus, which runs from the craniolateral shaft of the fibula, as it passes over the calcaneum to the plantar surface of the foot to insert on the lateral surface of the first metatarsal. Its action appears to be to both flex the ankle and to counteract the action of the tibialis anterior in preventing evulsion of the pes [46]. The convergent morphology between *Macropus* and *Onychogalea* indicates that this anatomy relates functionally to more rapid and/or sustained hopping, and may reflect increasing need for the control of the foot position on landing with frequent and rapid limb return.

The fibular facet on the calcaneum is more prominent in *Macropus* than in *Sthenurus*, and is also located in a more medial position on the tarsus (see Figure 6). The generalized macropodoid condition appears to be for a facet that is less prominent, but located in a medial position as in *Macropus*. The facet is more prominent in sthenurines than in many macropodids, which suggests that the facet has been displaced laterally in sthenurines. Bishop [44] interprets the position of the sthenurine fibular facet as enabling greater ability to pronate the foot, which could be important in terms of bearing weight on the medial side of the foot, and notes that this motion would necessitate the observed sthenurine morphology indicating powerful ligaments binding together the astragalus and calcaneum. Murray [42] also interprets a suite of astragalocalcaneal features as relating to bearing weight more on the medial side of the foot (including the plantar crest of the calcaneum being more elongated on the medial side), seen in *Hadronomas* as well as in more derived sthenurines. However, if both feet were habitually landing on the ground at the same time, as in almost all other macropodoids, there would be little need to favor one side of the foot for weight-bearing. Humans (as opposed to apes) have a suite of morphological adaptations related to the shifting of their weight to the medial side of the foot during locomotion [43]. This feature of sthenurines may again be indicative of weight bearing on one leg at a time.

The sustentaculum tali, on the medial side of the calcaneum, is where the weight of the animal's body is transmitted from the astragalus to the calcaneum, and from there to the foot. The sustentaculum is taller in the dorso-plantar direction in both *Macropus* and *Sthenurus* than in other macropodids, giving the calcaneum an asymmetric “hunched shoulders” appearance when viewed from the plantar side. A larger sustentaculum would indicate greater capacity for weight transmission, either from a larger body size and/or from greater forces encountered in rapid locomotion. However, the shape of the sustentaculum is notably different in sthenurines than in macropodines. Bishop [44] notes that it is narrower (in the mediolateral direction) in sthenurines, and proposes this as part of a suite of adaptations that allow for plantar flexion when the foot is internally rotated. In medial aspect, the sustentaculum is dorsoplantarly deep and right-angled in sthenurines, while in macropodines it is narrower with the distal border orientated at a 45^o^ angle. Bishop [44] interprets this morphology as preventing the medial dislodgement of the tendon of the flexor digitorum longus (which passes over the top of the sustentaculum), which could be important in the act of elevating the foot to stand on the toes (i.e., in moving from a plantigrade, resting, posture to a digitigrade, locomotor posture).

On the distal calcaneum, all macropodoids have a “stepped” cubonavicular facet, with the dorsolateral facet being projected more ventrally than the dorsomedial or ventromedian facets. This morphology has been considered important as an adaptation for hopping, in limiting movement between the calcaneum and the cuboid [44]; but note that this morphology is also seen in the musky rat-kangaroo (*Hypsiprymnodon moschatus*), which is considered to be a primarily non-hopping form [21], and is retained (although reduced) in *Dendrolagus* spp. [44], [45]. A stepped calcaneum is also seen among the balbarids, supposedly non-hopping macropodoids [47]. Sthenurines retain the more generalized macropodoid condition, while in *Macropus* the “stepping” is more pronounced and the dorsolateral facet is wider in the mediolateral direction.

In distal view, the generalized macropodine condition of the calcaneum (as seen, for example, in *Dorcopsulus* and *Setonix*) is for equal-sized dorsolateral and dorsomedial facets, with a relatively narrow (in the dorsoplantar direction) ventromedian facet giving the distal surface a rectangular profile. In *Dendrolagus* the ventromedian facet is indistinct and merged with the dorsolateral facet, a morphology that Warburton and Prideaux [45] interpret as allowing for a greater amount of inversion and eversion of the foot than in terrestrial kangaroos. In sthenurines all of the facets, and especially the ventromedian facet, are elongated in the dorsoplantar direction (i.e., a large measure C21 as seen in [Fig pone-0109888-g007] [Fig pone-0109888-g008] Figure 9K), resulting in a square profile of the distal surface. Interestingly, the large species of *Protemnodon* did not broaden the ventromedian facet in this fashion (e.g., in Flinders University specimen 1611; personal observation of senior author), suggesting that large size alone is not the reason for this change in morphology.

**Figure 7 pone-0109888-g007:**
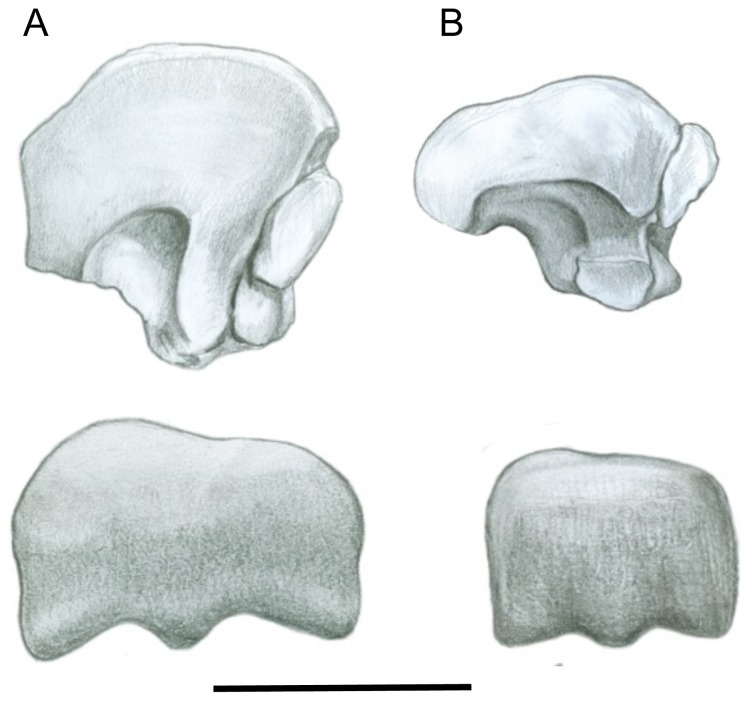
Fourth metatarsal. (A) *Sthenurus stirlingi* (AMNH 117496). (B) *Macropus giganteus* (AMNH 2390 [reversed]). All left side: upper  =  proximal articular view; lower  =  distal articular view (plantar side downwards). Scale bar  = 2 cm.

**Figure 8 pone-0109888-g008:**
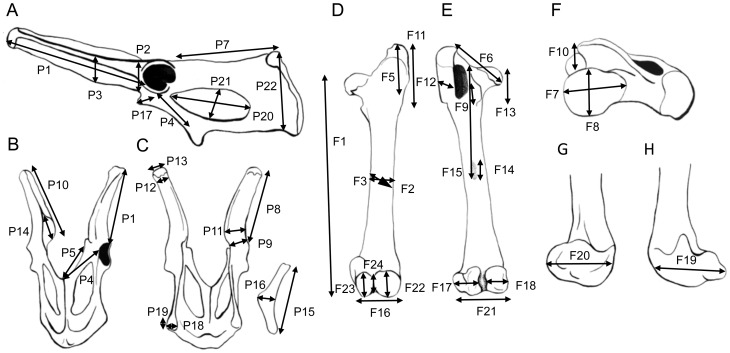
Measurements used in analyses-1. Drawings, all of left side elements, primarily from photographs of *Macropus fuliginosus*, AMNH 2390. (A) Pelvis, lateral view. (B) Pelvis, ventral view. (C) Pelvis, dorsal view. (D) Femur, anterior view. (E) Femur, posterior view. (F) Femur, proximal articular view (anterior of shaft downwards). (G) Distal femur, medial view. (H) Distal femur, lateral view. A detailed description of the measurements is provided in Table S2.

**Figure 9:**
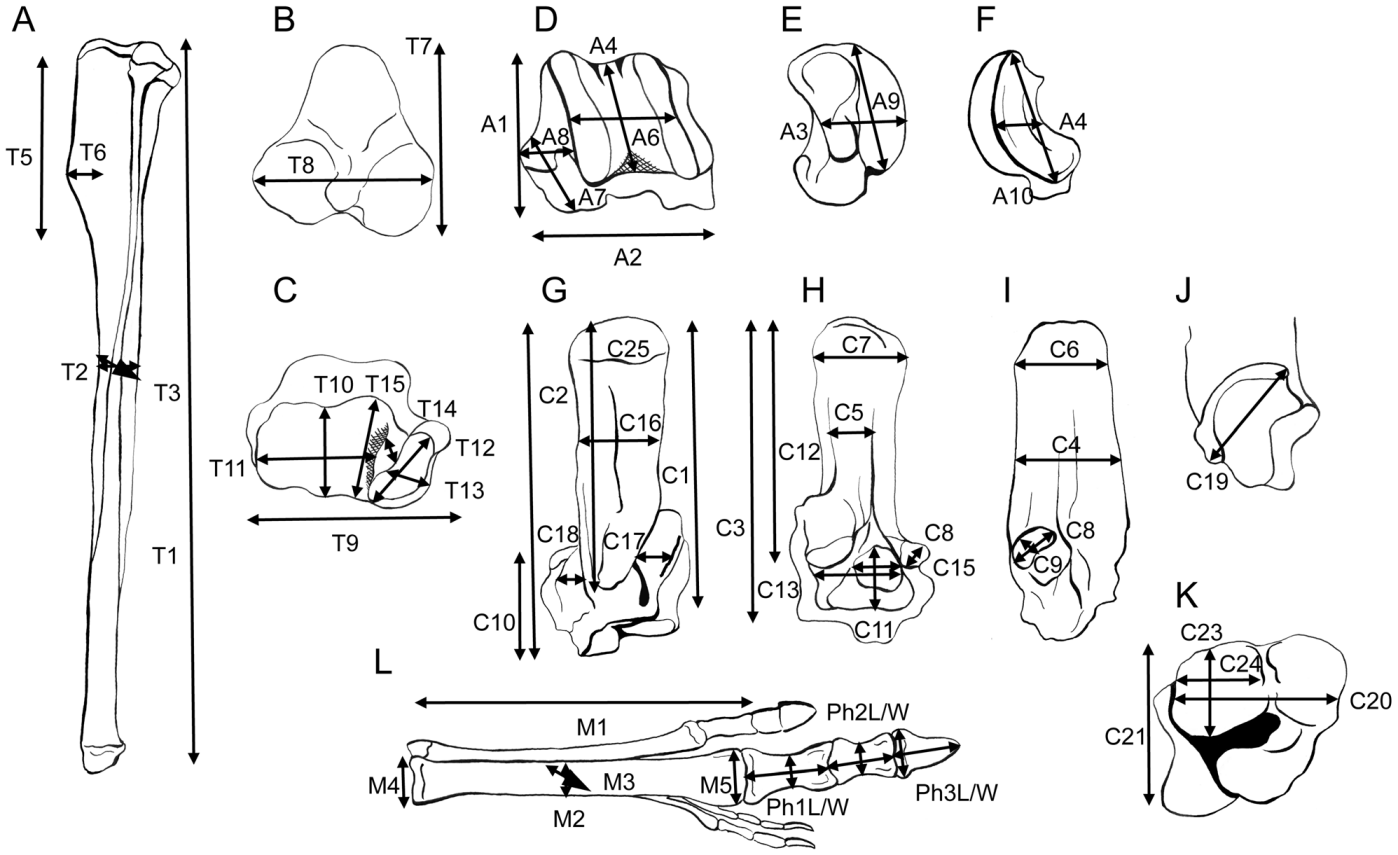
Measurements used in analyses-2. Drawings, all of left side elements, primarily from photographs of *Macropus fuliginosus*, AMNH 2390, Calcaneum from *Macropus giganteus*, AMNH 74753). (A) Tibia, lateral view (fibula removed). (B) Tibia, proximal articular view (plantar side downwards). (C) Tibia, distal articular view (plantar side upwards). (D) Astragalus, anterior (dorsal) view. (E) Astragalus, medial view. (F) Astragalus, lateral view. (G) Calcaneum, anterior (dorsal) view. (H) Calcaneum, posterior (plantar) view. (I) Calcaneum, lateral view. (J) Calcaneum (head only) medial view. (K) Calcaneum, distal articular view (plantar side upwards). (L) Pes, anterior (dorsal) view. A detailed description of the measurements is provided in Table S2.

#### Metatarsals and Phalanges (Figure 7)

The fourth metatarsal is long and curved in *Macropus*, with a prominent posterior bulge (the plantar crest) that extends down the proximal third of the bone. The metatarsals are also curved in *Sthenurus*, but appear to be proportionally somewhat shorter, and Wells and Tedford [12] note that the cross-sectional area in *Sthenurus* is from 1.2 to 1.5 times larger than would be predicted for a *Macropus* of similar size. The plantar crest is deeper in sthenurines and extends further down the length of the bone. This crest serves as the insertion for the origin of interosseus muscles, and is reduced in placentals with an unguligrade stance where these muscles have been reduced to ligaments [48].

The generalized macropodine condition, as seen for example in *Dorcopsulus* and *Setonix*, is for relatively short metatarsals; the metatarsals of sthenurines are elongated over this general condition, but they tend to be shorter than those of *Macropus* and other more cursorial macropodines such as *Petrogale*, *Onychogalea*, and *Lagorchestes* (see Figure S3D), where this morphology has evolved convergently several times [42]. The metatarsals of tree-kangaroos (*Dendrolagus* spp.) have been shortened, more so in the New Guinea species than in the Australian ones [45]. *Protemnodon* spp. also have fairly short metatarsals.

The proximal articular surface of the fourth metapodial (articulating with the cuboid) has a fairly flat anterior profile in most macropodoids, while in sthenurines this surface curves posteriorly both medially and laterally, resulting in a more plantar (versus more lateral) positioning of the intra-articular groove [42]. The facets for the articulation with the ectocuneiform in the tarsus are larger in sthenurines than in macropodines (see Figure 7A). For a similarly sized bone, the proximal articular surface is about 50% larger in *Sthenurus* than in *Macropus* [12], and the plantar eminence forms around one third of the width of the posterior proximal surface, but less than 20% in *Macropus*. Sthenurines exhibit a derived condition for macropodids for all of these features (see Figure 7). All of these differences between sthenurines and macropodines indicate a relatively larger ankle joint in sthenurines.

The distal articular surface of the fourth metapodial is fairly square-shaped in most macropodoids, but in sthenurines not only is the distal surface proportionally larger, but it is more mediolaterally elongated (see Figure 7). This is reflected in the rectangular profile of the proximal articular surface of the proximal phalanx of the fourth pedal digit [42]. Sthenurines also have a less prominent distal metapodial keel than *Macropus* [42]. This might reflect a greater amount of movement of the phalanges on the metapodial (see [49]), although the potential functional reasons for this are unclear.

The phalanges of sthenurines are distinctive: the proximal and middle phalanges appear “waisted”, with expanded proximal and distal ends, but a narrower median portion. The proximal phalanx of *Macropus* is relatively long, almost twice the length of the second phalanx, while in *Sthenurus* the second phalanx is around 80% of the length of the first. In comparison with other macropodids, this appears to represent a relative enlargement of the medial phalanx in *Sthenurus*, in addition to the lack of lengthening of the proximal one as seen in *Macropus*. The generalized macropodid condition is for a relatively short proximal phalanx, with elongation seen convergently in more cursorial kangaroos, such as *Petrogale, Onychogalea*, and *Lagorchestes*, while the proximal phalanx is shorter and broadened in *Dendrolagus* spp. A lengthened proximal phalanx is also seen in more cursorial ungulates (hoofed placentals), where it apparently relates to a lengthening of the plantar tendons, increasing the “springiness” of the foot [50].

The ungual phalanx is curved and rather claw-shaped in most macropodoids, being narrow in the mediolateral direction. More distinctive claws are seen in the species of *Dendrolagus*. In contrast, the ungual phalanx in sthenurines is more blunt and rounded, described as “hoof-like” by Kear et al. [51]. Similar ungual phalangeal morphology is seen in the earlier (Miocene) sthenurines *Hadronomas* and *Rhizosthenurus*, and convergently among *Protemnodon* spp. [51].

Wells and Tedford [12], and Murray [42], note well-developed scars for the plantar cruciate ligaments of the pes in *Sthenurus*: Murray [42] postulates that these scars, that are more separated and powerful than those in other macropodoids (and also apparent in *Hadronomas*), represent more torque being placed on the first phalanx.

### Speculations on sthenurine locomotor anatomy

Despite the potential biomechanical problems of hopping locomotion in large kangaroos, the ability of sthenurines to use hopping as a mode of locomotion has rarely been questioned (but see [2], [31]). Sthenurines possess the elongated hind limbs (with an especially long tibia) seen in extant large kangaroos, which have been interpreted as an adaptation for hopping [11], [12], but this anatomy was inherited from their macropodid ancestry: many sthenurine species were of such large size that the biomechanics of hopping are rendered implausible [2], [31].

One possible reason for the lack of questioning about the mode of locomotion of sthenurines is the issue of monodactyly. This has been seen as analogous to the attainment of monodactyly in the equid lineage, and thus indicative of a highly cursorial lifestyle, rendering sthenurines even more specialized hoppers than the large macropodines (see [52], p. 59), although Wells and Tedford [12] later emended that conclusion to perceiving sthenurines as being slow hoppers. However, despite their monodactyl pes, other skeletal modifications of sthenurines are less indicative of adaptations for faster locomotion: we later propose an alternative, non-locomotor, explanation for monodactyly in sthenurines.

The apparent “robusticity” of sthenurines has often been noted, but is not well understood. Wells and Tedford [12] proposed that the robusticity relates to them having to support their “great weight” in a bipedal browsing posture, where they are envisaged to have risen up on their hind legs, gerenuk-style, and reached over their heads with their arms. However, why should it be the case that sthenurines were so much more heavily build than macropodines of similar linear dimensions? A speculation that has been raised is that, because sthenurines were browsers, they had to support a massive gut (and this reason is also given for the larger distance between the pelvic acetabulae and for the massive epipubic bones in sthenurines) (e.g., Wells and Tedford [12]). But this hypothesis is problematic: not only are kangaroos foregut fermenters (so any “massively enlarged” portion of their gut would be well anterior to the pelvic region) but, in ungulate placental mammals at least, it is the gut fermentation areas of grazers, rather than browsers, that are the more massive [53]. Consider the black rhino (*Diceros bicornis*, a browser) and the white rhino (*Ceratotherium simum,* a grazer): they are of a similar size (although the black rhino is slightly smaller), but there is no indication that there is any difference in the relative robusticity of their skeletal framework. Thus, on the basis of diet alone, one would expect the large grazing species of *Macropus* to be the more robust forms, which is clearly not the case. In addition modern kangaroos show little capacity for gut expansion [54].

A clue to this issue of “robusticity” may be found in the proportions of a third lineage of kangaroos to reach “giant” proportions: species of the extinct macropodine *Protemnodon*. Large species of *Protemnodon* showed similar robusticity to the sthenurines, despite a very different postcranial anatomy to either sthenurines or large species of *Macropus* (and with craniodental anatomy indicative of a mixed-feeding diet). This raises the issue of what is the normal allometric scaling for kangaroos: we will discuss later the likelihood that the real issue is that it is the large species of *Macropus* that are relatively gracile, with sthenurines and *Protemnodon* spp. representing the “normal” condition.

Another hypothesis for the more massive hind limb structures in sthenurines is related to the supposed browsing posture, where the animal must raise its body up over the hips (see [12], p. 78]. But why would this feeding posture necessitate more robust limbs? The weight of the animal would be the same whatever its posture (although admittedly some of the directional forces would be different). It seems more likely that some sort of dynamic forces applied during locomotion would be the issue necessitating more sturdy support. The hopping gaits of extant kangaroos mean that both hind feet are always applied to the ground at the same time. In human running the vertical ground reaction forces applied to each foot on landing are between two and three times the body weight [55]. Does kangaroo-style hopping mean that this reaction force is evened out over both hind feet? If so, perhaps more robust limbs reflect a locomotor shift to bearing weight on one foot at a time.

While Wells and Tedford [12] did not question the notion of hopping in sthenurines, they did note that the slow, pentapedal gait of macropodines was likely impossible. As a consequence of the modification of the forelimb for browsing in sthenurines, there was limited ability for dorsiflexion of the hand, so they would have had difficulty in placing their hand on the ground in the requisite palmigrade position. The hands are also highly specialized, with extremely long third phalanges, and they appear unsuited to weight bearing. *Sthenurus* also has a smaller olecranon process for the insertion of the triceps than large species of *Macropus*, which Wells and Tedford [12] interpreted as limiting the ability to support the anterior body weight over the hands, or to provide propulsion with the forelimbs. The anterior caudal vertebrae in *Sthenurus* have reduced processes for muscle attachment in comparison with *Macropus* [12], implying less tail musculature and perhaps a tail that is no longer used to propel the body as in pentapedal locomotion. Also note that pentapedal locomotion involves considerable flexion of the backbone, which appears to be limited in sthenurines (see below). A problem thus arises when considering sthenurine locomotion over a variety of speeds. If they just had a “greater dependence on bipedal saltation”, as proposed by Wells and Tedford [12], p. 85, then how did they manage to locomote at slow speeds? As discussed previously, hopping is difficult if not impossible at slow speeds, and large modern macropodines employ pentapedal locomotion up to speeds of around 3 m/sec [18], [28].

We propose here that sthenurine kangaroos employed a novel type of gait, certainly at slow speeds and likely also at faster speeds in larger species: that of bipedal striding with a relatively upright trunk. This gait is not unique among macropodids, as it is occasionally seen in tree-kangaroos, walking along a branch [23]. This is not to imply that sthenurines, especially the smaller ones, never employed a hopping gait, but that the addition of this gait to their locomotor repertoire can explain many of the anatomical peculiarities of these animals. In the previous section we discussed the anatomical differences between sthenurines and large macropodines, and proposed that the differences in sthenurine anatomy from other large kangaroos can be related to supporting the body weight during locomotion over a single hind leg. In following sections we present analyses of anatomical data comparing sthenurines with other kangaroos (both extant and extinct), and discuss how our results support this hypothesis.

## Materials and Methods

### Data

We took linear measurements, taken with digital calipers, of 66 extant kangaroo individuals (belonging to 45 species) and 78 extinct kangaroo individuals (belonging to 18 genera) (see Tables S2–4). All of the specimens measured were housed in accredited museum collections: these include the American Museum of Natural History (New York, NY, USA); The Australian Museum (Sydney, NSW, Australia); the University of New South Wales (Sydney, NSW, Australia); the Queensland Museum (Brisbane, QLD, Australia); Museum Victoria (Melbourne, VIC, Australia); the Western Australian Museum (Perth, WA, Australia); the Northern Territory Museum and Art Galleries (Alice Springs, NT, Australia); the South Australian Museum (Adelaide, SA, Australia); and Flinders University (Bedford Park, SA, Australia).

The measurements included 22 from the pelvis, 24 from the femur, 15 from the tibia, 10 from the astragalus, 9 from the calcaneum (plus an additional 16 for a further analysis), and 14 from the pes (see Table S2 and Figures 8, 9; the original measurements are available in Table S5). We did not take measurements of the fibula because of the rarity of preservation of complete fibulae in the fossil record. Measurements of the fifth metatarsal were taken, but not included in the analyses (to avoid the possibility that sthenurines would be grouped merely on the fact that their fifth metatarsal is vestigial). However, we did determine that there is no reduction of the fifth metatarsal with increasing cursoriality in extant macropodids. In a few instances of missing data from extant taxa (usually involving the distal phalanx of the fourth pedal digit) measurements were added from related and similarly sized individuals (to prevent these incomplete individuals from being excluded from the multivariate analyses; details provided in Table S3). We also created a separate data set with 16 additional calcaneal measurements (with only some overlap with the first one in terms of the individuals sampled). This included 70 individuals: 44 extant forms (including 33 species, again sampling all extant genera), and 26 extinct forms (including 10 sthenurines, eight *Protemnodon* spp., and representatives of the smaller fossil forms mentioned below). (Details of the included taxa are in Table S4, and the original measurements are available in Table S6).

Every genus and almost every species of extant macropodoid was measured. Extinct taxa included not only sthenurines, but also a few other forms. For the purposes of seeing if the morphology of modern kangaroo species fell within the range of the smaller extinct taxa, we included information from the late Oligocene/early Miocene balbarid *Nambaroo gillespieae*, the late Oligocene/early Miocene *Ngamaroo archeri* (Macropodidae *incertae sedis*, possibly basal to macropodids above the level of the potoroines), and the late Miocene macropodine *Dorcopsoides* sp. Taxonomy was taken from Prideaux and Warburton [4]. For the purposes of comparing sthenurines with other large kangaroos, we included information from several species of the Pleistocene macropodine *Protemnodon*. We also included information from two “giant” (i.e., larger than living forms) Pleistocene species of the extant genus *Macropus*: *M. titan* and *M. ferragus*. The details of the specimens measured are presented in Table S3, S4. Some of the specimens used in the multivariate analyses are represented by a composite of different individuals (see Table S3 and figure captions).

### Statistical analyses

We performed both bivariate and multivariate analyses on the linear measurements using the statistical package SPSS v. 19. We created a number of bivariate plots of the different bony elements of the skeleton, primarily to confirm the visual observations discussed in the “Descriptive Anatomy” sections, and these are to be found in the supplementary information, along with some discussion of the distribution of taxa (Figures S1–S3). Certain anatomical variables were plotted against a measurement of that same bone that appeared to correlate best with body mass (as determined from the PCA scores). Thus pelvic elements were plotted again the iliac blade length, femoral elements (plus metatarsal length) against femur length, tibial elements against tibia average cross sectional diameter, and tarsal measurements against astragalus width. These plots are presented for visual inspection, and we have not attempted to demonstrate any statistical significance. However, they clearly show the differences between sthenurines and other macropodoids.

**Figure 10:**
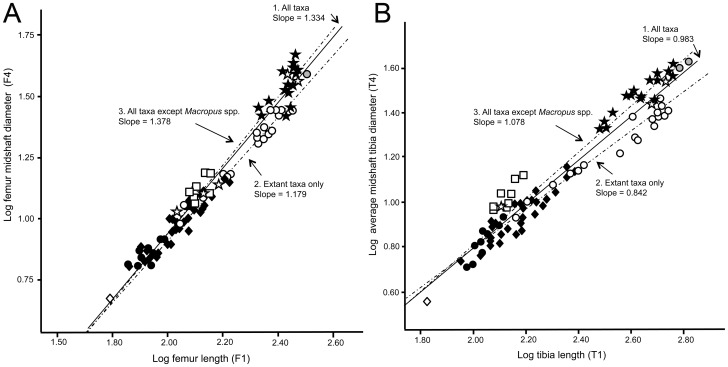
Scaling of long bone length versus diameter (i.e., robusticity). (A) Femur length versus average femur cross-sectional diameter (B) Tibia length versus average tibia midshaft cross sectional diameter. The regression line and its 95% confidence interval (dotted lines) are also shown (A r^2^ = 0.96, B r^2^ = 0.933). Key: Open diamond  =  *Hypsiprymnodon moschatus*; filled circles  =  potoroines; open circles  =  extant species of *Macropus*; half tone circles  =  extinct (“giant”) species of *Macropus* (*M. titan* or *M. ferragus*); filled diamonds  =  extant macropodines (other than *Macropus* or *Dendrolagus*) and lagostrophines; open squares  =  species of *Dendrolagus*; filled stars  =  sthenurines; open stars  =  other extinct taxa. The regression line and its 95% confidence interval (dotted lines) are also shown.

Two of the bivariate plots (Figure 10, showing the scaling of femur and tibia diameter against the length of the same bones), relate to the issue of “robusticity” in larger kangaroos discussed previously, and are shown in the results section. They are also included in Text S1 with a degree of taxa identification (Figure S4), but are shown here without those labels for reasons of clarity. Here three different regression lines were created to examine differences in scaling relationships (in all cases the extinct “giant” species of the extant genus *Macropus* [*M. titan* and *M. ferragus*] were omitted, as their placement in the analyses was more like that of the sthenurines than that of the large extant species of *Macropus*): *(i)* all of the taxa; *(ii)* extant species only; *(iii)* all of the taxa except the extant species of the genus *Macropus*.

As our interest here was in testing for differences in the regression slopes for pair of groups (see above), we performed a Student's t-test between the coefficients (b) of the three regression lines. The null hypothesis (of no difference between the slopes) will be rejected if the regression slopes of each adjusted model are not statistically significant different from each other. In addition, in order to explore if different scaling relationships follow an allometric trend or rather an isometric one, we performed a Student's t-test between the coefficients (b) of each regression line and the theoretical value of a slope equals to one (i.e., the expected coefficient for isometry when two linear measurements are regressed). The null hypothesis (of isometric scaling) will be rejected if the slope of a given bivariate regression model significantly departs from unity, thus suggesting a significant allometric trend.

The multivariate analyses included both Principal Components Analysis (PCA) of log-transformed variables and Linear Discriminant Analysis. These were performed on the following sets of data. *(i)* All hind limb bones combined: this did not allow for the inclusion of many extinct taxa, but three sthenurine species (of the genera *Sthenurus*, *Simosthenurus* and “*Procoptodon*”) could be included, two of which were composite specimens (see Table S3 for details). *(ii)* All hind limb bones except the pelvis: this allowed us to include many more extinct taxa, including species of *Protemnodon*, and the sthenurine genera *Hadronomas* and *Procoptodon*. *(iii)* On the calcaneum alone (using the separate dataset). The calcaneum is a highly informative bone in terms of locomotor behavior [44], [56], but it is usually to be found bound to the astragalus and distal tarsal bones in both extant and fossil specimens: hence our separate analysis from specimens where an isolated calcaneum was available.

All the discriminant analyses were performed by the stepwise approach. This approach was preferred over the direct method because it only uses the best set of variables for discriminating among the groups compared (e.g., [57]–[59]). The selection criterion in the stepwise model was the inclusion of variables with F probability between <0.05–0.01 (depending on sample size and the number of variables), and the exclusion of variables with F probability>0.1. The first analysis was run with an F probability <0.05 of inclusion and, if this analysis included too many variables for the sample size of each specific analysis (see above), we modified the F probability up to <0.01. The F probability for excluded variables was not modified in all of the analyses performed. The power of the discriminant functions was evaluated from the value of the Wilks' lambda statistic (λ), which measures the proportion of the total variance explained by the within-groups differences in the discriminant scores [60]. However, as this statistic provides little information on the effectiveness of the discriminant function for reclassifying the specimens, we assessed the significance of this value by comparing it with the percentage of correct assignments using the leave-one-out cross-validation approach described in Mendoza et al. [57].

## Results

### Bivariate analyses

#### Femur length versus femur diameter (Figure 10A)

The slope for all the taxa (excluding the extinct *Macropus* species *M. titan* and *M. ferragus*, as previously discussed) is 1.334 (slope 1); the slope for the extant species only is 1.179 (slope 2); the slope for all species excluding species of *Macropus* is 1.378 (slope 3). (See Figure S4A for the confidence limits for the regression model and the identification of some of the taxa.) All of these lines are different from isometry (expected slope of 1) at the 95% level of significance, showing that the larger animals have relatively more robust femora (slope 1: *n* = 95; *t* = 10.790; *P*-value <0.0001; slope 2: *n* = 69; *t* = 6.3477; *P*-value <0.0001; slope 3: *n* = 71; *t* = 9.819; *P*-value <0.0001). To a first approximation, slope 2 is the regression line excluding the extinct “giant” kangaroos (which tend to fall above the regression line for all of the taxa), and slope 3 is the regression line excluding the large extant, specialized fast-hopping kangaroos (which tend to fall below the regression line for all of the taxa).

However, the differences between the slopes are interesting. There is no statistical difference between slopes 1 and 3 (*n* = 71; *t* = −0.657; *P*-value = 0.5119): that is the inclusion of the extinct kangaroos does not greatly affect the regression line that fits the extant taxa. However, slope 2 is different from both other slopes at the 99% level of significance (slope 1 vs. slope 2: *n* = 69; *t* = 3.294; *P*-value = 0.001; slope 1 vs. slope 3: *n* = 69; *t* = 3.776; *P*-value = 0.0002); that is, with the exclusion of the large extinct species, the slope is significantly less steep. We interpret this to mean that the femoral proportions of sthenurines and *Protemnodon* spp. are following the “normal” allometric relationships for kangaroos, and that the larger species of *Macropus* are acting to pull the slope down to a lower level.

#### Tibia length versus tibia diameter (Figure 10B)

Here the scatter around the regression line is considerably greater than for the femur proportions, and the 95% confidence limits of the slope are much broader. (See Figure S4B for the confidence limits for the regression model and the identification of some of the taxa). The slope for all of the taxa (excluding the extinct *Macropus* species *M. titan* and *M. ferragus*, as previously discussed) is 0.983 (slope 1); the slope for the extant species only is 0.842 (slope 2); the slope for all species excluding species of *Macropus* is 1.078 (slope 3). Slope 1 is not different from isometry at the 95% level of significance (slope 1: *n* = 92; *t* = −0.595; *P*-value = 0.552). Both slopes 2 and 3 are different from isometry at the 95% level of significance (slope 2: *n* = 66; *t* = −4.7215; *P*-value<0.0001; slope 3: *n* = 69; *t* = 2.777; *P*-value = 0.0071): that is, slope 2 represents negative isometry, and slope 3 positive isometry.

Slopes 1 and 3 are different from each other at the 95% level of significance, while slope 2 is different from both of the other slopes at the 99% level of significance. Thus, with the inclusion of the sthenurines and *Protemnodon* spp. the relative width of the tibia is scaling with isometry or slight positive allometry. But exclusion of the extinct species means that the large living species of *Macropus* pull the regression line down to an overall negative allometric scaling: that is, that large extant kangaroos have tibia that are proportionally more slender in comparison with their length.

#### Summary of bivariate analyses

We interpret these different scaling relationships as follows: that it is not really the case that, compared with other kangaroos in general, that the larger extinct forms were “more robust”; rather, it is the extant large species of *Macropus* that are relatively gracile. To present this in a allegorical fashion: if the only medium-to-large sized felid that survived today was the cheetah, this would appear to be the “normal” form of a large cat, and the bones of an extinct leopard would be seen as being unexpectedly “robust”. But with the known diversity of extant felids, it is apparent that cheetah is a specialized gracile form, probably representing morphological adaptations for speed at a larger size. We propose that the same is true of the large extant kangaroos: they are the ones who do not follow the “normal” kangaroo scaling relationships – they are the ones who are exceptionally gracile while the sthenurines and *Protemnodon* spp. have the expected proportions. We propose that modern large species of *Macropus* are “cheetahs”: they do not represent the “norm” for kangaroos in general, and their gracility is probably an adaptation to allow them to maintain rapid hopping at a larger body size than optimal for this type of locomotion.

### Multivariate analyses

#### Principal Components Analysis of all hind limb bones (Figure 11A, Table 1)

**Figure 11:**
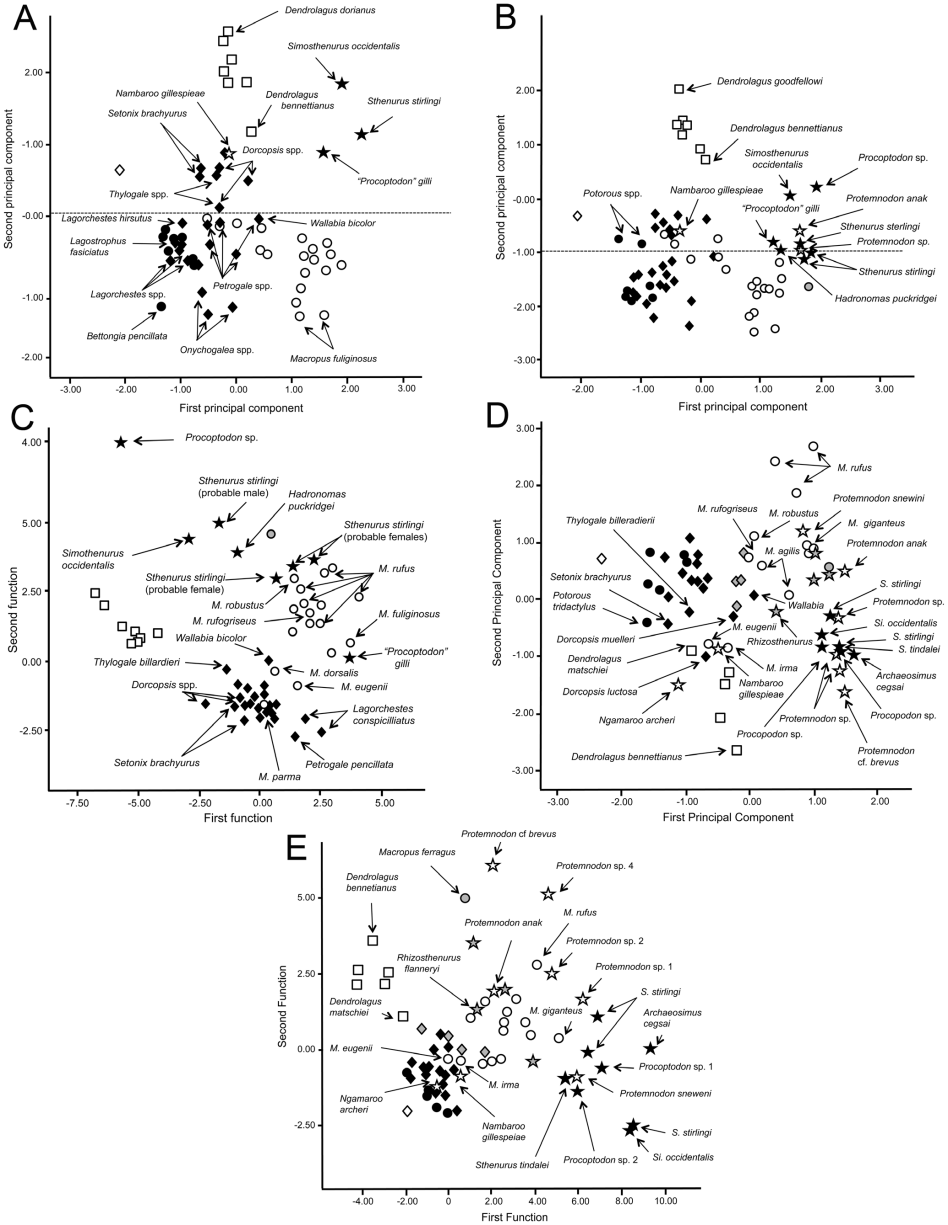
Multivariate analyses of hindlimb bones. Key as for Figure 10: taxa included and explanation of any composite fossils are listed and explained in Tables S3, 4. (A) Principal Components Analysis using all hind limb bones. The dotted line indicates the division between extant taxa that are more specialized hoppers, and those that are less specialized or that rarely hop. (B) Principal Components Analysis without the pelvis. The dotted line indicates the division between extant taxa that are more specialized hoppers, and those that are less specialized or that rarely hop. The placement of taxa along both components is very similar to that shown in Figure 11A, except where otherwise noted. (C). Discriminant Analysis using all hind limb bones. Sthenurines and extant macropodines (plus *Lagostrophus*) only. (D). Principal Components Analysis, calcaneum only. Key as for Figure 10 except for the following additions: half tone diamonds  =  extinct Miocene macropodine *Dorcopsoides*; half tone stars  =  Miocene sthenurines ( =  *Hadronomas puckridgi* unless otherwise indicated). (E). Discriminant Analysis, calcaneum only. Key as for (D).

The PCA performed from the dataset including all the bones yielded two eigenvectors with eigenvalues higher than 1.0, which jointly explained the 93% of the original variance. This analysis proved excellent for distinguishing between extant macropodoids that are more specialized hoppers from those that are rare, or less specialized hoppers (such as tree kangaroos, the non-hopping *Hypsiprymnodon moschatus*, and the forest and woodland dwelling wallabies). The first PC (λ = 79.263; 91.10% of variance explained) is interpreted as a size vector because all the variables had positive loadings and fairly high values (Table 2). However, the second PC (λ = 1.543; 1.774% of variance explained) is interpreted as a shape vector because not all the loadings of the variables on this eigenvector were positive (Table 1). The morphospace depicted from the scores of the specimens on these two PCs is shown in Figure 11A and the factor loadings of the variables on each eigenvector are shown in Table 1.

**Table 1:** Factor loadings for Principal Components Analysis: all hind limb bones.

Loading Positively on the second component			Loading negatively on the second component		
Var.	Variable Description	Load.	Var.	Variable Description	Load.
P13	Maximum width of tuber coxa	0.300	M1	Length of fourth metatarsal	−0.379
P15	Length of epipubic bone	0.296	Ph1l	Length of first phalanx on digit 4	−0.356
P12	Minimum width of tuba coxa	0.255	P17	Height of iliopectineal process	−0.316
P19	AP width of ischial tuberosity	0.236	P6	Length of puboischiatic symp.	−0.337
Ph3L	Length of third phalanx on digit 4	0.203	T6	Height of tibial crest	−0.318
C5	ML (mid width) of calcaneal tuber	0.205	T1	Length of tibia	−0.249
A4	Width of fibula facet on lat. ridge	0.199	P20	AP width of obturator foramen	0.198
F8	AP width of femoral head	0.140	Ph2L	Length of second phalanx on digit 4	−0.188
C7	DP (top width) of calcaneal tuber	0.122	P7	Dorsal length of ischium	−0.181

Key: AP  =  anteroposterior; DP  =  dorsoplantar; ML  =  mediolateral.

**Table 2:** Factor loadings for Principal Components Analysis: without the pelvis.

Loading Positively			Loading negatively		
Var.	Variable Description	Load.	Var.	Variable Description	Load.
Ph3L	Length of third phalanx	0.226	M1	Length of the fourth metatarsal	−0.336
C5	ML (mid width) of calcaneal tuber	0.183	Ph1L	Length of first phalanx	−0.320
F8	AP width of femoral head	0.112	T6	Height of tibial crest	−0.222
P19	AP width of ischial tuberosity	0.236	T1	Length of tibia	−0.212
A4	Width of fibula facet on lat. ridge	0.165	Ph2L	Length of second phalanx	−0.178
A8	ML width of medial tibial facet	0.108	T7	Length of proximal art. surface	−0.117
A2	Max. MW width of astragalus	0.107	C2	Lateral AP length of calcaneum	−0.102
C7	DP (top width) of calcaneal tuber	0.122	C3	Plantar AP length of calcaneum	−0.092
F11	Length of gluteal insertion	0.100	C1	Medial AP length of calcaneum	−0.088

Key: AP  =  anteroposterior; DP  =  dorsoplantar; ML  =  mediolateral.

The most obvious feature of Figure 11A is that, among the extant macropodoids, there is a division along the second component between specialized hoppers (with negative scores) and forms that either do not hop (the musky rat-kangaroo *Hypsiprymnodon moschatus*), hop rarely (the tree-kangaroos, *Dendrolagus* spp.), or which are unspecialized, forest-dwelling hoppers (the New Guinea forest-wallabies, *Dorcopis* spp. and *Dorcopsulus* spp., the quokka, *Setonix brachyurus*, and the pademelons, *Thylogale* spp.) The Oligo-Miocene balbarid *Nambaroo gillespieae* also falls within this clustering, supporting the hypothesis that these animals were not hoppers, or poor hoppers at best [47]. The tree-kangaroos form a distinct cluster with higher positive scores than any other macropodines: the one with the obviously lower scores, *Dendrolagus bennettianus*, is one of the more basal, Australian, members of this tribe (see [45]).

The macropodoids that have negative scores on the second component include the more specialized hoppers that are also more open-habitat forms: these include the swamp wallaby (*Wallabia bicolor*), the rock-wallabies (*Petrogale* spp.) the hare-wallabies (*Lagorchestes* spp. and *Lagostrophus fasciatus*), the nail-tail wallabies (*Onychogalea* spp.), and the species of the genus *Macropus* (i.e., “regular” kangaroos and wallabies). The species of *Macropus* on Figure 11A can be identified as follows: small species (*M. parma, M. eugenii*, and *M. dorsalis*), which cluster amongst the other terrestrial macropodines; medium-sized species (*M. rufogriseus*, *M. agilis*, and *M. parryi*), which have slightly more positive scores on the first component than any of the other macropodines; and large species with the highest scores on the first component (*M. antilopinus, M. fuliginosus, M. giganteus, M. robustus*, and *M. rufus*). Note how the scores of *Macropus* species on the second component change with increasing size, with the smaller species having the least negative scores, and the larger ones the most negative scores. This is not merely an allometric issue as the species of nail-tail wallabies (*Onychogalea* spp.), which are of a similar size to the small species of *Macropus*, and which have evolved more cursorial anatomy convergently with *Macropus* (see previous discusson), have similarly negative scores on the second component as the large species of *Macropus*. The only specimen which appears to be “out of place” here is the brush-tailed bettong or woylie, *Bettongia penicillata*: this potoroine is not notably cursorial in its anatomy or behavior, but may be falling with low scores on the second component because of relatively small epipubic bones (see later discussion): note that, when the analysis is performed without the pubis (Figure 11B) *B. penicillata* clusters with the other potoroines.

The three included sthenurine species have, unsurprisingly, high scores on the first component, due to their large size. But what is of great interest is that they also have high scores on the second component, falling with the extant macropodoids that rarely or never hop. (In fact, their negative scores put them within the morphospace of the tree-kangaroos.)

Table 1 shows the variables that are responsible for the distribution of the taxa along the second principal component. The pelvis features prominently in the placement of the taxa with negative scores: of prime importance are the width of the tuber coxa (the dorsal tip of the ilium, indicative of a large origin of the superficial gluteals and the cranial head of the caudofemoralis) and the length of the epipubic bone. Also important are the size of the ischial tuberosity (indicative of a large area for the origin of the hamstrings), a large femoral head, a broad calcaneal tuber, and a deep fibula facet on the astragalus. The only length measurement with high loadings on the second component is the length of the third phalanx of the fourth pedal digit: this seems to be reflecting the long claws on the hind limbs of the tree-kangaroos.

In contrast, length measurements figure prominently in the negative loadings on the second component: most important is the length of the fourth metatarsal and the first phalanx, with the length of the tibia and second phalanx being less important. The height of the iliopectineal process on the pelvis is an important element on this axis of the second component, and this can be seen especially in large species of *Macropus*. This serves as the area of origin of the pectineus, a short muscle that would act to rapidly move the leg forwards from a backwardly positioned femur, and may be important for rapid recycling of the limb during hopping. Three other variables (length of puboischiatic symphysis, anterior-posterior width of the obturator foramen, and the dorsal length of the ischium) relate to an elongated posterior portion of the ischium, which provides for a longer moment arm for the hamstring and adductor muscle complexes, important in powerful limb retraction during hopping (see previous discussion). Another variable with negative loadings includes the size of the tibial crest, reflecting a large area for the origin of the tibialis anterior, possibly reflecting rapid foot flexion (see previous discussion).

The difference between the positive and negative loadings on the second component is that the variables loading positively largely reflect adaptations for stability and power (large joint surfaces, large superficial gluteal muscles, broad tarsal bones, strong abdominal muscle support [epipubics]); while those loading negatively reflect adaptations for speed (long limb segment lengths, modifications for rapid and powerful limb retraction and protraction). Note that the position of the sthenurines on component two is despite their being unlike the other taxa in this position in many respects, in that they have variables with high negative loadings on this component, such as a long tibia, and a relatively long fourth metatarsal and first phalanx.

#### Principal Components Analysis without the pelvis (Figure 11B, Table 2)

As the pelvis is rarely completely preserved in fossil forms, we performed a second PCA, excluding those variables measured on the pelvis, in order to include more extinct taxa. Again, while the first PC (λ = 60.734; 93.443% of variance explained) was interpreted as a size vector according to the loadings of the variables on this axis (see Table 3), the second PC was interpreted as a shape vector (λ = 0.672; 1.034% of variance explained). Despite the fact that only the first PC had eigenvalues higher than one, we also extracted the second PC because it represents aspects of morphological differences among the specimens. The morphospace depicted from the scores of the specimens on these two PCs is shown in Figure 11B, and the factor loadings of the variables on each eigenvector are shown in Table 2.

**Table 3:** Coefficient loadings for Discriminant Analysis: all hind limb bones (sthenurines and extant macropodines only).

Loading on Factor 1			Loading on Factor 2		
Var.	Variable Description	Load.	Var.	Variable Description	Load.
F20	Length of medial tibial condyle	0.231	A2	ML width of astragalus	0.399
F12	Max width of greater trochanter ridge	0.212	F12	Max width of greater trochanter ridge	0.187
M1	Length of fourth metatarsal	0.085	M1	Length of fourth metatarsal	−0.005
A2	ML width of astragalus	−0.723	F20	Length of medial tibial condyle	−0.194

Key: ML  =  mediolateral.

Despite the fact that the pelvis figured so prominently in the first analysis, with its exclusion the taxa fall in a similar position within the morphospace. One notable difference is the more negative position along component two of the potoroos (*Potorous* spp.), which are the least specialized hoppers among the potoroines. However, regarding the scores of the different taxa, only *Dendrolagus* spp. have positive scores. Thus this analysis, more so than the first one, is distinguishing between tree-kangaroos and other forms. The position of the sthenurines on the second component, in a similar position to the less-specialized extant forms, largely reflects low values for the variables that have high negative loadings on this component. The sthenurines all have less negative scores on the second component than the large species of *Macropus*, including the extinct “giant” species *M. titan*. The Miocene sthenurine *Hadronomas puckridgi* falls relatively close to *Macropus*. Two sthenurines, unlike any extant form apart from *Dendrolagus* spp., have slightly positive scores on the second component: the largest, most specialized taxa, *Procoptodon* sp. (probably  =  *P. goliath*) and the somewhat smaller *Simosthenurus occidentalis*. Note that *Procoptodon* sp. and *S. occidentalis* tend to cluster together in all other analyses where both are included. A couple of *Protemnodon* species are included in this analysis, and they fall within the range of the less specialized sthenurines, likely indicating less specialized hopping abilities rather than any particularly sthenurine qualities.

Table 2 shows the variables that are responsible for the distribution of taxa along the second component. The most positively loading variable is the length of the third phalanx of the fourth digit, reflecting the long claws of the tree-kangaroos. Additional variables loading positively include a number of measurements of the astragalus that indicate a broader joint surface with greater rotational abilities of the ankle. Variables which would seem to apply to the sthenurines as well as *Dendrolagus* spp. include a larger femoral head, a broader and wider calcaneal tuber, and the length of the gluteal insertion on the femur, reflecting the large size of this muscle (which provides stability over the hip joint) previously indicated by the large tuber coxae.

New variables with negative loadings include a longer (in the anterior-posterior direction) proximal articular surface of the tibia (which reflects a longer tibial tuberosity, incorporated into the value of a higher tibial crest on the tibia), and several measurements of the length of the calcaneal tuber (reflecting the moment arm for the gastrocnemius muscle, the primary foot extensor). The most important of the calcaneal tuber length measurements is the lateral length, which reflects a greater amount of “stepping” of the articulation of the calcaneus with the cubonavicular (see previous discussion). Thus the negative loadings on this axis represent modifications for rapid hopping, as with the previous analysis.

#### Summary of Principal Components Analyses

The PCA of all the bones together proved excellent for distinguishing between extant macropodoids that are more specialized hoppers from those that are rare, or less specialized hoppers (such as tree-kangaroos, the non-hopping *Hypsiprymnodon moschatus*, and the forest and woodland dwelling wallabies), and the loadings of the variables along the second component made sense in terms of the functional morphology. In the analysis with all hind limb bones the Pleistocene sthenurines clustered with the rare hoppers, although the smallest form, *“Procoptodon” gilli*, had the lowest scores. With the pelvis removed, the distinction was not so marked and the analysis appeared to be mainly separating the tree-kangaroos (*Dendrolagus* spp.) from the other forms. The position of the sthenurines was not so clearly separate from the more specialized macropodines in this analysis, but all sthenurine individuals had scores that were more towards the positive end of the component than did any of the large species of *Macropus*.

#### Discriminant Analysis of all hind limb bones (Figure 11C, Table 3)

We originally performed this analysis with the complete range of taxa, but the results appeared to be somewhat skewed by the inclusion of the potoroines. While on the first function the two larger sthenurine species (*Sthenurus sterling* and *Simosthenurus occidentalis*) were clearly separate from all other macropodoids (apart from *Dendrolagus* spp.), the second function appeared to be distinguishing potoroines from other macropodoids, with the extinct balbarid *Nambaroo gillespeiae* being an extreme outlier at the opposite end of this function from the potoroines. This analysis is presented in the Supplementary Information (Figure S5, Tables A and B in Text S2): here we chose to do an analysis comparing only sthenurines with macropodines. No pelvis measurements were selected by the analysis with all the variables, so this analysis was not repeated with the exclusion of the pelvis. The morphospace depicted from the scores of the specimens on the two functions is shown in Figure 11C, and the factor loadings of the variables on each eigenvector are shown in Table 3.

Three groups were defined: Group 1 =  species of *Macropus*; Group 2 =  other extant macropodine species (plus *Lagostrophus*), excluding *Dendrolagus* spp.; Group 3 =  *Dendrolagus* spp. The extinct taxa were added as unknowns. The value of the Wilks' lambda statistic for the first function was close to zero and highly significant (λ = 0.04; χ2 = 137.221; d.f. = 8; P<0.001). Similarly, the value of the Wilks' lambda statistic for the second function was also highly significant (λ = 0.299; χ2 = 51.317; d.f. = 3; P<0.001). We obtained a 94.2% of correct reclassifications using the leave-one-out method of cross-validation, which suggests that both discriminant functions combined a set of skeletal traits that accurately distinguished the three groups compared.

The first function distinguishes *Dendrolagus* spp. (with negative values) from other extant macropodines. The more specialized sthenurines (*Procoptodon* sp. and *Simosthenurus occidentalis*), and the presumed male specimen of *Sthenurus stirlingi* (see [12]) also have high negative scores on function one, but only *Procoptodon* sp. falls into the same level of scores as *Dendrolagus* spp. The presumed female specimens of *S. stirlingi* (see [12]) have somewhat negative scores in comparison with the *Macropus* individuals, but “*Procoptodon*” *gilli* has the most positive of scores of any macropodid. The non-*Macropus* macropodines (excluding *Dendrolagus* spp.) tend to have more negative scores than the *Macropus* individuals, possibly reflecting less specialization for hopping (the non-*Macropus* individuals that have positive scores on this function tend to be the more hopping specialists, but there is no distinct separation within the group).

The only variable with negative loadings is the mediolateral width of the base of the astragalus. As notec previously, this feature may be distinguishing *Dendrolagus* spp. based on their more mobile tarsal joint, and *Procoptodon* sp. may be falling into this area of the morphospace because of its large size (and hence a proportionally larger ankle joint). Two measures of the femur have high positive scores on this axis: the maximum width of the greater trochanteric ridge, and the length of the medial tibial condyle. The width of the trochanteric ridge reflects the insertion of the gluteals, perhaps indicating larger hind limb retractor musculature in the species of *Macropus*. The functional significance of the length of the medial tibial condyle is not clear: as all the macropodids have a medial condyle that is shorter than the lateral one, this variable may be reflecting a more symmetrical distal femur, possibly related to hopping behavior. The length of the fourth metatarsal also has slight positive loadings, probably reflecting the difference from the very short metatarsals of *Dendrolagus* spp.

The second function mostly separates the sthenurines from the macropodines, with the exception of “*Procoptodon*” *gilli*. Perhaps surprisingly, *Hadronomous puckridgi* has higher scores on both this function, and the first one, than “*P*.” *gilli* and the presumed female individuals of *Sthenurus stirlingi*. The only macropodine to cluster among the high-scoring sthenurines is the exinct “giant” *Macropus titan*, possibly reflecting the fact that this function is largely (but not entirely) a reflecting body size. Note that the smaller species of *Macropus* cluster with the other macropodines, but there is no simple size-sorting among the larger species of *Macropus*, and *Hadronomas puckridgi* is no larger than the large extant species of *Macropus*. And, of course, *Dendrolagus* spp., with higher scores than any of the non-*Macropus* species of macropodines, are not any larger than all of these taxa.

With regard to the variables with high loadings on the second function: here the mediolateral width of the astragalus has the highest positive loadings; this may be the variable that is acting to sort individuals by body size, in part (and the relatively high scores of *Dendrolagus* spp. are explained by their more flexible ankle). The maximum width of the greater trochanteric ridge also has relatively high scores: all of the *Macropus* species have positive scores on this function, as they did with this variable on the first function. However, the sthenurines also have high scores: as previously discussed, sthenurines have large areas for gluteal origin and insertion, but here perhaps reflecting balance over an upright trunk rather than limb retraction. The length of the fourth metatarsal here has weakly negative scores, again possibly explained by the position in the morphospace of *Dendrolagus* spp. However, the length of the medial tibial condyle has the highest negative scores. This may reflect the relatively larger length of the lateral tibial condyle observed in sthenurines, as previously discussed.

To investigate this further we performed a CVA with and without *Dendrolagus* spp. (not illustrated): this resulted in just two predetermined groups (species of *Macropus* and other macropodines [plus *Lagostrophus*]), with the sthenurines classified as unknowns. The value of the Wilks' lambda statistic for the first function was highly significant (λ = 0.266; χ2 = 49.019; d.f. = 2; P<0.001) and the 89.8% of the taxa were correctly classified. The function incorporated only two variables, both loading positively. These were the height of the medial malleolus on the distal tibia (T14: highest loading) and the width of the greater trochanteric ridge (F12). On a univariate axis non-*Macropus* macropodines have the lowest scores, species of *Macropus* have intermediate scores, and the sthenurine species have the highest scores. These features relate to the relative stability of the ankle joint and to the relative size of the gluteals. Thus it seems that the *Macropus* species have a more stable ankle joint and larger gluteals than non-*Macropus* macropodines, perhaps due to greater hopping specializations, while the sthenurines are more extreme in these features, but perhaps for reasons unrelated to hopping. The larger gluteals in sthenurines could reflect the balancing of the trunk over the hips while foraging, and the more stable ankle joint could reflect balancing the body weight over one leg while striding. The fact that the width of the astragalus, which featured prominently in some earlier analyses, is not included here confirms the suspicion that the high loadings of this variable in other analyses largely distinguished *Dendrolagus* spp., with their more flexible ankles, from other macropodids.

#### Principal Components Analysis of the calcaneum (Figure 11D, Table 4)

The analysis of the calcaneum yielded a first principal component (λ = 22.652; 94.38% of variance explained), which represents body size, and a second component (λ = 0.301; 1.254% of variance explained), which represents aspects of morphological differences among the specimens. Despite the fact that only the first PC had eigenvalues higher than one, which means that body size is responsible for a high amount of the total shape variation, we also extracted the second PC because it represents aspects of morphological differences among the specimens. The morphospace depicted from the scores of the specimens on these two PCs is shown in Figure 11D and the factor loadings of the variables on each eigenvector are shown in Table 4.

**Table 4:** Factor loadings for Principal Components Analysis: Calcaneum only.

Loading positively on the second component			Loading negatively on the second component		
Var.	Variable Description	Load.	Var.	Variable Description	Load.
C17	Width of sulcus for tendon of peroneus longus	0.284	C5	ML width of midshaft of tuber (dorsal ridge only)	−0.187
C25	Length of roughened area on plantar side of tuber	0.138	C18	Width of sulcus for tendon of flexor digitorum longus	−0.174
C3	Medial AP length	0.095	C16	ML width of midshaft of tuber (plantar side)	−0.165
C12	AP length of tuber	0.094	C15	Length of ectal facet	−0.154
C2	Lateral AP length	0.091	C6	DP width of top of tuber	−0.078
C11	AP length of CLAJ	0.090	C13	Mediolateral width across CLAJ	−0.077
C1	Plantar DV length	0.088	C14	Maximum width of calcaneal head	−0.073

Key: AP  =  anteroposterior; DP  =  dorsoplantar; ML  =  mediolateral. CLAJ  =  continuous lower ankle joint.

Taxa that have positive values on the second component (the first component representing body size) comprise most of the extant macropodoids, with the exception of the tree-kangaroos (*Dendrolagus* spp.) Perhaps surprisingly, the basal species of *Dendrolagus*, *D. bennettianus*, is the one here with the highest negative loadings, falling away from the other kangaroos, whereas in the PCA for all hind limb bones it was the one *Dendrolagus* species that tended to cluster with the other kangaroos. The extant macropodoids that place with negative scores include mainly the non-specialized hoppers: *Potorous* spp. (potoroos), *Setonix brachyurus* (the quokka), *Thylogale* spp. (pademelons), and *Dorcopsis* spp. (New Guinea forest-wallabies), but not *Hypsiprymnodon moschatus*. The Oligo-Miocene taxa (the macropodid *Ngamaro archeri* and the balbarid *Nambaroo gillespieae*) also have negative scores, but most of specimens of the late Miocene macropodine, *Dorcopsoides* sp., have positive scores. Perhaps surprisingly, the smallest species of *Macropus*, *M. eugenii* and *M. irma*, also have negative scores, as does the swamp wallaby, *Wallabia bicolor*.

Most of the large extinct taxa (sthenurines and *Protemnodon* spp.) have negative scores, with the largest forms (e.g., *Procoptodon* sp., *Protemnodon* cf. *brevus*) having the most negative ones. The smaller, and/or more gracile species of *Protemnodon*, *P. snewini* and *P. anak*, have positive scores, clustering with the larger species of *Macropus*, as do the several individuals of the Miocene sthenurine *Hadronomas puckridgi*. The smaller Miocene sthenurine *Rhizosthenurus flanneryi* has slightly negative scores, but still falls within the range of extant hopping macropodines. But basically, as with the PCA for all hind limb bones, the Pleistocene sthenurines and the larger species of *Protemnodon* fall with the rarely-hopping extant macropodines, in particular with the tree-kangaroos (*Dendrolagus* spp.).

The highest positive loading variable on the second component is the width of the sulcus on the latero-plantar side of the calcaneal head for the tendon of the peroneus longus muscle. As discussed previously, this action of this muscle it to both flex the ankle and to counteract the action of the tibialis anterior in preventing the evulsion of the pes. Thus this reflects morphology specialized for rapid hopping, and accounts for the high scores of the larger species of *Macropus* (especially *M. rufus*) on this component. Almost all of the other variables loading positively on the second component reflect the length of the calcaneal tuber: a long calcaneal tuber indicates a longer moment arm for the gastrocnemius muscle, again indicative of powerful and/or rapid hopping. Also loading weakly with positive values is the dorso-ventral length of the CLAJ (continuous lower ankle joint), which may simply reflect a rather narrow calcaneal head.

The variables loading with high negative loadings on the second component largely reflect the width of both the calcaneal tuber and the calcaneal head, especially the CLAJ. A broader calcaneum reflects foot stability rather than rapid locomotion. Also loading with high negative values is the width of the sulcus for the tendon of the flexor digitorum longus, on the medio-plantar side of the calcaneal head. This loading largely reflects the width of this sulcus in the tree-kangaroos, and may be related to their climbing ability. Another variable with moderately negative high loadings on the second component is the size of the ectal facet, which is one of the places where the astragalus articulates with the calcaneum (the other being the sustentacular facet). Thus a large ectal facet represents morphology adapted for weight-bearing and foot stability.

#### Discriminant Analysis of the calcaneum (Figure 11E, Table 5)

Three groups were defined: Group 1  =  non-hopping or occasionally hopping taxa (species of *Dendrolagus* plus *Hypsiprymnodon moschatus*); Group 2 =  regular hoppers (macropodines [including *Lagostrophus*] with the exception of the genera *Dendrolagus* and *Macropus*); Group 3 =  specialized hoppers (the species of *Macropus*). The extinct taxa were added as unknowns. The analysis yielded two functions that together allow a 93.2% of correct assignments using the leave-one-out method of cross-validation, which suggests that both discriminant functions combined a set of skeletal traits that accurately distinguishes among the three groups compared. The value of the Wilks' lambda statistic for the both functions is significant (Function I: λ = 0.092; χ2 = 94.067; d.f. = 8; P<0.001; Function II: λ = 0.465; χ2 = 30.213 d.f. = 3; P<0.001). The morphospace depicted from the scores of the specimens on both functions is visually displayed in Figure 11E and the factor loadings of the variables on each eigenvector are shown in Table 5.

**Table 5:** Coefficient loadings Discriminant Analysis: Calcaneum only.

Loading on Factor 1			Loading on Factor 2		
Var.	Variable Description	Load.	Var.	Variable Description	Load.
C21	Dorso-plantar width across surface of cubonavicular facets	0.933	C13	Medio-lateral width across CLAJ	0.586
C13	Medio-lateral width across CLAJ	0.014	C11	Dorso-ventral width across CLAJ	0.239
C11	Dorso-ventral width across CLAJ	−0.533	C5	Medio-lateral width of midshaft of tuber (main anterior ridge)	−0.111
C5	Medio-lateral width of midshaft of tuber (main anterior ridge)	−0.615	C21	Dorso-plantar width across surface of cubonavicular facets	−0641

Key: CLAJ  =  continuous lower ankle joint.

This analysis picked out four variables: two relating to the size and shape of the continuous lower ankle joint (where the astragalus contacts the calcaneum), one relating to the width of the calcaneal tuber, and one relating to the width of the cubonavicular facet at the base of the calcaneum, where the calcaneum contacts the cubonavicular bone.

For the first function the positive values largely reflect the dorso-plantar width of the cubonavicular facets (i.e., across the lateromedial and ventromedian facets). This function appears to reflect body size, in part, as among the extant kangaroos the larger forms (e.g., the larger species of *Macropus*) have more positive scores, and most of the Pleistocene sthenurines also have highly positive scores. However, this function is one of the few that distinguishes between the tree-kangaroos and the sthenurines. Tree-kangaroos have strong negative scores on this function, probably as the result of their merging of the ventromedian facet with the dorsolateral facet, which may allow for greater intratarsal motion [45], while the high positive scores of the sthenurines reflect the lengthening of the ventromedian facet. The variable loading with the greatest negative values on function one is the mediolateral width of the calcaneal tuber, a variable that is also large in sthenurines, despite the fact that they cluster with positive scores on this function: this variable also distinguishes *Dendrolagus* spp. from the other macropodoids. The dorsoventral width of the CLAJ also has high negative values on function one, reflecting a relatively narrow articulation between astragalus and calcaneum, again a feature of the smaller macropodines and *Dendrolagus* spp.

The variable with the highest positive loadings on the second function is the mediolateral width across the CLAJ: the tree-kangaroos have high positive scores on this function, and this may reflect an ankle joint that has some mediolateral mobility, as employed in climbing. The dorsoventral width of the CLAJ also has high positive values on this function: the larger species of *Macropus* and *Protemnodon* have positive scores on the second function (see discussion below), while most of the other extant macropodoids have negative (or at least less positive) scores. A narrow CLAJ may reflect a tarsal morphology that is better adapted for a restricted range of motion of the leg about the foot in the parasaggital plane, at least in the smaller taxa. The widths of the calcaneal tuber and the cubonavicular facet have negative loadings on the second function, reflecting the low scores of the larger sthenurines. The smaller and/or more gracile Miocene sthenurines, *Rhizosthenurus* and *Hadronomas*, tend to cluster with the extant kangaroos on both functions.

An interesting observation is that, while function one seems to represent a size axis in part, with the large sthenurines having the highest scores, other “giant” kangaroos do not cluster in this morphospace: rather, the large species of *Protemnodon*, and especially the extinct *Macropus ferragus*, have relatively low values on function one, but high values on function two. This may represent independent evolution of large size and mode of weight bearing over the foot: while the sthenurines enlarge the ventromedian cubonavicular facet, other large kangaroos have a larger overall size of the CLAJ.

#### Summary of the multivariate analyses of the calcaneum

The results of the PCA were similar to that of the other PCAs: the Pleistocene sthenurines were distributed along the second component in a similar fashion to *Dendrolagus* spp. and the less specialized terrestrial macropodines. The Miocene sthenurine *Hadronomas puckridgi* clustered with the larger species of *Macropus*, while the smaller Miocene sthenurine *Rhizosthenurus flanneryi* occupied a fairly middling position in the morphospace. The CVA was the only multivariate analysis to separate the sthenurines from the tree-kangaroos. Here the Pleistocene sthenurines occupied more or less their own area of the morphospace along the first function: the distribution of other large-bodied taxa showed that this was not simply an effect of their large size.

## Discussion

### Overview

The descriptive anatomy of Pleistocene sthenurines shows that they have numerous differences from large macropodines, over and above their greater “robusticity”. The bivariate and multivariate analyses show that they tend to cluster away from the macropodines in the morphospace. The larger species (*Sthenurus stirlingi*, *Simosthenurus occidentalis*, and *Procoptodon* sp.) invariably occupy a different portion of the morphospace to the macropodines (apart from *Dendrolagus* spp., [tree-kangaroos] with which they tend to group); and while the smaller “*Procoptodon*” *gilli* is usually distinct from the macropodines in the bivariate analyses (see especially Figures S1A, S1C, S2B, and S3B) it sometimes clusters with them in the multivariate analyses. In the analyses where both are included, *Procoptodon* sp. and *Simosthenurus* tend to group together, away from the species of *Sthenurus* (see Figures 11B, C, S5B), despite the size difference (*Simosthenurus* is considerably smaller than both *Procoptodon* and *Sthenurus stirlingi*). However, both *Procoptodon* and *Simosthenurus* are more specialized in their skull and dentition than *Sthenurus* [6], and they may also be more specialized in their postcranial anatomy. The Miocene wallaby-sized sthenurine, *Rhizosthenurus flanneryi*, represented here only by the pes, is not distinguished from similar-sized macropodines. The larger, grey kangaroo-sized Miocene sthenurine *Hadronomus puckridgi*, represented here by all elements except the pelvis, sometimes clusters with the macropodines (e.g., Figures 11B, D, and most of the SI bivariate plots), and sometimes with the larger Pleistocene sthenurines (e.g., Figure 11C).

The inclusion of other extinct taxa (*Nambaroo gillespieae*, *Ngamaroo archeri*, and *Dorcopsoides* sp.) in many of the analyses shows that they generally cluster with the extant macropodoids, supporting the hypothesis that it is the sthenurine anatomy that is distinctive from the general macropodoid bauplan (rather than representing the primtivie condition). The inclusion of the species of *Protemnodon* is interesting: the smaller (*P. snewini*) or more gracile (*P. anak*) forms often cluster with the similarly sized macropodines, especially on the bivariate plots. However, larger species tend to cluster with the sthenurines (e.g., Figures 11B, C) or occupy a different portion of the morphospace from either sthenurines or extant macropodines (e.g., Figures 11E, S5B). Although the locomotion of *Protemnodon* spp. is not a subject of this paper (they were included primarily because they represent a different lineage of large, robust kangaroos), these results may indicate that the larger species, at least, were not hopping (or not deploying hopping as their habitual gait), but did not have a similar type of locomotion to the sthenurines.

The large extinct species of *Macropus* (*M. titan* and *M*. *ferragus*) also behave in an interesting fashion in the analyses. In terms of the tibia diameter, at least, they are as “robust” as the other large extinct kangaroos (see Figure 9B). In the other bivariate plots (Figures S1–S3) they tend to follow the trajectory of the extant species, except in some features of the ankle joint (Figures S3B, C) where they cluster more with the other large extinct forms. In the multivariate analyses they sometimes group with the other species of *Macropus* (Figures 11B, D, S5B), with the larger sthenurines (Figure 11C), or in a different portion of the morphospace to either (Figure 11E). In any event, their inclusion shows that the difference between the large sthenurines and the smaller-sized extant large species of *Macropus* is not simply a matter of body size.

However, while the results here clearly indicate that at least the larger sthenurines were distinctly different in their hind limb anatomy from the large extant macropodines, they can at best support the hypothesis that sthenurines were different in their locomotion from extant kangaroos: they cannot provide evidence of a specific other type of locomotion, as there are no similar extant forms with which they could cluster. Using the results, and the implications of the differences in the descriptive anatomy, we present an argument based on principles of functional anatomy.

We summarize the aspects of sthenurine anatomy that differ from those of large hopping macropodines as follows: *(i)* anatomy of sthenurines that indicates they would have had difficulty with *Macropus*-like rapid hopping; *(ii)* anatomy indicative of a habitual more upright posture, with the femur at an angle more parallel to the iliac blade and with less flexed limbs; and *(iii)* anatomy indicative of bearing weight on one foot at a time, as would be the case with a locomotor gait of bipedal walking. While modifications listed under *(ii)* could be taken as indicative merely of bipedal browsing with an upright trunk, in combination with *(i)* and *(iii)* they also lend support to this posture being employed during locomotion. Important differences in anatomy are presented in Tables 6, 7.

**Table 6:** Summary of important differences between sthenurines and large species of *Macropus* 1: Lumbar vertebrae, pelvis and proximal limb bones.

Bony element	Morphology in large macropodines	Morphology in sthenurines	Implications of derived morphology	Functional relevance
Lumbar vertebrae (general)	*Unmodified*	Enlarged, massive	Resistance to rotational torsion	BS
Transverse processes	*Unmodified*	Reduced or absent	Reduction in back flexibility (reduced longissimus dorsi muscles)	FP and/or BS
Metapophyses	*Unmodified*	Enlarged and laterally expanded	Reduction in back flexibility (enlarged multifidus muscles)	FP or BS
Sacrum	*Unmodified, two vertebrae*	Enlarged, may include three vertebrae.	Resistance to rotational torsion	BS
Iliac blade	*Unflared, narrow tuber coxae*	Flared laterally, broad, enlarged tuber coxae@	Greater origin of gluteal & iliacus muscles	FP and/or BS
Width between acetabulae	*Relatively narrow*	Relatively broad	More stable bipedal stance	FP
Ischium length	Elongated#	*Not elongated*	Increased moment arm of hamstring muscles	RH
Angle between ischium and ilium	170^0^	145^0^ (i.e., tipped dorsally)@	Reorientated moment arm of hamstring muscles	FP and/or BS
Epipubic bones	Relatively short#	Long and broad@	Stiffening of trunk, resist rotational torsion	FP and/or BS
Femoral head size	*Regular*	Enlarged (i.e., larger hips)@	Increased weight-bearing	BS
Femoral head shape	Ovoid	*Round*	Restricts movement to parasaggital plane	RH
Femoral neck	Elongated	*Regular*	Increased moment arm of gluteals	RH
Lesser trochanter of femur	*Regular position*	Placed distallybroad@	Increased moment arm of iliopsoas	BS and/or FP
Position of femoral adductor scar	*Regular position*	Placed distallydistallybroad@	Increased moment arm of m. quadratus femoris	BS and/or FP
Femoral condyle width	*Unmodified*	Increased (i.e., larger knees)@	Increased weight-bearing	BS
Tibial tuberosity	Long#	*Short*	Increased area of insertion of tibialis anterior	RH
Medial malleolus of tibia	*Unmodified*	Longer and more robust	Stabilization of tibia-astragalar articulation	BS and/or FP
Tibial distal plantar flange	*Absent*	Prominent	Stabilization of tibia-astragalar articulation	BS and/or FP

Key: Italics indicate the primitive condition for the morphology. BS  =  bipedal striding. FP  =  foraging posture. RH  =  rapid hopping. Italics indicate the primitive condition for the morphology (as determined by the condition in generalized small macropodines such as *Dorcopsis*).

^@^  =  also seen in tree-kangaroos (Dendrolagus spp.).

# =  also seen in smaller, rapid-hopping macropodines (e.g., species of *Onychogalea* and *Lagorchestes*).

**Table 7:** Summary of important differences between sthenurines and large large species of *Macropus* II: Pes.

Bony element	Morphology in large macropodines	Morphology in sthenurines	Implications of derived morphology	Functional relevance
Astragalar trochlear groove	*Shallow with low ridges*	Deep with high ridges, esp. medial	Stabilization of tibia-astragalar articulation	BS
Astragalar fibular facet	*Narrow*	Broad	Increased weight-bearing	BS
Medial malleolar astragalar process	*Broad*	Narrow	Reduced intratarsal mobility	BS and/or FP
Calcaneal tuber	Long, posteriorly reflected#	*Shorter, straight or anteriorly reflected*	Increased moment arm of m. gastrocnemius	RH
Calcaneal tuber dorsoventral width	*Unmodified*	Deep	Increased weight-bearing	BS
Calcaneal tuber tip	*Unmodified*	Broadened in mediolateral plane@	Larger insertion area of Achilles tendon	BS and/or FP
Transverse plantar sulcus on calcaneum	Broad#	*Narrow*	Larger sulcus for peroneus longus tendon	RH
Fibular facet on calcaneum	*More medial position*	More lateral position	Greater ability to pronate foot	BS and/or FP
Sustentaculum tali on calcaneum	*Broad medio-laterally*	Narrow medio-laterally	Greater ability for plantar flexion with internally rotated foot	BS and/or FP
Cubonavicular facet on calcaneum	Pronounced “stepping” of dorsolateral facet	*Moderate “stepping” of dorsolateral facet*	Restriction of motion between calcaneum and cuboid	RH
Cubonavicular facet on calcaneum	*Relatively narrow*	Elongated in dorsoplantar direction	Increased weight-bearing	BS
Length of fourth metatarsal	Greatly elongated	*Moderately elongated*	Increased length of distal limb	RH
Proximal articular surface of fourth metatarsal	*Flat anterior profile, small plantar eminence*	Enlarged & curved anterior profile, large plantar eminence	Increased weight-bearing	BS
Distal articular surface of fourth metatarsal	*Square-shaped*	Larger and medio-laterally broadened.	Increased weight-bearing	BS
Metatarsal keels	*Moderately prominent*	Less prominent	Stabilization of phalanges on metatarsus	RH
Proximal phalanx of fourth pedal digit	Elongated#	*Regular length*	Lengthening of plantar tendons, more “springy” foot.	RH
Middle phalanx of fourth pedal digit	*Regular length*	Elongated	Increased weight-bearing	BS

Key: Italics indicate the primitive condition for the morphology. BS  =  bipedal striding. FP  =  foraging posture. RH  =  rapid hopping. Italics indicate the primitive condition for the morphology (as determined by the condition in generalized small macropodines).

^@^  =  also seen in tree-kangaroos (Dendrolagus spp.).

# =  also seen in smaller, rapid-hopping macropodines (e.g., species of *Onychogalea* and *Lagorchestes*).

#### Anatomical features indicative of limited hopping at best

The anatomy of the sthenurine lumbar spine is contraindicative to hopping. Hopping in extant kangaroos entails a considerable amount of flexion of the lumbar vertebral column, especially at the point of the anticlinal ( =  diaphragmatic) vertebra [61], but the morphology of the sthenurine backbone appears to be specifically designed to limit mobility in this area. Note also virtual loss of the vertebral transverse processes, indicating great reduction of the longissimus dorsi musculature, which is essential for creating dorsiflexion of the spine, and counterbalancing regular flexion. Wells and Tedford [12] interpret sthenurine lumbar anatomy as rigidity to resist rotational stress on the backbone, which they note can be caused by alternate limb loadings in mammals: but why would sthenurines require a backbone resistant to rotational stress if they were moving both hind limbs simultaneously in a kangaroo-like fashion? The anatomy of the lumbar vertebrae in sthenurines indicates an extremely rigid back, where in addition the muscles that flex the spine have been greatly reduced. This would limit the ability to hop, but would brace the backbone against the rotational forces that would be generated by alternate limb loadings. The large epipubic bones also indicate a bracing of the trunk.

The reduction of the sthenurine tail is also a problematical issue. Macropodine kangaroos can use their tail as a support when standing on their hind legs, both when standing plantigrade in repose, or standing digitigrade to fight [1]. As this digitigrade standing is the posture proposed for sthenurines browsing, it seems strange that they would have *reduced* the size of their tail. In addition, the tail is important in hopping locomotion: the tail is swung downwards as the hind limbs swing backwards, thus cancelling the inertia of the hind limbs [25]. If sthenurines hopped in a *Macropus*-like fashion, then their heavier limbs would require an even more sturdy tail to balance out these inertial forces. However, if they were employing bipedal striding, then a large tail would no longer be necessary to cancel out inertial forces, and additionally a large tail could possibly impede such locomotion by dragging on the ground. The relatively shorter and less muscular tail of sthenurines may also reflect its lack of use in pentapedal locomotion.

Sthenurines also lack many of the features of *Macropus* that are specialized for hopping (although this would not rule out the ability to hop): these features in *Macropus* include the elongated ilium, the long tibial tuberosity and prominent tibial crest, the elongation of the trochlea of the astragalus, the long calcaneal tuber, the enlarged sulcus on the calcaneum for the peroneus longus muscle, and the elongated proximal phalanx on the fourth medal digit. Note also the hoof-like ungual phalanges in sthenurines, which Kear et al. [47] interpreted as an adaptation for “hopping at slower speeds”.

#### Anatomical features indicative of a habitual upright posture

That is, with a fairly upright trunk, the femur in a more vertical position, and the limb joints with more obtuse angles (i.e., less “crouched”).

The morphology of the sthenurine ischium, tipped dorsally and forming a more acute angle to the ilium, repositions the moment arm for the hamstring muscles, important in limb retraction. This ischial anatomy is seen in placentals among primates that locomote with an upright trunk, as it maintains the moment arm of the hamstrings when the femur is rotated posteriorly in this posture [62]. Convergence in this feature is seen in tree-kangaroos (and also koalas, see plate XIV in [35]), supporting the hypothesis that this anatomy is related to the trunk position relative to the femur. Tree-kangaroos and koalas also resemble sthenurines in having extremely large epipubic bones, which Elftman [35] interpreted as important for supporting the viscera with an upright trunk, due to the insertion of the hypaxials on the epipubics.

The enlarged tuber coxae of the ilium, also echoed to a certain extent in tree-kangaroos (see Figure S1A), reflect enlarged superficial gluteal muscles and an enlarged cranial head of the caudofemoralis [36]. These muscles would be important for raising the front end of the body up over the hips, as would be necessary in a bipedal feeding posture.

The more distal placement on the femur of the adductor scar and the lesser tuberosity would increase the moment arms of the quadratus femoris and iliopsoas, respectively. This might relate to more powerful limb adduction and protraction, but might simply reflect compensation for the altered moment arms of these muscles occurred by a difference in the position of the femur relative to the pelvis with an upright trunk.

The shorter calcaneal tuber in sthenurines might also reflect a change in posture at the ankle joint. In considering forces acting over any joint, the moment arm produced by the limb extensors must balance the ground reaction force. Crouched limb postures result in a long lever arm for the ground reaction force, and the postural changes in larger animals reduce this lever arm, increasing the limb's effective mechanical advantage [30]. As noted previously, extant kangaroos do not change their locomotor posture with increasing size. The long tuber in large species of *Macropus* reflects an increase in the moment arm for the limb extensors (the gastrocnemius, in this case), to balance out the large ground reaction forces occasioned by a crouched posture, which become relatively larger with larger body size. The shorter tuber in sthenurines implies a lesser moment arm for the gastrocnemius, which given their large size would only be biomechanically possible if the moment arm for the ground reaction force was also reduced, as would be occasioned by a more upright posture at the ankle joint. The very broad area on the calcaneum for the attachment of the Achilles tendon in sthenurines may indicate an insertion that is fleshy rather than tendinious; if this were the case, this would definitely be in contradiction to a hopping mode of locomotion.

#### Anatomical features indicative of bearing weight on one foot at a time

Part of the greater “robustness” of the hind limbs of sthenurines could be explained by each limb having to bear the full weight of the animal at some point in the locomotor cycle, something not experienced by hoppers: as noted earlier, the real issue here may be that large species of *Macropus* are unusually gracile, so that sthenurines appear to be “robust” only in comparison with these highly specialized hoppers. Many morphological features of sthenurine hind limbs actually parallel those seen in humans that distinguish them from other apes. In the human condition this obviously relates to walking bipedally rather than quadrupedally, rather than a transition from hopping, but the same principles apply.

The short and broad sacrum of sthenurines could be interpreted as resisting greater rotational forces, as occasioned by walking with alternate limbs (as could, as well, the more rigid lumbar spine, as discussed above). Humans have a broader sacrum than other apes, which is interpreted as relating to weight support and transmission during locomotion with a habitual bipedal posture [43]. In the sthenurine pelvis, the inflated tuber coxae indicate an enlargement of muscles (gluteus superficialis and caudofemoralis) that would not only elevate the body, but would also balance the body over a single leg and prevent medial tipping by their abduction action. This is reminiscent of the human morphology of the enlargement and repositioning of the gluteus superficialis (via the shorter and broader iliac blades), interpreted as an adaptation for bipedal walking in preventing collapse at the hip while balancing on one leg [43]. The enlarged gluteus medius and minimus, as well as the iliacus, as indicated by the expanded iliac blade in sthenurines, could also aid in this postural support. Enlarged areas of insertion for the gluteals are also seen in camelids, who balance the body over pairs of lateral legs during pacing locomotion, and who thus require large limb abductor muscles to prevent the body from collapsing medially [49].

The enlarged femoral head and distal condyles in sthenurines are reminiscent of the differences between the human and ape conditions [43], and may be indicative of increased load bearing in supporting the weight on a single leg. Elliptically shaped femoral condyles, as seen in sthenurines, are also a new feature in humans, interpreted as minimizing the load on the knee [43]. The elliptical shape in humans also increases the moment arm of the quadriceps femoris, which aids in maintaining balance in a straight-legged position [43]; this morphology in sthenurines could also reflect walking with a straighter knee.

Aspects of the sthenurine ankle joint have been interpreted as rotating the feet inwards for a more medial position, and/or for bearing more weight on the medial side of the foot: these include the rotation of the tibioastragalar joint in an anteriomedial direction; the longer groove in the proximal tibia for the insertion of the fibula, allowing for a greater internal rotation of the lower limb about the knee; the lateral displacement of the fibular facet as enabling greater ability to pronate the foot; a sustentaculum tali that is narrow in the mediolateral direction (allowing for plantar flexion with internal rotation of the foot); and the plantar crest of the calcaneum being more elongated on the medial side (see [12], [42]). Humans (as opposed to apes) have a suite of morphological adaptations related to the shifting of their weight to the medial side of the foot during locomotion [43]. These features of sthenurines may again be indicative of weight bearing on one leg at a time.

Another suite of features of the sthenurine ankle joint include ones relating to a greater stabilization of the tibia on the astragalus, and ones resisting movement of the astragalus on the calcaneum. Such features could indicate a need to resist greater rotational forces incurred by bearing weight on one leg at a time. Sthenurines are unique among macropodoids in having a plantar process on the distal tibia that fits in a tongue-in-groove linkage into the astragalar trochlea (also seen in the Miocene *Hadronomas*). An analogous morphology is also seen in cursorial placental mammals such as canids and horses, and is also present in the thylacine ( =  the “marsupial wolf” *Thylacinus cynocephalus*; personal observation of the senior author). In conjunction with this, the trochlear groove on the astragalus is deeper in sthenurines, with a raised medial trochlear ridge. There is also evidence of an increase in the size in the ligaments that bind the astragalus to the calcaneum [44], and the astragular trochlea has been rotated medially, now being at more of a right angle to the longtitudinal axis of the pes; this would restrict the movement of the tibia on the pes to a more anteroposterior motion, with compressive stresses being directed more anteroventrally (see [12]). The constriction of the sulcus for the flexor digitorum longus in sthenurines, intepreteted by Bishop [44] as preventing the dislodging of this tendon while elevating the foot, could also indicate greater stress on one foot at a time. A narrow medial malleolar process, and the larger and more posteriorly directed cuboid facet, would restrict any motion between the astragalus and the cuboid.

There are a number of morphological features indicative of overall greater weight bearing by both the tarsus and the foot. The lateral trochlear ridge on the astragalus, that forms an articulation with the fibula, is thicker in sthenurines than in other macropodoids. The overall size of the calcaneonavicular articulation is proportionally greater in sthenurines, in particular with a very deep ventromedian facet, rendering the shape of the joint square rather than rectangular. Correspondingly, the proximal articular surface on the fourth metatarsal is proportionally larger, and rendered more square in shape by an enlarger plantar eminence. The distal articular surface of the fourth metatarsal is also proportionally larger and more rectangular, matched by a similarly shaped proximal articulation on the proximal phalanx. Both proximal and distal articular surfaces are enlarged on the proximal and medial phalanges, giving these bones a “waisted” or “I-beam” shape.

Finally, there are a number of features indicative of enlarged plantar ligaments on the pes of sthenurines. These could be interpreted as being related to springiness of the foot during hopping (see e.g., [52]), but elastic energy storage in the hind limb in general is counter-indicated by the constriction of the sulcus for the flexor digitorum longus and the lesser mechanical advantage of the gastrocnemius (occasioned by the shorter calcaneal tuber). The larger plantar crest on the fourth metatarsal in sthenurines is indicative of larger interosseus muscles, and the well-developed scars for plantar cruciate ligaments on the plantar side of the proximal phalanx could as equally well indicate greater torque being placed on this bone [42], and stabilization of the foot under greater pressure while bearing weight on one leg at a time.

### A hypothesis about the sthenurine monodactyl condition

None of the scenarios of locomotor evolution in sthenurines account for the issue of the loss of the fifth pedal digit (the original idea of Tedford [52], that this represented a greater specialization for hopping, has now been abandoned; see earlier discussion). Although Murray [42] considers that the weight had already shifted to the medial side of the foot in the Miocene sthenurine *Hadronomas* (see also Kear [16], for *Rhizosthenurus*), thus rending the animal functionally monodactyl, this does not explain why the digit would become vestigial. An explanation is proposed here based on developmental trajectories. Pigs (*Sus scrofa*), like other artiodactyls, have digits three and four as the primary ones on both fore and hind feet, with the reduction of digits one, two and five. Developmental studies show that this has occurred through evolutionary modifications in the patterning of their limbs: developing limb buds of pigs are different from those of a mouse, with a reduction of the condensations of cartilages that form the lateral digits [63]. If this is indeed a general developmental pattern in mammals, with regards to the elongation of certain digits and the reduction of others, then the sthenurine hand might provide a clue to the sthenurine foot.

The sthenurine hand shows an elongation of the second, third, and fourth digit, accompanied by the reduction of digits one and five, while the digits of the hands of other macropodoids are of more equal length [12]. If this change in hand anatomy had been accomplished by the developmental process of suppressing the formation of the digits one and five (as described in pigs [63]) it is possible that this process was also transmitted to the hind limb. Sánchez-Villagra and Mencke [64] describe a similar echoing in the hind limb of a developmental change in the forelimb in the mole, *Talpa europa*: the transformation of the radial sesamoid into a prepollux in the hand is accompanied by the transformation of the tibial sesamoid into a (small) prehallux in the foot. They [64] propose that this change in foot morphology is not a functional adaptation (unlike the condition in the hand), but that it reflects a common epigenetic control of the hand and foot. Pedal digit one is lost in all macropodids, and digits two and three have been severely reduced, so in this respect the foot development has already been shifted from the basic pentadactyl pattern. But a new developmental program in the hand of sthenurines, resulting in a developmental reduction of the condensation cartilage for digit five, may also have found expression in the foot. If an animal like *Hadronomas w*as already functionally monodactyl, then there might have been no active selection pressure against such an anatomical change.

## Conclusions

The following is a scenario for the evolution of bipedal walking in sthenurines. The key to understanding sthenurine evolution is to realize that the forelimb and hind limb anatomy are linked in a way that channels their locomotor mode. Sthenurines inherited their “hopping anatomy”, with a long tibia and digitigrade foot posture while moving, from their macropodid ancestry; thus any change in locomotion from bipedal hopping would be within the constraints of this anatomy as a starting point. One of the characteristic features of at least the Pleistocene sthenurines is a forelimb that has been specialized for browsing, with the concomitant anatomy that would restrict them from putting the hand on the ground in a palmar stance, or from supporting their body weight on their forelimbs [12]. Hence the slow, pentapedal motion seen in extant large macropodines would be difficult or impossible. This presents a functional problem, because no mammal that hops today uses hopping for its slowest gait, and it seems unlikely on energetic and biomechanical terms that an animal as large as a sthenurine would be able to do this.

The middle to early late Miocene sthenurine *Rhizosthenurus flanneryi* was fairly large for a macropodid by today's standards (probably around 12–15 kg). While it retained the fifth toe, it also had some modifications of the ankle joint indicative of shifting its body weight to the medial side of the foot, a morphology that can be interpreted as weight-bearing on one leg at a time. *Rhizosthenurus* had a few modifications of its forelimbs indicative of the type of specialized behavior proposed for later sthenurines, such as enlarged epicondylar areas on the humerus for digital flexors [16]. The larger late late Miocene sthenurine *Hadronomas* (estimated body mass of 30 kg) also retained the fifth digit, but had other hind limb features resembling those of later sthenurines [42]. *Hadronomas* sometimes clusters with the macropodines in our analyses, and sometimes with the Pleistocene sthenurines, perhaps indicating an intermediate functional morphology. Although the hand of this animal is unknown, it had features of the scapula resembling those of later sthenurines, including a reduced supraspinous fossa and an enlarged coracoid process, which have been functionally implicated in Pleistocene sthenurines for reaching with the forearms [12].

Thus, the Miocene sthenurines, although still relatively small, showed postcranial modifications indicative of specialized browsing using their hands, and weight-bearing on one foot at a time. We propose that these animals, while likely still hopping for their faster locomotor gait, were starting to use some bipedal walking for locomoting at slow speeds: their capacity for slow pentapedal locomotion would be compromised by the more specialized forelimbs, and hopping at very slow speeds appears to be biomechanically impossible. Remember that bipedal walking has been observed in tree-kangaroos, so is certainly not an impossible gait for a macropodid. Additionally, sthenurines have been proposed to have been feeding with an upright trunk, and reaching up over their heads into tall vegetation. With this feeding posture, resorting to pentapedal motion to move to the next bush would mean frequent postural changes and energy expenditure. During their early evolutionary history sthenurines may have been performing some bipedal shuffling in order to move short distances, even if they retained hopping as their regular faster gait. Adaptations for bipedal posture for feeding would also preadapt them for maintaining this posture while moving and balancing their weight over a single limb (as is already indicated by the foot modifications shifting the body weight to the medial side of the foot).

The spinal anatomy of *Rhizosthenurus* and *Hadronomas* is unknown, but at some point sthenurines would have started to stabilize the lumbar region of their spine (as described by Wells and Tedford [12] in the large Pleistocene *Sthenurus stirlingi*), in order to support their trunk and stabilize their front end while foraging with an upright trunk. The lumbar vertebrae with greatly enlarged metapophyses and reduced transverse processes indicate an increase in size of the multifidous musculature and, concurrently, a decrease in size of the longissimus dorsi. However, this anatomy would pose a further conflict with locomotion, both pentapedal walking and bipedal hopping. With a stiff, shorter lumbar region they would no longer be able to arch the back to perform pentapedal locomotion (even if they could support their weight on their hands), and with the stiff lumbar spine and reduced longissimus musculature they would be unable to dorsiflex in the region of the anticlinal vertebra, as seen in modern kangaroo hopping locomotion.

The majority of the Pleistocene sthenurines were no larger than modern large *Macropus* species [6], still without severe biomechanical limitations for hopping (apart from the morphology of the lumbar spine). Such animals likely would have been able to do some slow hopping, but may have become increasingly reliant on bipedal walking for much of their daily locomotor repertoire. But, as reliance on bipedal walking grew, with concomitant adaptive morphology, they would have been able to increase their body size to outside of the range where hopping is biomechanically feasible (because of the safety factors involved with tendon strength, see [3]). Whether or not the largest sthenurines (especially the large species of *Procoptodon*) abandoned hopping altogether is unknown, and would require more data to attempt to determine.

A likely reason that hopping locomotion has not been questioned for sthenurines is their generally kangaroo-like hind limb, with features such as a long tibia that are characteristic of hopping mammals. But, remember that this is the anatomy that they inherited from their macropodid ancestry. And, if sthenurines were indeed engaging in bipedal walking, then the long tibiae would give them a long length of stride, which would be energetically efficient. The loss of the fifth digit is perhaps counterintuitive for an animal that might require more foot support over a single leg, but Murray [42] has proposed, based on pedal anatomy, that *Hadronomas* was already functionally monodactyl, and we propose here a possible developmental reason for the reduction of this digit to vestigial remnant. One puzzling aspect of sthenurine anatomy, in the context of this hypothesis of bipedal walking, is that the knees that face outwards rather than inwards. Humans differ from apes in their more knock-kneed (valgus) stance, which relates to their placing of the foot in the midline during locomotion, which aids with balance over the hips in bipedal walking [43]. Sthenurines, on the other hand, appear to have had a more bow-legged (vargus) stance. The sthenurine stance may relate to biomechanical issues arising from the very different anatomical starting point of a kangaroo to a hominid for adopting such a gait. Perhaps this stance was originally adopted in the context of foraging behavior: a wider stance might make walking more clumsy, but would provide a broader base for balancing while standing. Or perhaps there were issues relating to carrying large pouch young with a different type of locomotion, which entailed rotational torsion of the body rather than the pitching seen in hopping, which necessitated this stance.

In conclusion, although a fossilized trackway would be the only means of completely verifying our hypothesis of sthenurines using a bipedal striding gait, their anatomy is clearly different from that of large macropodines. Sthenurines lack the specialized features for fast hopping seen in macropodines and the differences in anatomy from the extant forms can be functionally related to locomotion bearing weight on one leg at a time.

## Supporting Information

Figure S1 **Ilium length versus other aspects of ilial morphology.** (A) Ilium length versus width of the tuber coxa. (B) Ilium length versus the dorsal length of the ischium. (C) Ilium length versus the ventral length of the puboischiatic symphysis. (D) Ilium length versus the length of the epipubic bone.
(TIF)

Figure S2 **Femur length versus other aspects of femoral morphology.** (A) Femur length versus the anterior-posterior width of the femoral head. (B) Femur length versus the distance of femoral head to the base of the lesser trochanter. (C) Femur length versus the length of the femur from the proximal end to the base of the adductor scar. (D) Femur length versus the width of the femur across the distal condyles.
(TIF)

Figure S3 **Other morphological variables from the tibia and pes.** (A) Tibia average midshaft diameter versus the anterior-posterior length of the tibia tuberosity. (B) Width (medio-lateral) of the base of the astragalus versus the width (dorso-plantar) of the fibular facet on the lateral trochlear ridge of the astragalus. (C) Width of the base of the astragalus versus the length of the calcaneal tuber. (D) Length of the femur versus the length of the fourth metatarsal.
(TIF)

Figure S4 **Scaling of long bone length versus diameter (shown in** **Figure 10** **) with labeled taxa.** (A) Femur length versus average femur cross-sectional diameter. (B) Tibia length versus average tibial midshaft cross sectional diameter.
(TIF)

Figure S5 **Additional discriminant analyses.** (A) Using all of the hind limb bones, for all of the taxa. (B) Without the pelvis, for all of the taxa.
(TIF)

Table S1 **Taxonomy of the Superfamily Macropodoidea.**
(DOC)

Table S2 **Measurements taken.**
(DOC)

Table S3 **Specimens measured for analyses.**
(DOC)

Table S4 **Specimens measured for calcaneum only analyses.**
(DOC)

Table S5 **Data for all of the bones.**
(PDF)

Table S6 **Additional data on the calcaneum.**
(PDF)

Text S1 **Description of bivariate plots.**
(DOC)

Text S2 **Additional discriminant analyses, using all of the taxa.** Table A, Coefficient loadings for additional Discriminant Analysis for all of the taxa: all hind limb bones. Table B, Coefficient loadings for additional Discriminant Analysis for all taxa: without the pelvis.
(DOC)
