# Supplementary figures and images for: Locomotion in Extinct Giant Kangaroos: Were Sthenurines Hop-Less Monsters?
Source: PLoS One. 2014 Oct 15;9(10):e109888. doi: 10.1371/journal.pone.0109888 (PMC4198187; doi:10.1371/journal.pone.0109888)

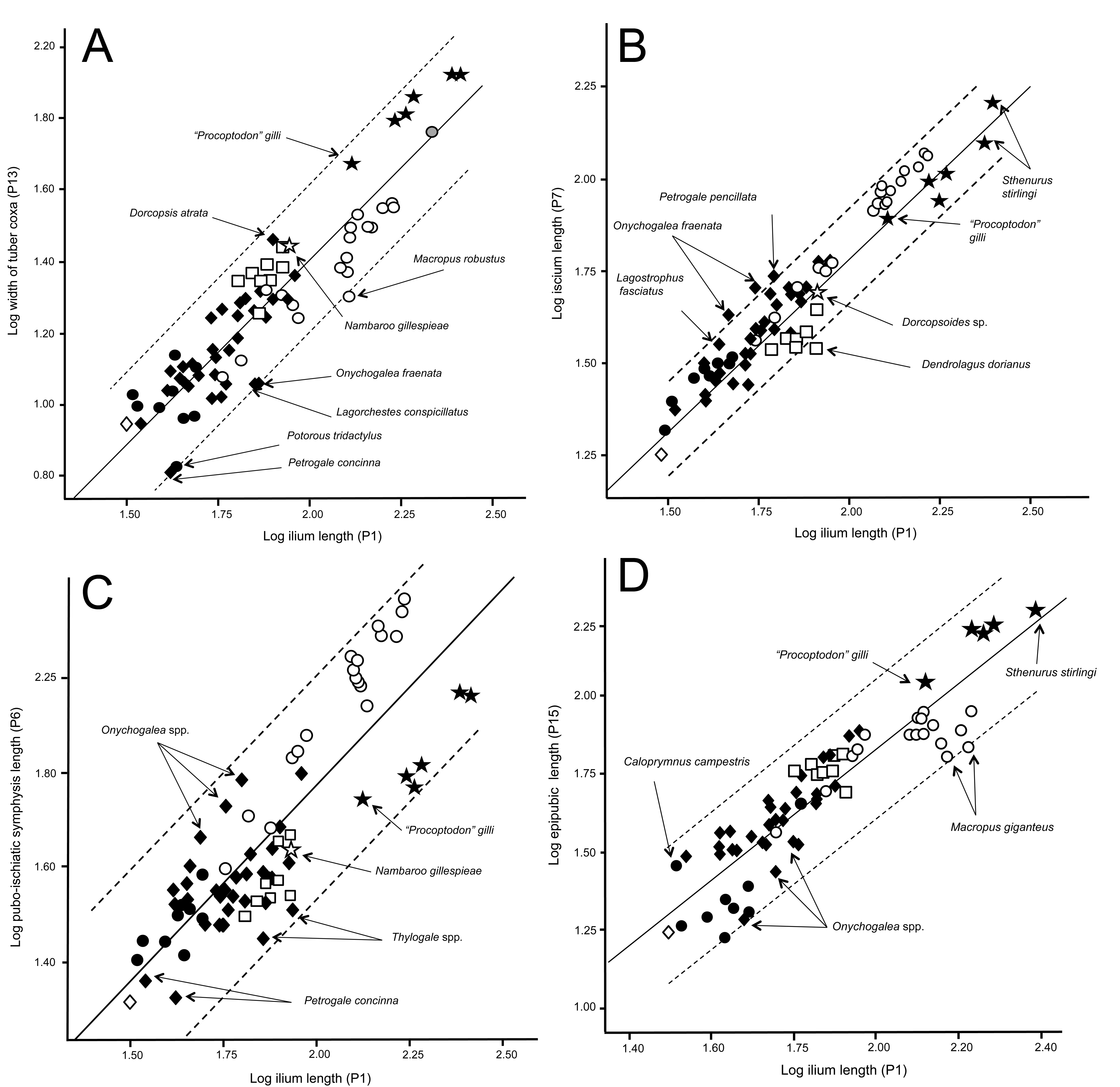

Supplement: Figure S1 — Ilium length versus other aspects of ilial morphology. (A) Ilium length versus width of the tuber coxa. (B) Ilium length versus the dorsal length of the ischium. (C) Ilium length versus the ventral length of the puboischiatic symphysis. (D) Ilium length versus the length of the epipubic bone. (TIF) [file pone.0109888.s001.tif]

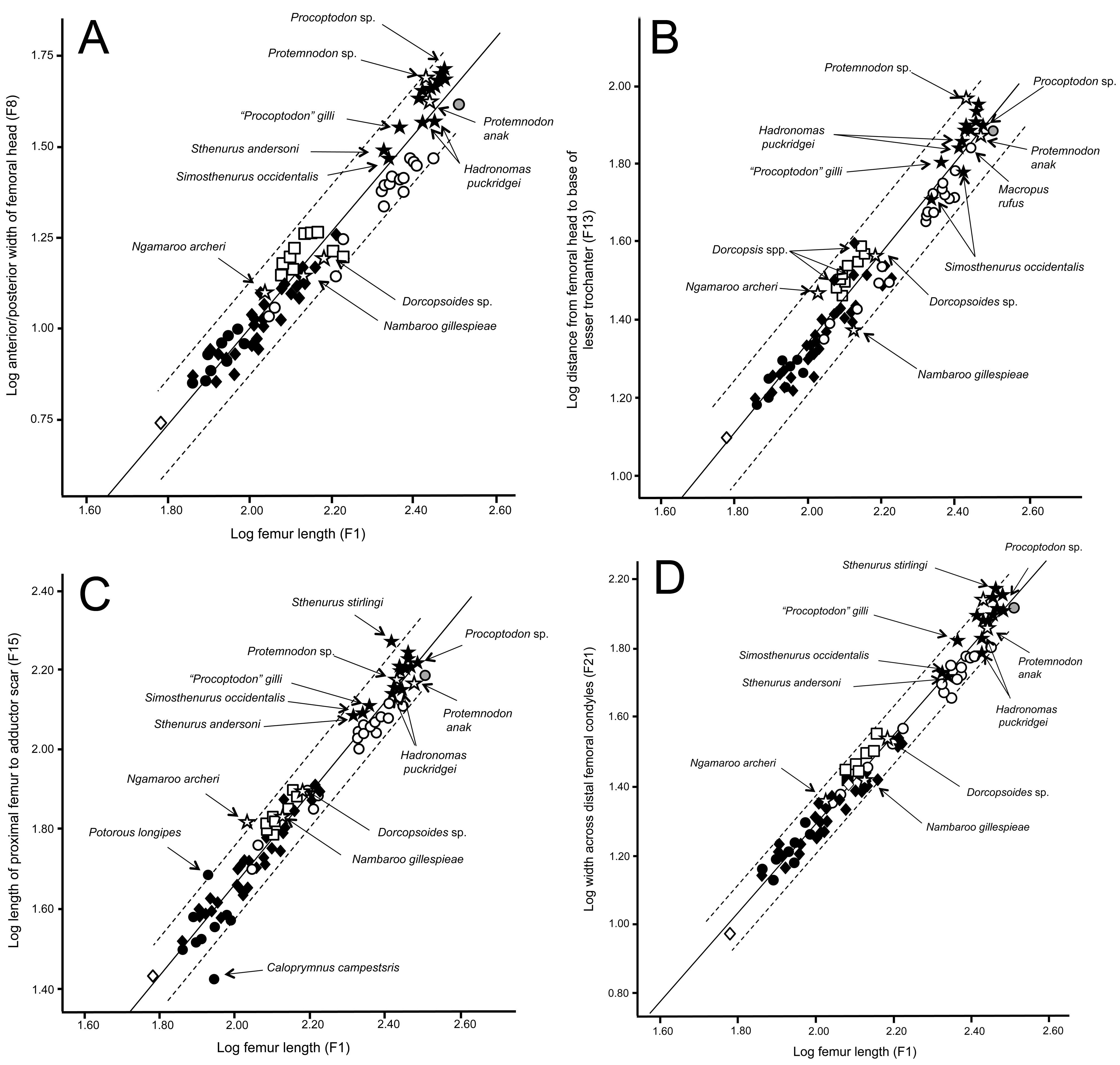

Supplement: Figure S2 — Femur length versus other aspects of femoral morphology. (A) Femur length versus the anterior-posterior width of the femoral head. (B) Femur length versus the distance of femoral head to the base of the lesser trochanter. (C) Femur length versus the length of the femur from the proximal end to the base of the adductor scar. (D) Femur length versus the width of the femur across the distal condyles. (TIF) [file pone.0109888.s002.tif]

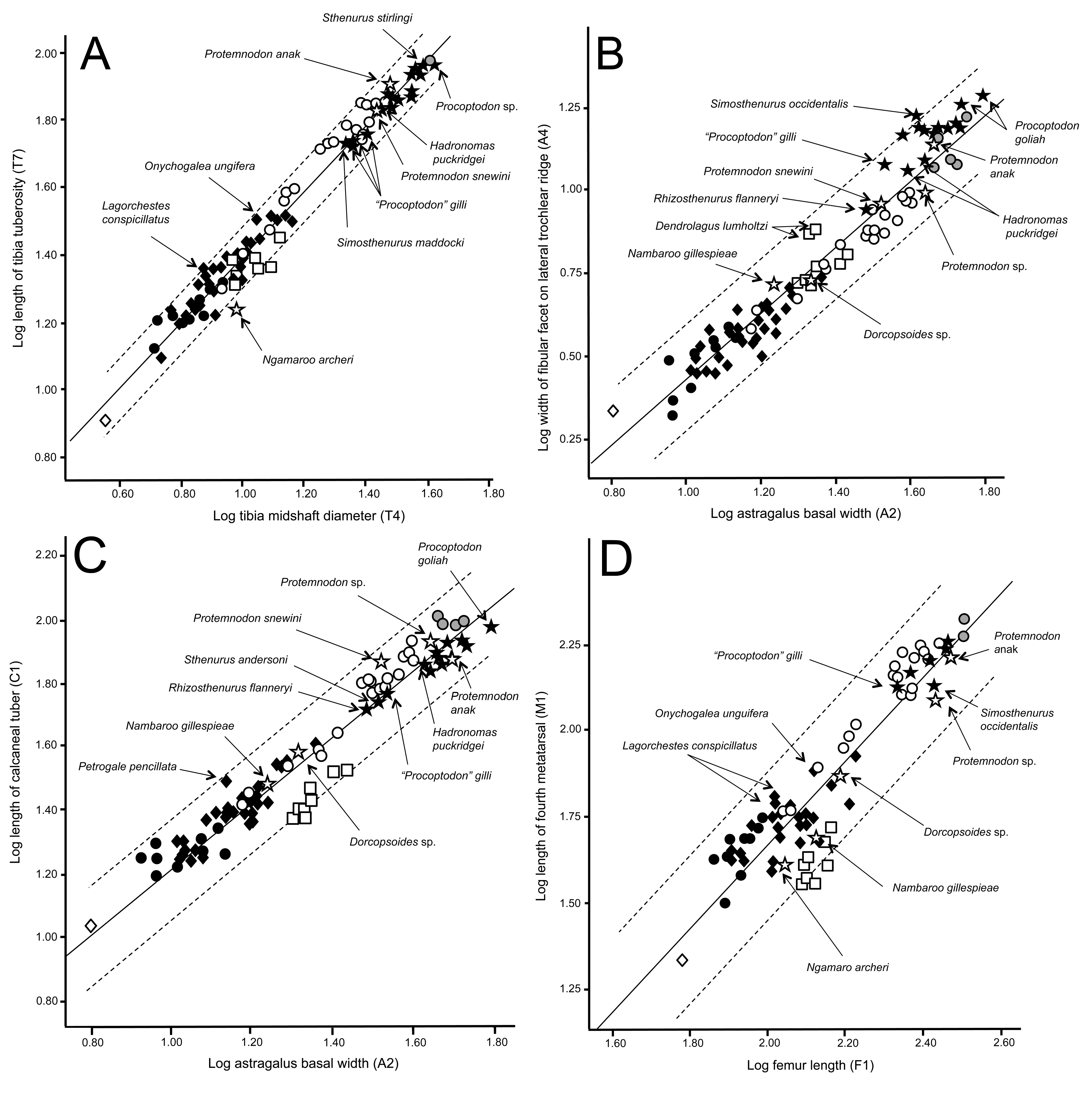

Supplement: Figure S3 — Other morphological variables from the tibia and pes. (A) Tibia average midshaft diameter versus the anterior-posterior length of the tibia tuberosity. (B) Width (medio-lateral) of the base of the astragalus versus the width (dorso-plantar) of the fibular facet on the lateral trochlear ridge of the astragalus. (C) Width of the base of the astragalus versus the length of the calcaneal tuber. (D) Length of the femur versus the length of the fourth metatarsal. (TIF) [file pone.0109888.s003.tif]

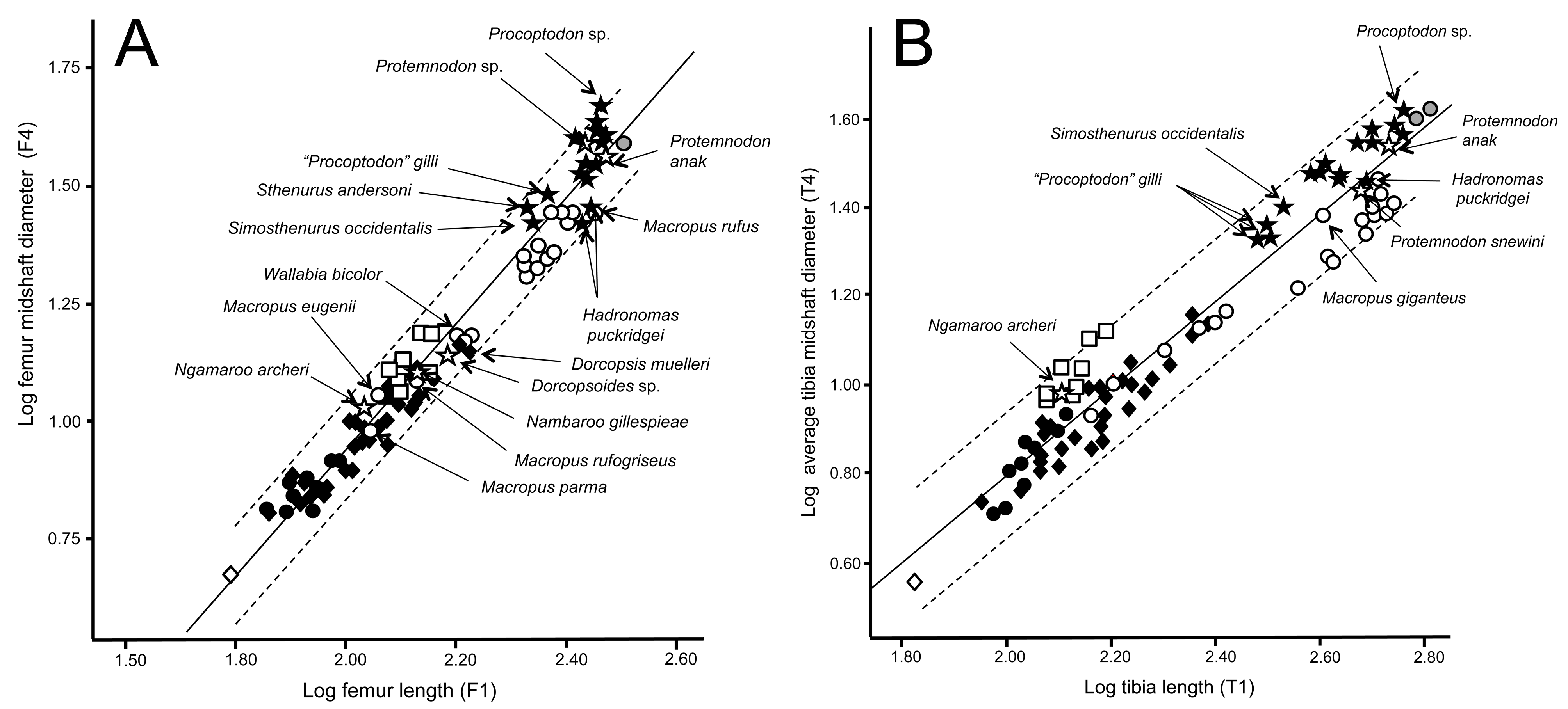

Supplement: Figure S4 — Scaling of long bone length versus diameter (shown in Figure 10 ) with labeled taxa. (A) Femur length versus average femur cross-sectional diameter. (B) Tibia length versus average tibial midshaft cross sectional diameter. (TIF) [file pone.0109888.s004.tif]

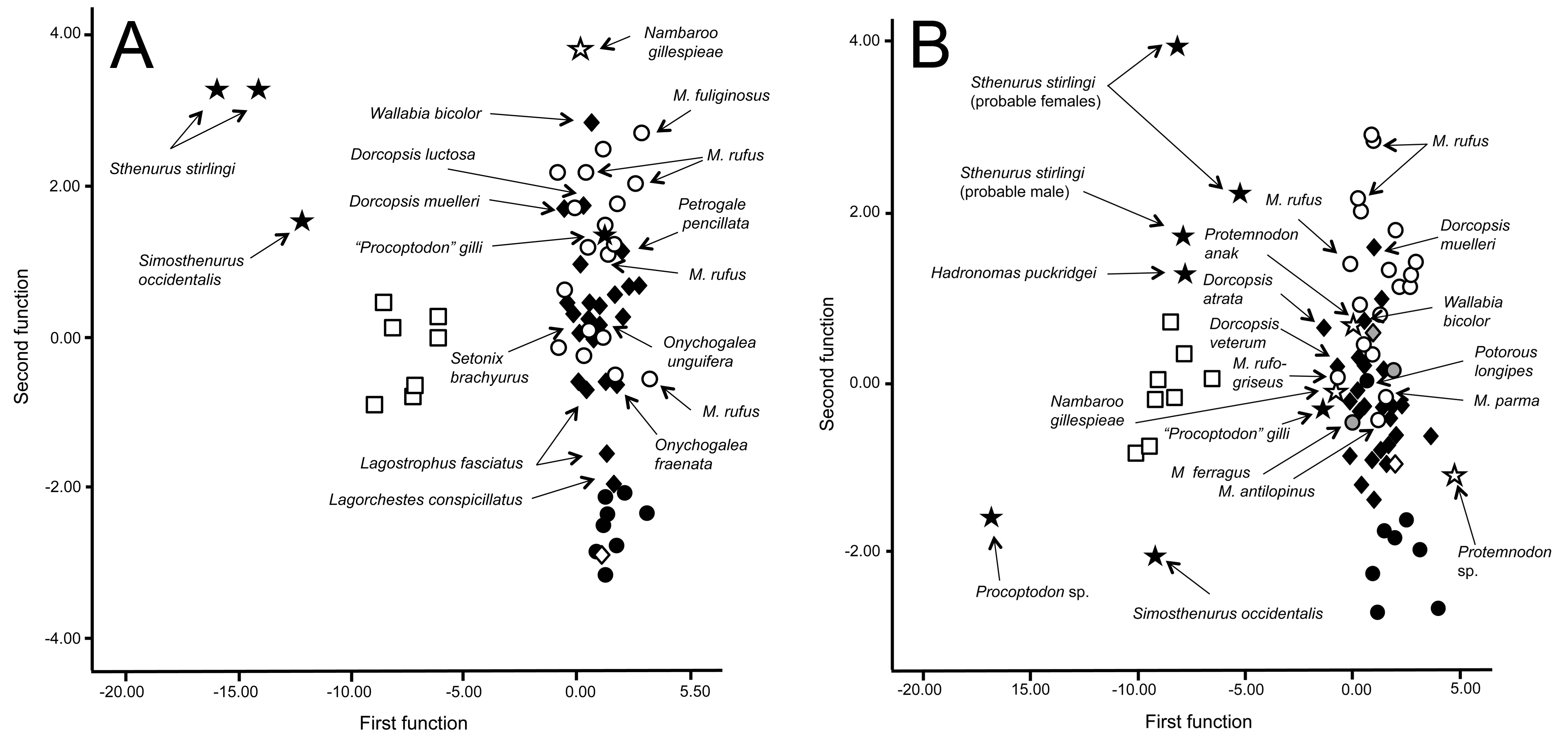

Supplement: Figure S5 — Additional discriminant analyses. (A) Using all of the hind limb bones, for all of the taxa. (B) Without the pelvis, for all of the taxa. (TIF) [file pone.0109888.s005.tif]
