# Supplementary material for: Locomotion in Extinct Giant Kangaroos: Were Sthenurines Hop-Less Monsters?
Source: PLoS One. 2014 Oct 15;9(10):e109888. doi: 10.1371/journal.pone.0109888 (PMC4198187; doi:10.1371/journal.pone.0109888)
Supplement: Table S1 — Taxonomy of the Superfamily Macropodoidea. (DOC) [file pone.0109888.s006.doc]

**Table S1: Taxonomy of the Superfamily Macropodoidea.**

Following Prideaux and Warburton [4]. ✝ = extinct taxon.

| Family | Subfamily | Included genera |
| --- | --- | --- |
| Balbaridae✝ |  | *Nambaroo*✝*, Balbaroo*✝*,* plus several other extinct genera |
| Hypsiprymno-  dontidae |  | *Hypsiprymnodon* (musky rat-kangaroo) plus several extinct  genera |
| Macropodidae | Potoroinae  (rat-kangaroos) | *Aepyprymnus* (rufous rat-kangaroo), *Bettongia* (bettongs), *Caloprymnus*✝(desert rat-kangaroo), *Potorous* (potoroos), plus  several other extinct genera |
|  | Lagostrophinae | *Lagostrophus* (banded hare-wallaby), *Troposodon*✝ |
|  | Bulungamayinae✝ | *Bulungamaya*✝*, Ganguroo*✝*,* plus several other extinct  genera. |
|  | Macropodinae | *Dendrolagus* (tree-kangaroos), *Dorcopsis*, *Dorcopsulus* (New Guinea forest-wallabies), *Lagorchestes* (hare-wallabies),  *Macropus* (“regular” kangaroos and wallabies), *Onychogalea*  (nail-tail wallabies), *Petrogale* (rock-wallabies), *Setonix* (quokka), *Thylogale* (pademelons), *Wallabia* (swamp wallaby), *Dorcopsoides*✝, *Protemnodon*✝, plus several other extinct  genera. |
|  | Stenurinae✝ | *Archaeosimus*✝, *Hadronomas*✝, *Metasthenurus*✝,  *Procoptodon*✝, *Rhizosthenurus*✝, *Sthenurus*✝, *Wanburoo*✝. |
