# Supplementary material for: Locomotion in Extinct Giant Kangaroos: Were Sthenurines Hop-Less Monsters?
Source: PLoS One. 2014 Oct 15;9(10):e109888. doi: 10.1371/journal.pone.0109888 (PMC4198187; doi:10.1371/journal.pone.0109888)
Supplement: Table S2 — Measurements taken. (DOC) [file pone.0109888.s007.doc]

**Table S2: Measurements taken.**

Pelvis:

P1 Length of the iliac blade on ventrolateral side, measured from the tip of the ilium to the closest point on the anterior rim of the acetabulum.

P2 Width of the iliac blade on ventrolateral side, measured at the level of the anterior rim of the acetabulum.

P3 Width of the iliac blade on ventrolateral side, at the point of maximum curvature of the ilium.

P4 Ventrolateral length of pubis, measured from the lowest point on the rim of the acetetabulum to the pubic symphysis.

P5 Medial-lateral length of the epipubic fossa, measured from the pubic symphsis to the iliopectineal process.

P6 Ventral length of the pubioischiatic symphysis (i.e., total anterior-posterior length along the base of the pelvis).

P7 Dorsal length of the ischium, measured from the posterior edge of the acetabulum to the posterior dorsal point of the ischium.

P8 Length of the iliac blade on dorsal side, measured from the tip of the ilium to the closest point on the anterior rim of the acetabulum.

P9 Minimum width of the iliac blade on dorsal side, measured at the indentation just anterior to the acetabulum.

P10 Length of iliac blade on medial surface from tip of ilium to posterior border of sacroiliac attachment.

P11 Width of the iliac blade on dorsal side, at the point of maximum curvature of the ilium.

P12 Minimum width of the tip of the iliac blade (tuber coxa) on the dorsal side.

P13 Maximum width of the tip of the iliac blade (tuber coxa) on the dorsal side.

P14 Dorsoventral length of the sacroiliac joint, measured on the medial side of the ilium.

P15 Maximum length of epipubic bone.

P16 Maximum width of the epipubic bone at the level of the midshaft projection.

P17 Dorsoventral length (height) of iliopectineal process.

P18 Mediolateral width of ischial tuberosity.

P19 Anteroposterior width of ischial tuberosity.

P20 Anterior-posterior width of obturator foramen.

P21 Dorsal–ventral width of obturator foramen.

P22 Dorsal–ventral length of ischium (not including pelvic tubercule, often missing in fossils).

Femur:

F1 Articular length of femur (from femoral head to femoral condyles).

F2 Anteroposterior width of femur below adductor scar (used to create F4).

F3 Mediolateral width of femur below adductor scar (used to create F4).

F4 Average midshaft width of femur

F5 Length of greater trochanter (from dorsal tip of trochanter to level of base of femoral condyle).

F6 Distance between tip of greater trochanter and anterior border of femoral head.

F7 Mediolateral width of femoral head.

F8 Anteroposterior width of femoral head.

F9 Length of lesser trochanter (dorsal – ventral).

F10 Maximum height of lesser trochanter above femoral shaft.

F11 Length of gluteal attachment on greater trochanter.

F12 Maximum width of greater trochanteric ridge of gluteal attachment.

F13 Distance from femoral head to base of lesser trochanter.

F14 Dorsoventral length of scar for adductor attachment.

F15 Length of femur from proximal end (between greater trochanter and femoral head) to base of adductor scar.

F16 Maximum width between the patellar trochanteric ridges.

F17 Width of lateral tibial condyle measured on posterior side.

F18 Width of medial tibial condyle measured on posterior side.

F19 Length of lateral tibial condyle measured on lateral side.

F20 Length of medial tibial condyle measured on medial side.

F21 Maximum with across tibial condyles on ventral side of femur.

F22 Length of lateral trochanteric ridge of patella.

F23 Length of medial trochanteric ridge of patella.

F24 Length of patellar groove.

Tibia

T1 Maximum articular length.

T2 Midshaft anterioposterior width (used to create T4).

T3 Midshaft mediolateral width (used to create T4).

T4 Average midshaft width.

T5 Length (dorsal–ventral) length of tibial (cnemial) crest.

T6 Height of tibial crest above shaft of tibia.

T7 Length of proximal articular surface from posterior border of femoral condyles to tibial crest.

T8 Width of proximal articular surface across femoral condyles.

T9 Maximum mediolateral width of distal tibia.

T10 Anteroposterior width of lateral astragalar facet.

T11 Mediolateral width of lateral astragalar facet.

T12 Anteroposterior width of medial astragalar facet (= medial malleolus).

T13 Mediolateral width of medial astragalar facet (= medial malleolus) (to lateral edge of tibia, inc. non-articular portion).

T14 Height of medial astragalar facet (= medial malleolus).

T15 Anteroposterior width of distal articular surface including posterior astragalar process.

Astragalus:

A1 Maximum dorsoventral length, measured from top of astragalus to base of astragalonavicular facet.

A2 Maximum mediolateral width, from lateral rim of ridge (= lateral trochlea) for astragalotibial facet to medial rim (= medial trochlea) of astragalofibular facet.

A3 Anteroposterior width of medial trochlear ridge of astragalotibial facet (= medial tibial facet), measured in medial view.

A4 Anteroposterior width of lateral trochlear ridge of astragalotibial facet (= fibular facet), measured in lateral view.

A5 Length of midline groove of lateral astragalotibial facet.

A6 Maximum width between medial and lateral ridges of lateral astragalotibial facet (measured on outside of ridges).

A7 Anteroposterior length of medial astragalotibial facet.

A8 Mediolateral width of medial astragalotibial facet.

A9 Maximum chord length of lateral ridge of lateral astragalotibial facet.

A10 Maximum chord length of medial ridge of lateral astragalotibial facet.

Calcaneum:

C1 Maximum plantar (posterior) anterioposterior (dorsoventral) length (measured from tip of calcaneal tuber to posterior border of calcaneocuboid facet).

C2 Maximum lateral anterioposterior (dorsoventral) length (measured from tip of calcaneal tuber to anterior border of lateral calcaneocuboid facet).

C3 Maximum medial anterioposterior (dorsoventral) length (measured from tip of calcaneal tuber to medial anterior border of calcaneocuboid facet).

C4 Dorsoplantar width of midshaft of calcaneal tuber.

C5 Mediolateral width of midshaft of calcaneal tuber measured on anterior (volar) side (main dorsal ridge only).

C6 Dorsoplantar width of top of calcaneal tuber.

C7 Mediolateral width of top of calcaneal tuber.

C8 Anteroposterior (dorsoventral) width of fibular facet.

C9 Mediolateral width of fibular facet.

C10 Anterposterior (dorsoventral) length of calcaneal head on lateral side, from top of fibular facet to base of lateral cubonavicular facet.

C11 Anterposterior (dorsoventral) length continuous lower ankle joint (CLAJ) measured

from the top of the ectal facet to the bottom of joint articulation.

C12 Anterioposterior (dorsoventral) length of calcaneal heel (measured from tip of calcaneal tuber to base of sustentacular facet).

C13 Mediolateral width across CLAJ.

C14 Maximum width across calcaneal head, measured on posterior (plantar) side.

C15 Length (mediolateral) of ectal facet.

C16 Mediolateral width of midshaft of calcaneal tuber measured on posterior (plantar) side.

C17 Width of sulcus for tendon of peroneus longus (taken as inside measurement with other side of calipers).

C18 Width of sulcus for tendon of flexor digitorum longus (taken as inside measurement with other side of calipers).

C19 Length of the ridge along the lateral/dorsal side of the sustentaculum tali.

C20 Width (mediolateral) across anterior surface of cubonavicular facets.

C21 Length (anteroposterior/dorsoplantar) across surface of cubnavicular facets.

C22 Width (mediolateral) across posterior base of calcaneum, including the cubonavicular facets, and the base of the sustentacular ridge (if flush with CN facet).

C23 Width (dorsoplantar) of lateromedial cubonavicular facet.

C24 Width (mediolateral) of the lateromedial cubonavicular facet.

C25 Length of the roughened area on the plantar side of the calcaneal heel.

Fourth Metatarsal

M1 Maximum length.

M2 Anteroposterior midshaft width.

M3 Mediolateral midshaft width.

M4 Proximal articular surface mediolateral width.

M5 Distal articular surface mediolateral width.

Fifth Metatarsal

M5-1 Maximum length.

M5-2 Anteroposterior midshaft width.

M5-3 Mediolateral midshaft width.

First Phalanx

Ph1L Maximum length

Ph1W Mediolateral midshaft width

Second Phalanx

Ph2L Maximum length

Ph2W Mediolateral midshaft width

Third Phalanx

Ph3L Maximum length

Ph3W Mediolateral proximal articular width
