# Supplementary material for: Locomotion in Extinct Giant Kangaroos: Were Sthenurines Hop-Less Monsters?
Source: PLoS One. 2014 Oct 15;9(10):e109888. doi: 10.1371/journal.pone.0109888 (PMC4198187; doi:10.1371/journal.pone.0109888)
Supplement: Table S3 — Specimens measured for analyses. (DOC) [file pone.0109888.s008.doc]

**Table S3 Specimens measured for analyses (for all of the bones).**

| FAMILY | SUBFAMILY | TAXON | C/P?^1^ | SPECIMEN NO. |
| --- | --- | --- | --- | --- |
| Balbaridae✝ |  | *Nambaroo gillespeiae*✝ | P^2^ | QM F35432 |
| Hypsiprymnodontidae |  | *Hypsiprymnodon moschatus* | C | SAM M11940 |
| Macropodidae | Potoroinae | *Aepyprymnus rufescens* | C* | AMNH 65283 |
| Macropodidae | Potoroinae | *Aepyprymnus rufescens* | C | QM J5579 |
| Macropodidae | Potoroinae | *Bettongia lesueur* | C | AM M2132 |
| Macropodidae | Potoroinae | *Bettongia tropica* | C | AM M40067 |
| Macropodidae | Potoroinae | *Bettongia penicillata* | C | SAM: M18986 |
| Macropodidae | Potoroinae | *Caloprymnus campestris*✝ | C^3^ | NT 48050 |
| Macropodidae | Potoroinae | *Potorous longipes* | C | NMV C32723 |
| Macropodidae | Potoroinae | *Potorous tridactylus* | C | AMNH 65297 |
| Macropodidae | incertae sedis | *Ngamaroo archeri*✝ | P^4^ | SAM P23821 |
| Macropodidae | Lagostrophinae | *Lagostrophus fasciatus* | C | WAM M16285 |
| Macropodidae | Lagostrophinae | *Lagostrophus fasciatus* | C | WAM M4393 |
| Macropodidae | Lagostrophinae | *Lagostrophus fasciatus* | C | AM M40303 |
| Macropodidae | Macropodinae | *Dendrolagus benettianus* | C | WAM M5530 |
| Macropodidae | Macropodinae | *Dendrolagus dorianus* | P^5^ | WAM M7643 |
| Macropodidae | Macropodinae | *Dendrolagus dorianus* | C | AM M9109 |
| Macropodidae | Macropodinae | *Dendrolagus goodfellowi* | C | NMV C25092 |
| Macropodidae | Macropodinae | *Dendrolagus lumholtzi* | C* | AMNH 65265 |
| Macropodidae | Macropodinae | *Dendrolagus lumholzi* | C* | SAM M7206 |
| Macropodidae | Macropodinae | *Dendrolagus matschiei* | C | WAM M21013 |
| Macropodidae | Macropodinae | *Dendrolagus matschiei* | C | QM J5287 |
| Macropodidae | Macropodinae | *Dendrolagus scotti* | P^6^ | AM M24424 |
| Macropodidae | Macropodinae | *Dorcopsis atrata* | P^7^ | AM M19461 |
| Macropodidae | Macropodinae | *Dorcopsis luctuosa* | C | SAM M15178 |
| Macropodidae | Macropodinae | *Dorcopsis muelleri* | P^8^ | AM M32339 |
| Macropodidae | Macropodinae | *Dorcopsis muelleri* | P^8^ | AM M32341 |
| Macropodidae | Macropodidae | *Dorcopsis veterum* | C* | AMNH 22262 |
| Macropodidae | Macropodinae | *Dorcopsulus vanheurni* | P^9^ | AMNH 194790 |
| Macropodidae | Macropodinae | *Lagorchestes conspicillatus* | C* | WAM M7032 |
| Macropodidae | Macropodinae | *Lagorchestes conspicillatus* | C | AMNH 197695 |
| Macropodidae | Macropodinae | *Lagorchestes hirsutus* | C | SAM M3587 |
| Macropodidae | Macropodinae | *Lagorchestes hirsutus* | C* | AM M40038 |
| Macropodidae | Macropodinae | *Macropus agilis* | C | AMNH 35750 |
| Macropodidae | Macropodinae | *Macropus antilopinus* | C | AMNH 70499 |
| Macropodidae | Macropodinae | *Macropus dorsalis* | C* | NMV C6490 |
| Macropodidae | Macropodinae | *Macropus eugenii* | C* | NMV C7908 |
| Macropodidae | Macropodinae | *Macropus fuliginosus* | C* | SAM M21497 |
| Macropodidae | Macropodinae | *Macropus fuliginosus* | C* | SAM M16578 |
| Macropodidae | Macropodinae | *Macropus giganteus* | C* | AMNH 35747 |
| Macropodidae | Macropodinae | *Macropus giganteus* | C | AMNH 35186 |
| Macropodidae | Macropodinae | *Macropus giganteus* | C | QM J11525 |
| Macropodidae | Macropodinae | *Macropus parma* | C* | SAM M7192 |
| Macropodidae | Macropodinae | *Macropus parryi* | C | AMNH 65054 |
| Macropodidae | Macropodinae | *Macropus robustus* | C | SAM M3695 |
| Macropodidae | Macropodinae | *Macropus robustus* | C* | AMNH 65036 |
| Macropodidae | Macropodinae | *Macropus rufogriseus* | C | AMNH 65116 |
| Macropodidae | Macropodinae | *Macropus rufus* | C | SAM M6559 |
| Macropodidae | Macropodinae | *Macropus rufus* | C | AMNH 200473 |
| Macropodidae | Macropodinae | *Macropus rufus* | C | AMNH 70284 |
| Macropodidae | Macropodinae | *Macropus rufus* | C | QM J22115 |
| Macropodidae | Macropodinae | *Macropus ferragus*✝ | P^10, 11, 19^ | NMV unnumbered^11^ |
| Macropodidae | Macropodinae | *Macropus ferragus*✝ | P^12, 11, 19^ | NMV unnumbered^13^ |
| Macropodidae | Macropodinae | *Macropus* cf *ferragus*✝ | P^14^ | SAM P194676 |
| Macropodidae | Macropodinae | *Macropus* cf *ferragus*✝ | P^15^ | SAM P43039 |
| Macropodidae | Macropodinae | *Macropus titan*✝ | P^16, 19^ | NMV unnumbered^17^ |
| Macropodidae | Macropodinae | *Macropus titan*✝ | P^18, 19^ | AMNH 18362 |
| Macropodidae | Macropodinae | *Onychogalea fraenata* | C* | NMV C6500 |
| Macropodidae | Macropodinae | *Onychogalea fraenata* | C | AMNH 42959 |
| Macropodidae | Macropodinae | *Onychogalea unguifera* | C* | WAM M11622 |
| Macropodidae | Macropodinae | *Petrogale assimilis* | C | QM J4470 |
| Macropodidae | Macropodinae | *Petrogale concinna* | P^20^ | WAM M9360 |
| Macropodidae | Macropodinae | *Petrogale concinna* | P^21^ | NMV C6478 |
| Macropodidae | Macropodinae | *Petrogale lateralis* | C* | AM M24183 |
| Macropodidae | Macropodinae | *Petrogale penicillata* | C* | AMNH 65261 |
| Macropodidae | Macropodinae | *Petrogale xanthropus* | C | AMNH 35642 |
| Macropodidae | Macropodinae | *Petrogale xanthropus* | C* | WAM M11469 |
| Macropodidae | Macropodinae | *Setonix brachyurus* | C | WAM M6792 |
| Macropodidae | Macropodinae | *Setonix brachyurus* | C | NMV C23029 |
| Macropodidae | Macropodinae | *Thylogale billardierii* | C* | NMV C11311 |
| Macropodidae | Macropodinae | *Thylogale billardierii* | P^22^ | NMV C11326 |
| Macropodidae | Macropodinae | *Thylogale billardierii* | C | NMV C30607 |
| Macropodidae | Macropodinae | *Thylogale stigmatica* | C* | AMNH 65153 |
| Macropodidae | Macropodinae | *Thylogale thetis* | P^23^ | AM M51512 |
| Macropodidae | Macropodinae | *Wallabia bicolor* | C* | AMNH 65722 |
| Macropodidae | Macropodinae | *Dorcopsoides*✝ sp. | P^24^ | NT P890 |
| Macropodidae | Macropodinae | *Protemnodon anak*✝ | P^25, 29^ | NMV |
| Macropodidae | Macropodinae | *Protemnodon anak*✝ | P^26^ | QM F14675 |
| Macropodidae | Macropodinae | *Protemnodon snewini*✝ | P^27^ | QM F9075/9076 |
| Macropodidae | Macropodinae | *Protemnodon*✝ sp. | P^28, 29^ | AMNH 117494 |
| Macropodidae | Sthenurinae✝ | *Hadronomas puckridgi*✝ | P^30^ | NT MPA61 |
| Macropodidae | Sthenurinae✝ | *Hadronomas puckridgi*✝ | P^31^ | NT P98141 |
| Macropodidae | Sthenurinae✝ | *Hadronomas puckridgi*✝ | P^32^ | NT MP1279 |
| Macropodidae | Sthenurinae✝ | *Hadronomas puckridgi*✝ | P^32^ | NT MP1200 |
| Macropodidae | Sthenurinae✝ | *Hadronomas puckridgi*✝ | P^33^ | NT unnumbered |
| Macropodidae | Sthenurinae✝ | *Hadronomas puckridgi*✝ | P^34^ | NT GB5-5 |
| Macropodidae | Sthenurinae✝ | *Hadronomas puckridgi*✝ | P^34^ | NT P—2911dW |
| Macropodidae | Sthenurinae✝ | *Hadronomas puckridgi*✝ | P^35^ | NT MPUD-07/72 |
| Macropodidae | Sthenurinae✝ | *Hadronomas puckridgi*✝ | P^36^ | NT P9336 |
| Macropodidae | Sthenurinae✝ | *Hadronomas puckridgi*✝ | P^36^ | NT SP69 |
| Macropodidae | Sthenurinae✝ | *Hadronomas puckridgi*✝ | P^36^ | NT A122 |
| Macropodidae | Sthenurinae✝ | *Hadronomas puckridgi*✝ | P^36^ | NT unnumbered |
| Macropodidae | Sthenurinae✝ | *Hadronomas puckridgi*✝ | P^36^ | NT P9214 |
| Macropodidae | Sthenurinae✝ | *Hadronomas puckridgi*✝ | P^37^ | NT P87-1422 |
| Macropodidae | Sthenurinae✝ | *Hadronomas puckridgi*✝ | P^38^ | NT SP708 |
| Macropodidae | Sthenurinae✝ | *Hadronomas puckridgi*✝ | P^38^ | NT SP488 |
| Macropodidae | Sthenurinae✝ | *Hadronomas puckridgi*✝ | P^38^ | NT P2960 |
| Macropodidae | Sthenurinae✝ | *Hadronomas puckridgi*✝ | P^38^ | NT MP-162-4 |
| Macropodidae | Sthenurinae✝ | *Hadronomas puckridgi*✝ | P^38^ | NT P9261 |
| Macropodidae | Sthenurinae✝ | *Hadronomas puckridgi*✝ | P^39^ | NT MP328 |
| Macropodidae | Sthenurinae✝ | *Hadronomas puckridgi*✝ | P^39^ | NT unnumbered |
| Macropodidae | Sthenurinae✝ | *Hadronomas puckridgi*✝ | P^39^ | NT P887-15 |
| Macropodidae | Sthenurinae✝ | *Hadronomas puckridgi*✝ | P^39^ | NT P887-18 |
| Macropodidae | Sthenurinae✝ | *Rhizosthenurus flanneryi*✝ | P^40^ | QM F31456 |
| Macropodidae | Sthenurinae✝ | *Procoptodon*✝ sp. | P^41^ | NMV unnumbered |
| Macropodidae | Sthenurinae✝ | *Procoptodon goliah*✝ | P^42^ | NMV unumbered |
| Macropodidae | Sthenurinae✝ | *“Procoptodon” browneorum*✝ | P^43^ | WAM 65-4-78 |
| Macropodidae | Sthenurinae✝ | *“Procoptodon” browneorum*✝ | P^44^ | WAM 68-3-637 |
| Macropodidae | Sthenurinae✝ | *“Procoptodon” gilli*✝ | P^47^ | SAM P17469 |
| Macropodidae | Sthenurinae✝ | *“Procoptodon” gilli*✝ | P^46^ | SAM P17277 |
| Macropodidae | Sthenurinae✝ | *“Procoptodon” gilli*✝ | P^46^ | FU unnumbered |
| Macropodidae | Sthenurinae✝ | *“Procoptodon” gilli*✝ | P^46^ | SAM P17291 |
| Macropodidae | Sthenurinae✝ | *“Procoptodon” gilli*✝ | P^47^ | SAM unnumbered |
| Macropodidae | Sthenurinae✝ | *“Procoptodon” gilli*✝ | P^48^ | SAM P17528 |
| Macropodidae | Sthenurinae✝ | *Simosthenurus maddocki*✝ | P^49^ | SAM P1A1 |
| Macropodidae | Sthenurinae✝ | *Simosthenurus occidentalis*✝ | C | SAM P20820 |
| Macropodidae | Sthenurinae✝ | *Simosthenurus occidentalis*✝ | P^50^ | SAM P17258 |
| Macropodidae | Sthenurinae✝ | *Simosthenurus occidentalis*✝ | P^50^ | SAM P17472 |
| Macropodidae | Sthenurinae✝ | *Simosthenurus occidentalis*✝ | P^51^ | SAM P17474 |
| Macropodidae | Sthenurinae✝ | *Simosthenurus occidentalis*✝ | P^51^ | SAM P17474.1 |
| Macropodidae | Sthenurinae✝ | *Simosthenurus occidentalis*✝ | P^51^ | SAM P17472 |
| Macropodidae | Sthenurinae✝ | *Simosthenurus occidentalis*✝ | P^51^ | SAM P18298 |
| Macropodidae | Sthenurinae✝ | *Simosthenurus occidentalis*✝ | P^51^ | SAM unnumbered |
| Macropodidae | Sthenurinae✝ | *Simosthenurus occidentalis*✝ | P^52^ | SAM P17262 |
| Macropodidae | Sthenurinae✝ | *Simosthenurus occidentalis*✝ | P^52^ | SAM P17296 |
| Macropodidae | Sthenurinae✝ | *Simosthenurus occidentalis*✝ | P^52^ | SAM P17475 |
| Macropodidae | Sthenurinae✝ | *Simosthenurus occidentalis*✝ | P^52^ | SAM P17460 |
| Macropodidae | Sthenurinae✝ | *Simosthenurus occidentalis*✝ | P^53^ | WAM 65-4-133 |
| Macropodidae | Sthenurinae✝ | *Simosthenurus occidentalis*✝ | P^54^ | WAM unnumbered |
| Macropodidae | Sthenurinae✝ | *Simosthenurus occidentalis*✝ | P^54^ | SAM P40068 |
| Macropodidae | Sthenurinae✝ | *Simosthenurus occidentalis*✝ | P^55^ | SAM P8296 |
| Macropodidae | Sthenurinae✝ | *Simosthenurus occidentalis*✝ | P^56^ | WAM 65-4-131 |
| Macropodidae | Sthenurinae✝ | *Simosthenurus occidentalis*✝ | P^56^ | WAM 65-4-62 |
| Macropodidae | Sthenurinae✝ | *Simosthenurus occidentalis*✝ | P^56^ | WAM 65-4-64 |
| Macropodidae | Sthenurinae✝ | *Simosthenurus occidentalis*✝ | P^56^ | WAM 65-4-66 |
| Macropodidae | Sthenurinae✝ | *Simosthenurus occidentalis*✝ | P^56^ | WAM 65-4-68 |
| Macropodidae | Sthenurinae✝ | *Sthenurus andersoni*✝ | P^57^ | SAM P13673 |
| Macropodidae | Sthenurinae✝ | *Sthenurus stirlingi*✝ | P^58^ | AMNH 140809 |
| Macropodidae | Sthenurinae✝ | *Sthenurus stirlingi*✝ | P^59^ | SAM P17259 |
| Macropodidae | Sthenurinae✝ | *Sthenurus stirlingi*✝ | P^60^ | NMV unnumbered |
| Macropodidae | Sthenurinae✝ | *Sthenurus stirlingi*✝ | P^6^ | AMNH 117496 |
| Macropodidae | Sthenurinae✝ | *Sthenurus stirlingi*✝ | P^62^ | AMNH 117497 |
| Macropodidae | Sthenurinae✝ | *Sthenurus stirlingi*✝ | P^62^ | AMNH 117494A |
| Macropodidae | Sthenurinae✝ | *Sthenurus tindalei*✝ | P^63^ | AMNH 117499 |
| Macropodidae | Sthenurinae✝ | *Sthenurus tindalei*✝ | P^64^ | AMNH 117493 |
| Macropodidae | Sthenurinae✝ | *Sthenurus*✝ sp*.* | P^65^ | SAM unnumbered |

✝ = extinct taxon.

Acronyms for museums:

AM = Australian Museum. AMNH = American Museum of Natural History. FU = Flinders University. QM = Queensland Museum. NMV = National Museum Victoria. NT = Northern Territories Museum. SAM = South Australian Museum. WAM = Western Australian Museum.

Notes:

1. C/P? = Complete or partial? Partial individuals are included in the bivariate plots only, unless otherwise stated. C* = complete except for some estimates of distal phalanges (see text).

2. *Nambaroo gillespieae* is an almost complete specimen (see Kear, 2002): for the purposes of retaining it in the multivariate analyses, the following measurements (see Table S1) were added from the similarly-sized *Dorcopsis* *veterum* (dorcopsids are the most plesiomorphic of the macropodines: see Prideaux and Warburton, 2010): P7, P10, P15, P18, P19, P22, T1, T2, T3, T5, T6, M3, M3, Ph1, Ph2.

3. *Caloprymnus campestris*, the desert rat kangaroo, is recently extinct (not counted here as an extinct taxon), and its locomotor behavior was observed (extremely cursorial).

4. Complete tibia and calcaneum only.

5. Pelvis and tibia only.

6. Missing pelvis and phalanges.

7. Partial pelvis only, missing third phalanx.

8. These two specimens of *Dorcopsis muelleri* were combined to create a composite specimen for the multivariate analyses.

9. Missing footbones.

10. Footbones, parts of pelvis, tibia and calcaneum.

11. From Lake Victoria.

12. Partial femur, tibia, calcaneum; complete astragalus.

13. From Lake Victoria site 50.

14. Astragalus and calcaneum only.

15. Astragalus only.

16. Partial pelvis only, missing tibia.

17. Composite individual from Lancefield.

18. Partial pelvis only.

19. Composite specimen of *Macropus titan/ferragus* created from femur of AMNH *M. titan*, tibiae of the two specimens of *M. ferragus* from Lake Victoria, astragalus, calcaneum and foot bones of *M. titan* from Lancefield.

20. Missing astragalus, calcaneum, and foot bones.

21. Missing tibia, astragalus, calcaneum, and foot bones.

22. Missing astragalus, calcaneum, and foot bones.

23. Partial pelvis only.

24. Complete pes, partial measurements on pelvis and femur.

25. Composite of unnumbered bones from same site. All bones except for pelvis. Used for tibia of *Protemnodon* sp. composite.

26. Calcaneum and foot bones only.

27. Tibia and pes.

28. Femur, partial tibia, pes.

29. These two used to create “*Protemnodon* sp. composite”. (Tibia from *P. anak*, the rest from *P.* sp.)

30. Femur only: used to create *Hadronomas* composite.

31. Femur only.

32. Partial tibia only

33. Astragalus only

34. Femur only, averaged for *Hadronomus* composite.

35. Tibia only. Used for *Hadronomas* composite.

36. Astragalus only, averaged for *Hadronomas* composite.

37. Calcaneum only, averaged for *Hadronomas* composite.

38. Metatarsal IV only, averaged for *Hadronomas* composite.

39. Metatarsal V only.

40. First phalanx only, averaged for *Hadronomas* composite.

41. Second phalanx only, average for *Hadronomas* composite.

40. Astragalus, calcaneum and foot bones only.

41. Femur only, used to make *Procoptodon* composite.

42. Composite tibia and pes from Lake Victoria, used to make *Procoptodon* composite.

43. Astragalus and alcaneum only. Used to make foot for composite *“P”. gilli* (see text).

44. Metatarsal IV and foot bones only. Used to make foot for composite *“P”. gilli* (see text).

45. Pelvis only, used for composite *“P”. gilli* (see text).

46. Tibia only.

47. Partial femur, and fourth metatarsal only, used for metarsal of composite *“P”. gilli* (see text).

48. Femur and tibia, used for composite *“P”. gilli* (see text).

49. Partial tibia only.

50. Pelvis only.

51. Femur only.

52. Tibia only.

53. Astragalus only.

54. Astragalus and fourth metatarsal only

55. Fourth metatarsal only.

56. Calcaneum only.

57. Partial femur, partial astragalus, partial calcaneum, complete foot bones.

58. (= SIAM 62, SAM P22533). Almost complete individual: the following measurements were added from AMNH 117496. P7, P18, P19. Probably a male (Webb and Taylor, 1995).

59. Femur only.

61. Composite specimen. Partial pelvis, tibia, astragalus, partial calcaneum, and foot bones.

62. Complete except for pelvis (which is partially known, and used to contribute to measurements of AMNH 140809). Probably a female (Webb and Taylor, 1995).

61. No pelvis, otherwise complete. Probably a female (Webb and Taylor, 1995).

62. Pes.

63. Partial pelvis, femur, partial tibia and calcaneum.

64. Astragalus and foot bones.

65. Femur only.
