# Supplementary material for: Locomotion in Extinct Giant Kangaroos: Were Sthenurines Hop-Less Monsters?
Source: PLoS One. 2014 Oct 15;9(10):e109888. doi: 10.1371/journal.pone.0109888 (PMC4198187; doi:10.1371/journal.pone.0109888)
Supplement: Table S4 — Specimens measured for calcaneum only analyses. (DOC) [file pone.0109888.s009.doc]

**Table S4 Specimens measured for calcaneum only analyses,**

| FAMILY | SUBFAMILY | TAXON | SPECIMEN NO. |
| --- | --- | --- | --- |
| Balbaridae✝ |  | *Nambaroo gillespeiae*✝ | QM F35432 |
| Hypsiprymnodontidae |  | *Hypsiprymnodon moschatus* | SAM M11940 |
| Macropodidae | Potoroinae | *Aepyprymnus rufescens* | AMNH 22788 |
| Macropodidae | Potoroinae | *Aepyprymnus rufescens* | QM J5580 |
| Macropodidae | Potoroinae | *Bettongia giamardi cuniculus* | AMNH 65268 |
| Macropodidae | Potoroinae | *Bettongia penicillata* | SAM: M22661 |
| Macropodidae | Potoroinae | *Potorous tridactylus* | SAM M7361 |
| Macropodidae | Potoroinae | *Potorous tridactylus* | AMNH 63509 |
| Macropodidae | incertae sedis | *Ngamaroo archeri*✝ | SAM P23821 |
| Macropodidae | Lagostrophinae | *Lagostrophus fasciatus* | AM M40303 |
| Maczropodidae | Macropodinae | *Dendrolagus benettianus* | WAM M5530 |
| Macropodidae | Macropodinae | *Dendrolagus dorianus* | AM M9109 |
| Macropodidae | Macropodinae | *Dendrolagus lumholtzi* | AMNH 65258 |
| Macropodidae | Macropodinae | *Dendrolagus lumholzi* | AMNH 65263 |
| Macropodidae | Macropodinae | *Dendrolagus matschiei* | AMNH 194793 |
| Macropodidae | Macropodinae | *Dendrolagus scotti* | AM M24424 |
| Macropodidae | Macropodinae | *Dorcopsis luctuosa* | SAM M15178 |
| Macropodidae | Macropodinae | *Dorcopsis muelleri* | AM M32341 |
| Macropodidae | Macropodinae | *Dorcopsulus vanheurni* | AMNH 194790 |
| Macropodidae | Macropodinae | *Lagorchestes conspicillatus* | AMNH 197695 |
| Macropodidae | Macropodinae | *Lagorchestes hirsutus* | AM M40038 |
| Macropodidae | Macropodinae | *Macropus agilis* | AMNH 184582 |
| Macropodidae | Macropodinae | *Macropus agilis* | AMNH 35621 |
| Macropodidae | Macropodinae | *Macropus eugenii* | AMNH 193974 |
| Macropodidae | Macropodinae | *Macropus fuliginosus* | AMNH 200826 |
| Macropodidae | Macropodinae | *Macropus giganteus* | AMNH 35747 |
| Macropodidae | Macropodinae | *Macropus giganteus* | QM J11525 |
| Macropodidae | Macropodinae | *Macropus giganteus* | AMNH 90136 |
| Macropodidae | Macropodinae | *Macropus giganteus* | AMNH 42904 |
| Macropodidae | Macropodinae | *Macropus irma* | AMNH 150319 |
| Macropodidae | Macropodinae | *Macropus robustus* | AMNH 65036 |
| Macropodidae | Macropodinae | *Macropus rufogriseus* | AMNH 14154 |
| Macropodidae | Macropodinae | *Macropus rufogriseus* | AMNH 273247 |
| Macropodidae | Macropodinae | *Macropus rufus* | AMNH 200473 |
| Macropodidae | Macropodinae | *Macropus rufus* | AMNH 70284 |
| Macropodidae | Macropodinae | *Macropus rufus* | QM J22115 |
| Macropodidae | Macropodinae | *Macropus* cf *ferragus*✝ | SAM P43039 |
| Macropodidae | Macropodinae | *Onychogalea fraenata* | AMNH 42959 |
| Macropodidae | Macropodinae | *Onychogalea fraenata* | NMV C6500 |
| Macropodidae | Macropodinae | *Petrogale assimilis* | QM J4470 |
| Macropodidae | Macropodinae | *Petrogale lateralis* | AM 24183 |
| Macropodidae | Macropodinae | *Petrogale penicillata* | AMNH 35758 |
| Macropodidae | Macropodinae | *Setonix brachyurus* | AMNH 196399 |
| Macropodidae | Macropodinae | *Thylogale billardierii* | AMNH 65215 |
| Macropodidae | Macropodinae | *Thylogale stigmatica* | AMNH 65140 |
| Macropodidae | Macropodinae | *Thylogale thetis* | AM M51512 |
| Macropodidae | Macropodinae | *Wallabia bicolor* | AMNH 65722 |
| Macropodidae | Macropodinae | *Dorcopsoides*✝ sp. | NT P890 |
| Macropodidae | Macropodinae | *Dorcopsoides*✝ sp. | NT P648-7 |
| Macropodidae | Macropodinae | *Dorcopsoides*✝ sp. | NT P678-6 |
| Macropodidae | Macropodinae | *Dorcopsoides*✝ sp. | NT unnumbered |
| Macropodidae | Macropodinae | *Protemnodon anak*✝ | QM F14675 |
| Macropodidae | Macropodinae | *Protemnodon brehus*✝ | SAM P20810 |
| Macropodidae | Macropodinae | *Protemnodon snewini*✝ | QM F9075/9076 |
| Macropodidae | Macropodinae | *Protemnodon*✝ sp. (small) | AMNH/SIAM 37 |
| Macropodidae | Macropodinae | *Protemnodon*✝ sp. (large) | AMNH/SIAM 37 |
| Macropodidae | Macropodinae | *Protemnodon*✝ sp. | FU 1661 |
| Macropodidae | Macropodinae | *Protemnodon*✝ sp. | FU CN40-01-19 |
| Macropodidae | Sthenurinae✝ | *Archaeosimus cegsai*✝ | FU unnumbered |
| Macropodidae | Sthenurinae✝ | *Hadronomas puckridgei*✝ | NT MPUD-07/72 |
| Macropodidae | Sthenurinae✝ | *Hadronomas puckridgei*✝ | NT P9336 |
| Macropodidae | Sthenurinae✝ | *Hadronomas puckridgei*✝ | NT P8745-2 |
| Macropodidae | Sthenurinae✝ | *Rhizosthenurus flanneryi*✝ | QM F31456 |
| Macropodidae | Sthenurinae✝ | *Procoptodon*✝ sp. | AMNH 91272 |
| Macropodidae | Sthenurinae✝ | *Procoptodon*✝ sp. | SAM unnumbered |
| Macropodidae | Sthenurinae✝ | *Simosthenurus occidentalis*✝ | SAM P20820 |
| Macropodidae | Sthenurinae✝ | *Sthenurus stirlingi*✝ | AMNH 117496 |
| Macropodidae | Sthenurinae✝ | *Sthenurus stirlingi*✝ | AMNH 117497 |
| Macropodidae | Sthenurinae✝ | *Sthenurus stirlingi*✝ | AMNH 117494 |
| Macropodidae | Sthenurinae✝ | *Sthenurus tindalei*✝ | AMNH 117493 |

✝ = extinct taxon.

Acronyms for museums:

AM = Australian Museum. AMNH = American Museum of Natural History. FU = Flinders University. QM = Queensland Museum. NMV = National Museum Victoria. NT = Northern Territories Museum. SAM/SIAM= South Australian Museum. WAM = Western Australian Museum.
