# Supplementary material for: Locomotion in Extinct Giant Kangaroos: Were Sthenurines Hop-Less Monsters?
Source: PLoS One. 2014 Oct 15;9(10):e109888. doi: 10.1371/journal.pone.0109888 (PMC4198187; doi:10.1371/journal.pone.0109888)
Supplement: Table S5 — Data for all of the bones. (PDF) [file pone.0109888.s010.pdf]

TABLE S5. DATA FOR ALL OF THE BONES

FAMILY	SUBFAMILY	GENUS		SPECIES
Balbaridae		Nambaroo		gillespieae
Hypsiprymnodontidae		Hypsiprymnodon	1	moschatus
Macropodidae	incertae sedis	Ngamaroo		archeri
Macropodidae	Potoroinae	Aepyprymnus	1	rufescens
Macropodidae	Potoroinae	Aepyprymnus	1	rufescens
Macropodidae	Potoroinae	Caloprymnus	1	campestris
Macropodidae	Potoroinae	Bettongia	1	leseur
Macropodidae	Potoroinae	Bettongia	1	tropica
Macropodidae	Potoroinae	Bettongia	1	penicillata
Macropodidae	Potoroinae	Potorous	1	longipes
Macropodidae	Potoroinae	Potorous	1	tridactylus
Macropodidae	Lagostrophinae	Lagostrophus	1	fasciatus
Macropodidae	Lagostrophinae	Lagostrophus	1	fasciatus
Macropodidae	Lagostrophinae	Lagostrophus	1	fasciatus
Macropodidae	Macropodinae	Dendrolagus	1	bennettianus
Macropodidae	Macropodinae	Dendrolagus	1	dorianus
Macropodidae	Macropodinae	Dendrolagus	1	dorianus
Macropodidae	Macropodinae	Dendrolagus	1	goodfellowi
Macropodidae	Macropodinae	Dendrolagus	1	lumholtzi
Macropodidae	Macropodinae	Dendrolagus	1	lumholtzi
Macropodidae	Macropodinae	Dendrolagus	1	matschiei
Macropodidae	Macropodinae	Dendrolagus	1	matschei
Macropodidae	Macropodinae	Dendrolagus	1	scottae
Macropodidae	Macropodinae	Dorcopsis	1	atrata
Macropodidae	Macropodinae	Dorcopsis	1	luctuosa
Macropodidae	Macropodinae	Dorcopsis	1	muelleri
Macropodidae	Macropodinae	Dorcopsis	1	muelleri
Macropodidae	Macropodinae	Dorcopsis	1	veterum
Macropodidae	Macropodinae	Dorcopsulus	1	vanheurni
Macropodidae	Macropodinae	Lagorchestes	1	hirsutus
Macropodidae	Macropodinae	Lagorchestes	1	hirsutus
Macropodidae	Macropodinae	Lagorchestes	1	conspicillatus
Macropodidae	Macropodinae	Lagorchestes	1	conspicillatus
Macropodidae	Macropodinae	Macropus	1	agilis
Macropodidae	Macropodinae	Macropus	1	antilopinus
Macropodidae	Macropodinae	Macropus	1	dorsalis
Macropodidae	Macropodinae	Macropus	1	eugenii
Macropodidae	Macropodinae	Macropus	1	fuliginosus
Macropodidae	Macropodinae	Macropus	1	fuliginosus
Macropodidae	Macropodinae	Macropus	1	giganteus
Macropodidae	Macropodinae	Macropus	1	giganteus
Macropodidae	Macropodinae	Macropus	1	giganteus
Macropodidae	Macropodinae	Macropus	1	parma
Macropodidae	Macropodinae	Macropus	1	parryi
Macropodidae	Macropodinae	Macropus	1	robustus

Macropodidae	Macropodinae	Macropus	1	robustus
Macropodidae	Macropodinae	Macropus	1	rufogriseus
Macropodidae	Macropodinae	Macropus	1	rufus
Macropodidae	Macropodinae	Macropus	1	rufus
Macropodidae	Macropodinae	Macropus	1	rufus
Macropodidae	Macropodinae	Macropus	1	rufus
Macropodidae	Macropodinae	Macropus		ferragus
Macropodidae	Macropodinae	Macropus		ferragus
Macropodidae	Macropodinae	Macropus		cf ferragus
Macropodidae	Macropodinae	Macropus		cf ferragus
Macropodidae	Macropodinae	Macropus		titan
Macropodidae	Macropodinae	Macropus		titan
Macropodidae	Macropodinae	Onychoglea	1	fraenata
Macropodidae	Macropodinae	Onychoglea	1	fraenata
Macropodidae	Macropodinae	Onychogalea	1	unguifera
Macropodidae	Macropodinae	Petrogale	1	assimilis
Macropodidae	Macropodinae	Peradorcas	1	concinna
Macropodidae	Macropodinae	Peradorcas	1	concinna
Macropodidae	Macropodinae	Petrogale	1	lateralis
Macropodidae	Macropodinae	Petrogale	1	penicillata
Macropodidae	Macropodinae	Petrogale	1	xanthropus
Macropodidae	Macropodinae	Petrogale	1	xanthropus
Macropodidae	Macropodinae	Setonix	1	brachyurus
Macropodidae	Macropodinae	Setonix	1	brachyurus
Macropodidae	Macropodinae	Thylogale	1	billardierii
Macropodidae	Macropodinae	Thylogale	1	billardierii
Macropodidae	Macropodinae	Thylogale	1	stigmatica
Macropodidae	Macropodinae	Thylogale	1	thetis
Macropodidae	Macropodinae	Wallabia	1	bicolor
Macropodidae	Macropodinae	Dorcopsoides		sp.
Macropodidae	Macropodinae	Protemnodon		anak
Macropodidae	Macropodinae	Protemnodon	66	anak
Macropodidae	Macropodinae	Protemnodon		snewini
Macropodidae	Macropodinae	Protemnodon		sp.
Macropodidae	Sthenurinae	Hadronomas		puckridgei
Macropodidae	Sthenurinae	Hadronomas		puckridgei
Macropodidae	Sthenurinae	Hadronomas		puckridgei
Macropodidae	Sthenurinae	Hadronomas		puckridgei
Macropodidae	Sthenurinae	Hadronomas		puckridgei
Macropodidae	Sthenurinae	Hadronomas		puckridgei
Macropodidae	Sthenurinae	Hadronomas		puckridgei
Macropodidae	Sthenurinae	Hadronomas		puckridgei
Macropodidae	Sthenurinae	Hadronomas		puckridgei
Macropodidae	Sthenurinae	Hadronomas		puckridgei
Macropodidae	Sthenurinae	Hadronomas		puckridgei
Macropodidae	Sthenurinae	Hadronomas		puckridgei
Macropodidae	Sthenurinae	Hadronomas		puckridgei

Macropodidae	Sthenurinae	Hadronomas	puckridgei
Macropodidae	Sthenurinae	Hadronomas	puckridgei
Macropodidae	Sthenurinae	Hadronomas	puckridgei
Macropodidae	Sthenurinae	Hadronomas	puckridgei
Macropodidae	Sthenurinae	Hadronomas	puckridgei
Macropodidae	Sthenurinae	Hadronomas	puckridgei
Macropodidae	Sthenurinae	Hadronomas	puckridgei
Macropodidae	Sthenurinae	Hadronomas	puckridgei
Macropodidae	Sthenurinae	Hadronomas	puckridgei
Macropodidae	Sthenurinae	Hadronomas	puckridgei
Macropodidae	Sthenurinae	Rhizosthenurus	flanneryi
Macropodidae	Sthenurinae	Procoptodon	sp.
Macropodidae	Sthenurinae	Procoptodon	goliah
Macropodidae	Sthenurinae	"Procoptodon"	browneorum
Macropodidae	Sthenurinae	"Procoptodon"	browneorum
Macropodidae	Sthenurinae	"Procoptodon"	gilli
Macropodidae	Sthenurinae	"Procoptodon"	gilli
Macropodidae	Sthenurinae	"Procoptodon"	gilli
Macropodidae	Sthenurinae	"Procoptodon"	gilli
Macropodidae	Sthenurinae	"Procoptodon"	gilli
Macropodidae	Sthenurinae	"Procoptodon"	gilli
Macropodidae	Sthenurinae	Simosthenurus	maddocki
Macropodidae	Sthenurinae	Simosthenurus	occidentalis
Macropodidae	Sthenurinae	Simosthenurus	occidentalis
Macropodidae	Sthenurinae	Simosthenurus	occidentalis
Macropodidae	Sthenurinae	Simosthenurus	occidentalis
Macropodidae	Sthenurinae	Simosthenurus	occidentalis
Macropodidae	Sthenurinae	Simosthenurus	occidentalis
Macropodidae	Sthenurinae	Simosthenurus	occidentalis
Macropodidae	Sthenurinae	Simosthenurus	occidentalis
Macropodidae	Sthenurinae	Simosthenurus	occidentalis
Macropodidae	Sthenurinae	Simosthenurus	occidentalis
Macropodidae	Sthenurinae	Simosthenurus	occidentalis
Macropodidae	Sthenurinae	Simosthenurus	occidentalis
Macropodidae	Sthenurinae	Simosthenurus	occidentalis
Macropodidae	Sthenurinae	Simosthenurus	occidentalis
Macropodidae	Sthenurinae	Simosthenurus	occidentalis
Macropodidae	Sthenurinae	Simosthenurus	occidentalis
Macropodidae	Sthenurinae	Simosthenurus	occidentalis
Macropodidae	Sthenurinae	Simosthenurus	occidentalis
Macropodidae	Sthenurinae	Simosthenurus	occidentalis
Macropodidae	Sthenurinae	Simosthenurus	occidentalis
Macropodidae	Sthenurinae	Sthenurus	andersoni
Macropodidae	Sthenurinae	Sthenurus	stirlingi
Macropodidae	Sthenurinae	Sthenurus	stirlingi
Macropodidae	Sthenurinae	Sthenurus	stirlingi
Macropodidae	Sthenurinae	Sthenurus	stirlingi
Macropodidae	Sthenurinae	Sthenurus	stirlingi
Macropodidae	Sthenurinae	Sthenurus	stirlingi

Macropodidae	Sthenurinae	Sthenurus		tindalei
Macropodidae	Sthenurinae	Sthenurus	1	tindalei
Macropodidae	Sthenurinae	Sthenurus	1	sp.

SPECIMEN NUMBER	P1	P2	P3	P4	P5	P6	P7
QM F35432	84.86	18.62	15.85	21.59	22.87	43.48	48.43
SAM M11940	31.66	6.43	5.15	13.01	7.46	20.99	17.73
SAM P23821	.	.	.	.	.	.	.
AMNH 65283	48.59	10.3	9.45	25.21	17.49	30.94	31.24
QM J5579	48.99	9.43	8.48	25.73	15.56	38.43	32.51
NT 48050	32.62	7.53	7.3	20.83	15.5	25.55	20.34
AM M2132	43.02	10.36	8.58	24.14	16.32	33.32	29.17
AM M40067	38.86	7.35	7.41	22.14	15.51	27.86	28.46
SAM M18986	33.81	8.09	7.25	19.71	12.95	28.09	24.38
NMV C32723	45.04	10.92	11.54	20.38	14.98	32.81	31.21
AMNH 65297	43.01	7.03	9.36	19.46	14.9	26.28	29.05
WAM M16285	45.02	10.57	7.41	24.95	16	37.05	29
WAM M4393	41.35	10.18	7.57	22.21	14.95	33.52	25.78
AM M40303	45.74	10.87	7.67	24.42	19.67	40.49	35.15
WAM M5530	84.15	17.48	16.74	43.26	28.11	46.54	43.74
WAM M7643	84.24	14.66	12.76	35.66	22.71	34.71	34.15
AM M9109	69.39	14.96	14.97	39.63	27.7	33.62	36.13
NMV C.25092	63.93	13.78	14.06	35.9	34.93	31.49	34.16
AMNH 65265	73.78	13.75	12.88	40.09	21.51	37.27	34.33
SAM M7206	76.18	19.07	14.07	41.87	30.95	37.33	36.67
WAM M21013	72.81	18.42	15.84	38.99	25.9	34.19	36.38
QM J5287	78.03	16.55	17.78	42.38	30.08	45.04	38.24
AM M24424	.	.	.	.	.	.	.
AM M19461	79.08	13.51	16.73	.	.	.	50.11
SAM M15178	65.78	18.14	14.38	34.46	21.06	42.71	44.78
AM M32339	76.02	13.03	13.7	36.74	25.19	43.52	45.54
AM M32341	95.18	20.86	17.47	.	.	.	64.58
AMNH 22262	75.25	13.71	12.23	39.42	24.14	37.65	48.43
AMNH 194790	54.53	8.88	10.04	25.6	21.25	30.28	27.36
SAM M3587	44.18	9.48	7.02	25.73	16	34.09	28.47
AM M40038	41.66	10.97	7.75	23.62	17.4	35.88	30.75
WAM M7032	54.93	11.37	8.56	27.36	19.81	34.99	33.02
AMNH 197659	54.1	10.33	8.88	31.02	21.2	30.16	31.01
AMNH 35750	92.67	18.3	17	45.9	32.47	75.45	58.01
AMNH 70449	129.83	27.71	28.1	65.11	44.65	95.85	84.27
NMV C6490	74.92	16.1	13.53	41.27	29.32	48.24	50.72
NMV C7908	64.82	13.24	11.89	34.56	20.66	51.28	41.71
SAM M21497	166.66	37.02	32.28	96.15	77.89	136.91	115.24
SAM M16578	124.9	30.03	22.3	62.9	50.23	104.27	85.24
AMNH 35747	160.33	33.91	32.28	82.71	63.35	121.56	106.87
AMNH 35186	127.44	28.52	25.19	65.1	52.18	99.63	91.21
QM J11525	147	32.56	29.53	86.76	71.72	122.69	105.1
SAM M7192	56.95	13.35	9.95	28.12	21.6	39.46	35.88
AMNH 65054	89.27	19.51	16.99	47.82	32.91	69.65	55.14
SAM M3695	127.97	29.64	28.66	68.1	46.07	108.54	94.75

AMNH 65036	129.1	29.15	27.55	62.32	46.24	97.68	83.43
AMNH 65116	85.6	20.84	18.33	44.43	30.88	67.29	56.79
SAM M6559	121.15	27.43	19.79	58.94	45.84	110.83	81.19
AMNH 200473	135.73	30.13	23.95	68.41	48.89	87.75	91.64
AMNH 70284	169.37	34.5	33.47	85.47	66.73	145.83	114.22
QM J22115	143.98	33.04	35.05	73.31	57.3	127.79	97.96
NMV unnumbered	.	.	.	.	.	.	.
NMV unnumbered	.	.	.	.	.	.	.
SAM P194676	.	.	.	.	.	.	.
SAM P43039	.	.	.	.	.	.	.
AMNH 18362	214.99	43.96	41.23	.	.	.	.
NMV Lancefield composite	.	.	.	.	.	.	.
NMV C6400	56.98	13.17	10.35	29.23	20.4	53.79	50.37
AMNH 42959	48.2	11.21	9.03	24.59	20.92	46.14	42.17
WAM M11622	63.08	13.68	11.51	34.37	21.5	60.94	47.97
QM J4470	60	13.86	11.18	32.59	21.39	37.96	40.25
WAM M9360	34.4	7.18	6.43	19.24	14.89	23.01	23.16
NMV C6478	41.76	9.64	7.99	22.11	16.41	21.3	24.62
AM M24183	71.89	13	10.96	32.3	22.88	33.82	37.57
AMNH 65241	64.08	11.99	10.78	34.81	25.5	33.82	53.97
AMNH 35642	55.59	10.97	11.32	30.77	25.15	35.59	36.28
WAM M11469	78.91	18.5	12.9	38.8	26.23	48.64	50.15
WAM M6792	57.29	14.67	11.91	31.67	22.06	32.5	38.76
NMV C23029	58.85	15.9	12.63	31.28	19.97	34.6	39.06
NMV C11311	71.25	13.58	11.16	38.96	27.82	39.1	49.75
NMV C11326	86.2	18.09	17.55	46.65	29.87	32.47	58.95
AMNH 65153	71.8	12.47	10.31	38.33	27.55	28.2	48.43
AM M51512	53.53	11.89	10.22	30.73	19.69	35.54	33.18
AMNH 65722	90.96	19.99	15.76	47.54	42.07	63.27	58.04
NT P890	65.2	17.81	14.91	.	.	.	34.74
NMV unnumbered composite	.	.	.	.	.	.	.
QM F14675	.	.	.	.	.	.	.
QM F9075/9076	.	.	.	.	.	.	.
AMNH 117494	.	.	.	.	.	.	.
NT MPA61	.	.	.	.	.	.	.
NT P98141	.	.	.	.	.	.	.
NT MP1279	.	.	.	.	.	.	.
NT MP1200	.	.	.	.	.	.	.
NT unnumbered	.	.	.	.	.	.	.
NT GB5-5	.	.	.	.	.	.	.
NT P-2911dW	.	.	.	.	.	.	.
NT MPUD-07/72	.	.	.	.	.	.	.
NT P9336	.	.	.	.	.	.	.
NT SP69	.	.	.	.	.	.	.
NT A122	.	.	.	.	.	.	.
NT unnumbered	.	.	.	.	.	.	.
NT P9214	.	.	.	.	.	.	.

NT P87-1422	.	.	.	.	.	.	.
NT SP708	.	.	.	.	.	.	.
NT SP488	.	.	.	.	.	.	.
NT P9260	.	.	.	.	.	.	.
NT MP162-4	.	.	.	.	.	.	.
NT P9261	.	.	.	.	.	.	.
NT MP328	.	.	.	.	.	.	.
NT unnumbered	.	.	.	.	.	.	.
NT P877-15	.	.	.	.	.	.	.
NT P877-18	.	.	.	.	.	.	.
QM F31456	.	.	.	.	.	.	.
NMV unnumbered							
NMV unnumbered							
WAM 65-4-78	.	.	.	.	.	.	.
WAM 68-3-637	.	.	.	.	.	.	.
SAM P17469	131.4	40.76	39.81	86.27	61.66	55.48	76.26
SAM P17277	.	.	.	.	.	.	.
SAM unnumbered	.	.	.	.	.	.	.
SAM P17291	.	.	.	.	.	.	.
SAM unnumbered	.	.	.	.	.	.	.
SAM P17528	.	.	.	.	.	.	.
SAM P1A1	.	.	.	.	.	.	.
SAM P20820	182.62	54.28	40.54	109.07	87.67	59.13	85.77
SAM P17258	171.56	48.96	36.19	106.42	73.82	62.15	96.97
SAM P17472	191.56	55.05	35.77	123.25	89.58	65.58	101.52
SAM P17474	.	.	.	.	.	.	.
SAM P18298	.	.	.	.	.	.	.
SAM unnumbered	.	.	.	.	.	.	.
SAM P17474.1	.	.	.	.	.	.	.
SAM P17262	.	.	.	.	.	.	.
SAM P17296	.	.	.	.	.	.	.
SAM P17475	.	.	.	.	.	.	.
SAM P17260	.	.	.	.	.	.	.
WAM 65-4-133	.	.	.	.	.	.	.
WAM unnumbered	.	.	.	.	.	.	.
SAM P40068	.	.	.	.	.	.	.
SAM P18296	.	.	.	.	.	.	.
WAM 65-4-131	.	.	.	.	.	.	.
WAM 65-4-62	.	.	.	.	.	.	.
WAM 65-4-64	.	.	.	.	.	.	.
WAM 65-4-66	.	.	.	.	.	.	.
WAM 65-4-68	.	.	.	.	.	.	.
SAM P13673	.	.	.	.	.	.	.
AMNH 140809	243.21	49.59	41.66	102.19	76.81	93.48	124
SAM P17259	.	.	.	.	.	.	.
NMV unnumbered composite	.	.	.	.	.	.	.
AMNH 117496	.	52.65	41.18	95.18	43.19	71.3	124.1
AMNH 117497	.	.	.	.	.	.	.
AMNH 117494A	.	.	.	.	.	.	.

AMNH 117499	.	.	.	.	.	.	.
AMNH 117493	184.05	36.97	.	.	.	.	.
AMNH unnumbered	.	.	.	.	.	.	.

P8	P9	P10	P11	P12	P13	P14	P15	P16
68.66	21.17	63.3	18.01	23.31	27.5	24.54	63.08	10.38
29.33	7.12	23.54	9.74	7.17	8.8	8.69	17.27	3.06
.	.	.	.	.	.	.	.	.
47.17	9.26	40.84	12.37	8.09	9.21	15.45	24.72	5.76
48.61	9.37	43.33	11.94	10.54	12.79	16.16	20.07	5.59
32.3	8.11	28.83	8.96	8.6	10.67	7.65	28.53	6.09
41.02	9.05	37.09	12.51	10.66	13.72	11.78	22.11	7.44
36.86	9.2	33.36	12.24	6.92	9.82	10.81	19.73	4.49
31.37	7.51	30.3	10.96	7.5	9.91	12.26	18.41	3.75
42.2	9.25	36.25	10.18	8.45	9.16	11.88	20.98	6.11
42.13	7.89	35.56	10.06	5.9	6.72	12.16	16.81	4.99
42.92	9.47	39.06	14.01	12.16	12.7	10.25	32	6.5
40.38	8.53	34.33	10.79	8.95	10.86	10.88	32.76	6.87
44.93	9.47	39.6	11.89	10.34	11.53	13.62	32.17	7.08
83.54	16.16	73.51	22.05	20.9	27.26	24.37	64.55	10.01
65.25	14.73	60.31	18.81	17	24.29	21.7	48.76	15.88
72.27	16.49	59.81	22.36	19.68	23.4	23.27	59.02	13.07
67.6	14.31	58.32	14.77	15.7	21.95	21.48	56.76	10.3
72.98	13.34	63.45	17.18	19.39	22.19	23.1	56.77	7.75
69.79	18.76	65.61	18.92	21.4	24.74	21.04	57.86	6.09
70.8	14.17	60.05	19.19	14.21	17.97	22.09	55.6	12.55
74.65	16.97	69.34	21.26	18.28	22.21	33.46	63.74	10.37
.	.	.	.	.	.	.	.	.
64.5	18.14	70.3	20.64	24.83	29.1	.	.	.
63.24	13.97	58.91	19.48	17.53	19.52	20.37	55.86	11.49
71.04	13.93	60.44	18.42	15.15	17.44	25.15	63.08	11.16
90.94	18.26	80.4	25.88	22.86	25.8	32.31	.	.
64.05	16.18	63.3	17.6	17.54	20.8	24.24	63.08	11.37
52.29	8.52	42.59	12.42	9.46	14.5	13.79	46.49	8.15
41.44	7.4	37.16	10.4	9.42	11.84	12.4	36.68	7.75
40.82	9.23	34.73	11.67	11.56	12.48	9.35	36.09	7.79
50.83	7.71	47.59	9.11	9.61	13.51	12.48	38.88	7.18
45.65	8.03	46.04	12.59	8.56	10.43	14.65	33.41	10.54
86.87	17.49	87.55	20.1	14.61	17.52	24.06	75.57	8.9
123.72	29.02	123.28	33.13	25.58	31.26	35.53	87.66	18.81
72.93	14.23	64.98	20.23	16.67	20.85	24.01	49.72	7.01
59.62	12.78	54.89	16.76	11.39	13.33	20.59	45.22	8.36
162.34	33.32	159.98	39.88	33.14	36.3	43.11	68.37	11.48
118.58	27.92	114.54	34.31	21.71	25.74	37.12	73.76	8.54
151.1	34.02	156.54	41.15	32.25	35.65	46.21	77.82	16.83
129.99	25.52	118.83	29.92	19.71	23.32	38.17	84.7	10.86
141.08	34.84	141.44	40.36	28.47	31.31	52.46	64.07	16.39
53.05	12.17	45.32	13.65	10.55	11.93	13.62	36.22	6.96
83.99	18.85	79.3	22.63	15.77	18.91	22.43	66.84	8.45
132.53	29.89	130.28	36.13	28.06	20.03	34.79	83.98	23.07

133.44	29	122.96	36.24	24.4	29.27	34.25	74.26	16.6
81.66	17.95	79.64	20.53	17.22	20.07	24.52	63.76	10.17
112.6	27.73	117.11	35.02	20.32	24.13	33.23	75.02	12.08
131.01	32.84	130.31	39.33	30.02	33.89	41.32	80	15
160.12	38.73	158.64	51.15	34.4	35.92	59.9	89.53	20.68
138.61	30.29	139.96	43.04	30.15	31.48	47.85	70.55	12.7
.	.	.	.	.	.	.	.	.
.	.	.	.	.	.	.	.	.
.	.	.	.	.	.	.	.	.
.	.	.	.	.	.	.	.	.
203.41	49.73	205.53	59.24	45.87	57.9	66.52		
.	.	.	.	.	.	.	.	.
57.28	12.15	53.23	13.37	9.98	10.38	18.45	27.25	4.33
47.05	10.9	43.66	13.34	9.46	12.75	14	19.45	3.41
58.02	14.11	57.45	18.14	12.51	15.24	20.65	34.11	7.77
58.37	12.3	52.03	14.7	12.32	14.18	19.76	43.44	4.7
34.4	7.94	29.72	9.3	7.35	8.9	9.69	30.64	4.32
40.35	9.84	36.97	11.02	6.27	6.42	9.96	31.33	5.18
54.95	.	48.54	.	9.98	11.41	15.01	46.36	7.7
57.71	14.33	56.33	16.82	13.46	19.36	23.49	33.62	8.99
56.68	11.47	46.64	13.07	10.23	12.12	12.64	43.44	4.7
75.62	16.97	67.55	18.74	15.62	19.77	19.91	51.02	7.21
57.07	10.18	49.14	13.8	13.41	18.6	15.76	40.32	6.88
61.94	10.57	50.98	13.02	12.06	11.47	17.26	40.42	7.93
68.71	15.88	61.67	17.58	10.89	11.43	21.89	48.02	9.18
84.7	16.57	83.69	20.95	17.03	19.7	32.27	74.33	11.01
71.29	14.75	60.34	17.35	14.04	18.16	20.11	45.02	6.98
52.51	11.9	.	15.04	11.74	17.38	17.68	34.16	7.79
88.04	18.59	80.16	21.24	20.3	23.03	18.51	77.55	12.82
61.1	.	55.5	.	.	.	.	.	.
.	.	.	.	.	.	.	.	.
.	.	.	.	.	.	.	.	.
.	.	.	.	.	.	.	.	.
.	.	.	.	.	.	.	.	.
.	.	.	.	.	.	.	.	.
.	.	.	.	.	.	.	.	.
.	.	.	.	.	.	.	.	.
.	.	.	.	.	.	.	.	.
.	.	.	.	.	.	.	.	.
.	.	.	.	.	.	.	.	.
.	.	.	.	.	.	.	.	.
.	.	.	.	.	.	.	.	.
.	.	.	.	.	.	.	.	.
.	.	.	.	.	.	.	.	.
.	.	.	.	.	.	.	.	.
.	.	.	.	.	.	.	.	.
.	.	.	.	.	.	.	.	.

.	.	.	.	.	.	.	.	.
.	.	.	.	.	.	.	.	.
.	.	.	.	.	.	.	.	.
.	.	.	.	.	.	.	.	.
.	.	.	.	.	.	.	.	.
.	.	.	.	.	.	.	.	.
.	.	.	.	.	.	.	.	.
.	.	.	.	.	.	.	.	.
.	.	.	.	.	.	.	.	.
.	.	.	.	.	.	.	.	.
.	.	.	.	.	.	.	.	.
.	.	.	.	.	.	.	.	.
.	.	.	.	.	.	.	.	.
126.81	32.61	117.65	38.57	39.01	47.18	41.06	111	14.5
.	.	.	.	.	.	.	.	.
.	.	.	.	.	.	.	.	.
.	.	.	.	.	.	.	.	.
.	.	.	.	.	.	.	.	.
.	.	.	.	.	.	.	.	.
.	.	.	.	.	.	.	.	.
175.36	39.3	172.25	53.9	53.45	65.05	69.93	159.21	22.19
166.16	49.42	163.83	60.44	55.88	62.48	49.03	161.89	23.71
194.11	47.46	178.55	56.86	58.78	72.29	49.71	168.41	22.73
.	.	.	.	.	.	.	.	.
.	.	.	.	.	.	.	.	.
.	.	.	.	.	.	.	.	.
.	.	.	.	.	.	.	.	.
.	.	.	.	.	.	.	.	.
.	.	.	.	.	.	.	.	.
.	.	.	.	.	.	.	.	.
.	.	.	.	.	.	.	.	.
.	.	.	.	.	.	.	.	.
.	.	.	.	.	.	.	.	.
.	.	.	.	.	.	.	.	.
.	.	.	.	.	.	.	.	.
.	.	.	.	.	.	.	.	.
.	.	.	.	.	.	.	.	.
.	.	.	.	.	.	.	.	.
.	.	.	.	.	.	.	.	.
.	.	.	.	.	.	.	.	.
.	.	.	.	.	.	.	.	.
240.82	50.85	225.75	60.42	65.55	83.64	62.53	188.26	32.55
.	.	.	.	.	.	.	.	.
.	.	.	.	.	.	.	.	.
	58.41					53.54	193.65	30.17
.	.	.	.	.	.	.	.	.
.	.	.	.	.	.	.	.	.

P17	P18	P19	P20	P21	P22	F1	F4	F5
5.94	9.13	8.67	38.65	25.46	55.55	133.85	12.94	20.78
1.32	3.82	3.22	14.8	9.68	19.36	60.59	4.725	5.61
.	.	.	.	.	.	107.29	10.665	.
4.33	5.12	8.88	32.23	18.28	45.48	94.12	8.19	13.94
4.77	4.41	8.21	31.91	18.04	32.95	96.65	8.215	12.65
2.89	5.83	5.16	19.04	15.79	29.92	87.39	6.425	9.14
4.83	5.81	5.62	27.73	17.9	37.2	78.81	7.4	11.23
2.36	4.49	6.31	24.64	15.67	28.31	80.31	6.89	10.14
4.77	4.92	4.52	23.06	13.13	30.83	72.58	6.485	11.22
3.94	4.64	7.38	28.09	16.89	31.58	85.04	7.545	12.4
3.48	5.61	5.51	23	15.39	31.14	78.1	6.39	8.48
4.31	5.97	4.15	26.1	17.1	31.8	85.14	7.315	10.75
4.68	4.34	3.07	26.34	13.84	27.77	80.48	6.835	10.03
3.91	4.55	5.94	27.19	15.85	30.03	86.38	6.875	13.27
5.73	8.25	5.23	42.68	27.23	51.33	143.66	14.99	25.87
2.49	8.15	6.32	30.15	22.96	51.38	120.49	11.385	19
2.92	8.26	13.72	26.57	22.61	55.94	124.75	12.41	20.42
4.07	7.34	9.34	31.92	19.93	48.79	122.54	11.505	15.11
4.08	7.18	9.62	31.2	19.18	50.73	124.23	12.765	21.79
2.5	6.82	9.62	33.29	18.15	50.57	126.16	13.18	15.6
3.99	8.94	6.51	28.94	20.43	53.08	128.53	13.44	22.18
4.02	9.58	16.96	34.5	22.72	47.73	137.9	15.43	20.16
.	.	.	.	.	.	140.91	12.81	20.66
.	9.95	11.25	.	.	.	134.69	12.87	16.22
5.7	8.48	6.3	35.34	24.59	54.65	121.25	11.63	19.53
5.69	8.01	11.43	38.74	23.83	55.7	130.54	11.51	17.54
.	10.46	14.85	.	.	.	162.89	14.465	23.77
6.33	9.13	8.67	36.63	25.73	55.55	133.87	10.89	15.9
6.51	4.6	6.58	25.82	13.77	38.88	101.07	7.77	11.79
5.26	6.08	3.85	25.42	14.33	29.49	91.32	6.99	14.88
4.39	4.68	6.19	28.68	14.81	39.09	80.08	7.615	12.56
3.67	5.6	2.94	31.32	17.88	33.42	104.76	8.785	14.75
5.02	8.78	6.79	32.39	17.17	41.26	104.11	8.885	15.77
7.95	9.39	8.99	46.45	23.97	61.18	162.22	14.43	28.76
13.43	14.65	14.94	65.56	35.25	90.51	221.71	21.13	42.58
7.47	9.55	8.72	41.52	24.97	49.38	134.89	12.06	13.69
6.77	8.81	7.63	35.91	20.94	43.93	114.85	11.29	19.22
13.46	16.37	9.58	104.38	50.91	114.06	246.91	27.83	66.34
12.02	14.82	9.07	83.79	42.54	87.91	211.9	22.255	43.79
12.94	19.67	12.06	98.01	46.86	117.34	254.44	27.43	50.33
10.12	13.46	12.9	77.18	40.87	74.89	212.05	21.35	20.62
13.01	20.01	22.8	93.76	47.4	112.11	252.71	26.5	55.15
4.1	6.11	5.06	30.21	16.82	32.78	110.75	9.51	15.92
11.03	8.14	12.96	44.68	20.09	62.32	168.32	15.045	28.56
14.94	15.51	9.99	73.63	36.67	78.09	235.97	22.83	44.81

16.27	16.29	15.33	73.77	36.12	81.93	232.61	22.085	24.35
9.07	10.6	6.63	48.67	25.8	60.4	156.8	15.27	28.35
9.63	13.09	7.55	71.2	35.58	75.82	213.24	20.54	39.13
11.9	16.13	14.46	83.83	42.17	94.84	223.1	23.89	37.65
17.85	15.99	17.52	92.21	45.96	102.5	279.92	27.575	61.97
9.45	16.4	15.5	88.18	41.23	96.02	237.77	27.715	51.12
.	.	.	.	.	.	.	.	.
.	.	.	.	.	.	.		47.18
.	.	.	.	.	.	.	.	.
.	.	.	.	.	.	.		.
						320	38.73	63.13
.	.	.	.	.	.	.	.	.
9	3.35	6.66	37.6	19.29	37.53	113.4	9.59	14.09
5.45	8.76	9.48	34.68	18.06	34.91	102.35	7.81	11.05
6.35	8.38	6.17	44.72	28.22	52.75	132.36	12.64	23.94
4.64	6.38	6.61	33.91	19.92	41.12	119.13	10.045	16.19
3.66	4.13	3.52	20.33	10.7	24.36	72.62	6.305	10.37
2.7	4.29	4.81	22.1	14.21	26.75	82.98	6.675	8.67
5.19	6.59	6.22	28.33	17.45	36.67	107.43	9.57	15.94
5.22	8.91	8.13	34.93	20.16	45	125.95	10.845	18.56
5.92	6.55	10.05	32.92	19.55	37.85	109.2	9.09	13.76
6.62	9.35	6.08	37.81	21.24	49.68	144.74	12.2	16.43
3.82	5.61	4.08	29.65	14.46	39.77	102.7	9.92	13.42
3.37	6.22	7.4	33.49	18.28	46.08	104.17	9.94	12.78
7.22	8.7	7.78	40.62	23.18	53.07	134.74	11.16	18.09
8.9	9.32	12.95	50	28.49	60.47	160.5	14.54	13.71
6.08	9.01	7	38.59	24.84	59.8	132	10.665	15.82
.	.	.	.	.	.	105.93	8.95	15.7
7.09	11.07	5.94	47.19	27.15	65.3	168.16	14.135	31.38
.	.	.	42.35	24.13	.	153.29	13.845	.
.	.	.	.	.	.	300	33.695	69.75
.	.	.	.	.	.		.	.
.	.	.	.	.	.	.	.	.
.	.	.	.	.	.	271.06	38.87	82.76
.	.	.	.	.	.	.	.	.
.	.	.	.	.	.	.	.	.
.	.	.	.	.	.	265.6	26.585	47.17
.	.	.	.	.	.	278.22	28.15	52.88
.	.	.	.	.	.	.	.	.
.	.	.	.	.	.	.	.	.
.	.	.	.	.	.	.	.	.
.	.	.	.	.	.	.	.	.
.	.	.	.	.	.	.	.	.
.	.	.	.	.	.	.	.	.
.	.	.	.	.	.	.	.	.
.	.	.	.	.	.	.	.	.
.	.	.	.	.	.	.	.	.

.	.	.	.	.	.	.	.	.
.	.	.	.	.	.	.	.	.
.	.	.	.	.	.	.	.	.
.	.	.	.	.	.	.	.	.
.	.	.	.	.	.	.	.	.
.	.	.	.	.	.	.	.	.
.	.	.	.	.	.	.	.	.
.	.	.	.	.	.	.	.	.
.	.	.	.	.	.	.	.	.
.	.	.	.	.	.	.	.	.
.	.	.	.	.	.	.	.	.
						.	.	.
						.	.	.
.	.	.	.	.	.	.	.	.
.	.	.	.	.	.	.	.	.
12.93	17.26	21.87	76.06	50.24	106.46	.	.	.
.	.	.	.	.	.	.	.	.
.	.	.	.	.	.	.	.	.
.	.	.	.	.	.	.	.	.
.	.	.	.	.	.	.	28.785 .	
.	.	.	.	.	.	232.21	30.385	62.03
.	.	.	.	.	.			
15.11	19.8	36.26	87.74	55	150.83	266.01	33.27	47.52
19.56	23.37	25.98	86.65	67.85	148.1	.	.	.
19.34	25	33.27	103.46	78.42	170.32			
.	.	.	.	.	.	275.31	35.27	62.43
.	.	.	.	.	.	273.63	32.7	54.59
.	.	.	.	.	.	218.8	26.2	35.22
.	.	.	.	.	.	278.71	35.155	54.96
.	.	.	.	.	.	.	.	.
.	.	.	.	.	.	.	.	.
.	.	.	.	.	.	.	.	.
.	.	.	.	.	.	.	.	.
.	.	.	.	.	.	.	.	.
.	.	.	.	.	.	.	.	.
.	.	.	.	.	.	.	.	.
.	.	.	.	.	.	.	.	.
.	.	.	.	.	.	.	.	.
.	.	.	.	.	.	.	.	.
.	.	.	.	.	.	.	.	.
.	.	.	.	.	.	.	.	.
.	.	.	.	.	.	.	.	.
.	.	.	.	.	.	213.23	28.21	.
18.48	21.5	38	101.86	70.25	200	286.67	43.33	63.85
.	.	.	.	.	.	298.77	36.68	73.25
.	.	.	.	.	.	.	.	.
22.41	21.55	38.09	.	.	.	297.98	40.295	93
.	.	.	.	.	.	260.72	39.63	47.39
.	.	.	.	.	.			

.	.	.	.	.	.			
.	.	.	.	.	.	293.44	38.905	67.86
.	.	.	.	.	.	290	46.775	67.85

F6	F7	F8	F9	F10	F11	F12	F13	F14
32.96	15.55	13.27	23.15	5.7	35.39	7.1	23.67	8.88
10.48	6.03	5.42	9.24	2.75	10.52	3.3	12.61	2.93
.	13.44	12.42	16.55	4.62	.	4.65	29.34	6.35
21.45	11.81	9.84	15.8	3.5	20.12	4.11	19.89	7.25
20.98	12.06	9.01	11.91	2.92	21.85	3.45	18.43	7.35
18.92	9.85	8	11.43	2.1	15.95	3.65	16.98	4.48
17.62	8.72	8.36	10.9	2.19	19.39	3.87	17.8	6.38
17.81	10.77	7.63	12.03	3.09	17.27	4.27	21.07	4.91
16.26	9.55	7.12	11.04	2.69	17.84	2.94	15.25	4.48
19.79	11.07	9.03	16.88	4.06	20.07	10	19.84	5.6
15.27	8.8	7.15	14.84	2.93	14.41	2.93	15.95	7.16
18.98	12.77	8.39	12.07	3.86	22.19	3.66	18.26	5.96
17.55	12.27	7.52	15.23	3.87	21.64	3.67	16.34	6.61
18.69	12.22	8.33	15.7	4.12	24.3	4.26	18.62	11.16
39.49	19.19	18.01	27.46	4.54	37.08	7.1	37.65	20.8
28.24	14.93	14.02	17.16	5.61	26.19	5.44	30	14.43
30.36	15.52	15.49	19.53	5.28	31.83	8.39	31.46	16.61
28.42	15.58	15.04	16.84	4.5	23.41	4.83	32.43	17
32.32	17.39	15.54	16.63	4.41	12.05	5.32	29.01	8.7
31.43	15.17	14.54	14.38	4.17	15.54	5.46	32.77	15.2
28.03	17.15	16.22	19.63	4.47	25.93	5.73	34.67	10.03
35.55	19.63	17.96	24.4	5.36	32.63	6.59	35.56	13.85
34.48	20	18.19	24.09	6.53	33.46	5.36	39.12	19.6
32.4	14.93	13.94	26.72	5.72	35.58	4.71	39.45	9.18
26.33	14.6	13.1	20.7	4.76	33.48	4.76	26.62	16.09
28.84	15.56	12.7	18.84	5.36	33.92	4.36	30.89	17.7
41.3	20.04	18.07	29.25	7.32	46.47	7.68	34.48	12.96
31.09	17.07	14.65	23.91	5.45	23.26	4.17	32.85	13.79
22.68	11.74	9.03	15.87	5.12	22.4	5.48	21.56	10.31
19.32	10.16	7.42	14.23	3.14	18.59	3.03	16.47	4.53
18.94	13.53	8.67	11.87	4.18	20.2	4.02	18.12	5.96
22.23	10.63	8.8	12.31	3.68	25.06	4.45	22.97	6.19
22.6	10.81	9.24	14.52	3.61	19.22	4.07	17.91	5.45
42.82	20.93	13.78	25.76	5.63	41.52	15.52	34.75	30
62.34	30.02	25.06	36.83	11.74	65.98	12.76	52.93	25.8
36.75	17.74	13.02	19.35	6.64	34.75	7.5	27.2	6.93
27.65	14.17	11.27	17.79	6.97	30.24	9.4	24.45	9.02
83.65	34.96	29.09	47.58	8.1	77.22	9.44	51.45	28.66
66.93	36.01	24.05	38.63	5.8	63.69	10.24	45.47	20.72
80.64	36.01	28.26	45.26	7.76	79.23	17.35	60.82	28.01
57.38	29.7	21.4	34	5.63	60.79	8.77	46.83	23.7
77.29	37	28.76	48.07	9.04	76.3	17.29	51.79	27.5
25.67	12.62	10.76	17.27	3.33	19.06	4.08	22.5	7.45
45.25	20.02	17.55	23.17	9.42	45.64	15.4	31.26	12.8
65.5	30.74	25.45	43.06	9.29	78.93	16.42	57.11	24.67

68.28	30.37	25.77	41.43	8.14	66.5	14.97	54.86	33.25
41.77	21.73	16.46	21.55	6.32	47.86	16.43	31.53	18.25
62.47	32.66	24.46	41.02	5.47	62.23	7.92	47.19	30.17
69.07	29.13	26.12	40.84	9.64	43.69	11.19	47.39	33.15
81.83	35.79	28.86	45.49	13.48	98.55	15.06	69.6	43.44
72.43	31.5	23.9	44.16	8.71	71.82	11.56	52.54	24.34
.	.	.	.	.	.	.	.	.
115.58	56.28	50.44	89.75	16.72	130.42	24.03	.	.
.	.	.	.	.	.	.	.	.
.	.	.	.	.	.	.	.	.
100.62	47.06	41.03	58.52	11.2	102.78	15.8	76.99	37.98
.	.	.	.	.	.	.	.	.
26.18	12.38	11.36	17.83	3.64	16.76	4.24	23.66	14.98
25.13	12.01	10.88	13.8	3.17	25.39	4.94	20.17	7.78
37.33	19.76	13.12	20.07	3.22	33.59	3.95	25.24	16.04
25.24	12.25	10.57	14.56	4.33	26.61	3.77	31.69	6.64
15.91	8.37	7.35	10	2.39	13.45	2.72	15.57	5.61
17.27	7.94	7.04	11.51	3.55	12.03	3.73	23.66	8.63
24.01	13.84	10.26	14.26	3.96	21.87	4.19	21.18	12.64
29.58	14.7	12.33	16.64	5.93	16.42	3.38	25.45	19.42
25.61	11.89	11.62	16.55	3.76	24.64	4.47	25.4	10.4
31.51	17.6	14.62	23.89	4.11	32.95	5.71	32.68	11.83
24.22	13.27	10.21	13.5	3.28	29.33	3.35	21.53	14.86
23.92	12.06	10.53	17.3	4.24	26.14	3.59	20.49	10.38
29.39	14.81	13.26	20.61	4.7	23.54	6.81	27.46	20.7
42.63	21.67	16.03	24.43	8.29	41.55	7.44	30.9	17.12
31.29	15.21	11.99	22.96	3.31	19.6	3.64	26.23	11.55
25.5	13.64	12.46	14.91	4.56	25.87	5.01	22.6	11.78
40.19	16.69	15.52	22.52	3.92	43.53	8.6	32	17.57
.	16.17	15.73	.	.	.	.	36.75	.
95.29	46.47	36.8	54.71	8.75	98.16	19.5	70.27	39.52
.	.	.	.	.	.	.	.	.
.	.	.	.	.	.	.	.	.
106.75	48.84	48.86	55.18	13.91	112.82	22.33	92.27	19.19
.	.	.	.	.	.	.	.	.
.	.	.	.	.	.	.	.	.
75.74	37.49	36.67	48.18	11.59	85.97	9.99	71.69	26.88
85.64	39.93	37.41	45.58	17.59	92.14	18.16	77.76	27
.	.	.	.	.	.	.	.	.
.	.	.	.	.	.	.	.	.
.	.	.	.	.	.	.	.	.
.	.	.	.	.	.	.	.	.
.	.	.	.	.	.	.	.	.
.	.	.	.	.	.	.	.	.
.	.	.	.	.	.	.	.	.
.	.	.	.	.	.	.	.	.
.	.	.	.	.	.	.	.	.

.	.	.	.	.	.	.	.	.
.	.	.	.	.	.	.	.	.
.	.	.	.	.	.	.	.	.
.	.	.	.	.	.	.	.	.
.	.	.	.	.	.	.	.	.
.	.	.	.	.	.	.	.	.
.	.	.	.	.	.	.	.	.
.	.	.	.	.	.	.	.	.
.	.	.	.	.	.	.	.	.
.	.	.	.	.	.	.	.	.
.	.	.	.	.	.	.	.	.
.	.	.	.	.	.	.	.	.
.	.	.	.	.	.	.	.	.
.	.	.	.	.	.	.	.	.
.	.	.	.	.	.	.	.	.
.	.	.	.	.	.	.	.	.
.	.	.	.	.	.	.	.	.
.	.	.	.	.	.	.	.	.
.	.	.	.	.	.	.	.	.
.	.	.	.	.	.	.	.	27.69
94.19	36.05	35.5	36.39	11.68	81.64	11.17	63.9	29.27
89.12	50.69	44.87	41.14	8.7	93.97	15.72	59.84	32.14
.	.	.	.	.	.	.	.	.
		.	.	.	.	.	.	.
97.09	47.96	45.37	51.09	15.03	91.17	8.26	79.54	34.11
92.69	45.88	42.11	50.03	15.83	96.3	8.06	77.8	33.37
69.45	29.82	29.39	37.33	8.82	65.34	6.87	51.2	25.72
93.58	48.32	46.4	48.15	11.77	89.72	7.24	78.17	34.83
.	.	.	.	.	.	.	.	.
.	.	.	.	.	.	.	.	.
.	.	.	.	.	.	.	.	.
.	.	.	.	.	.	.	.	.
.	.	.	.	.	.	.	.	.
.	.	.	.	.	.	.	.	.
.	.	.	.	.	.	.	.	.
.	.	.	.	.	.	.	.	.
.	.	.	.	.	.	.	.	.
.	.	.	.	.	.	.	.	.
.	.	.	.	.	.	.	.	.
.	.	.	.	.	.	.	.	.
.	.	.	.	.	.	.	.	.
.	33.87	30.96						22.57
126.31	62.06	46.4	61.96	13.77	116.73	18.5	81.29	41.73
104.72	51.47	48.54	53.84	16.33	101.78	14.78	78.55	45.86
.	.	.	.	.	.	.	.	.
123.94	58.17	51.12	47.58	15.52	47.61	18.05	76.97	31.56
108.58	50.02	42.81	66.99	12.63	90.1	19.31	69.96	42.68

110.86	53.88	50.2	47.82	12.44	58.86	15.89	90.64	38.36
122.8	48.98	47.87	59.24	14.16	118.88	16.94	86.76	46.55

F15	F16	F17	F18	F19	F20	F21	F22	F23
67.81	16.14	11.14	8.98	23.88	23.91	26.1	16.25	18.1
27.06	7.17	4.26	3.95	8.51	8.18	9.4	9.55	9.22
66.12	15.82	11	8.18	20.49	21.96	22.99	15.58	14.89
38.33	13.92	8.16	7.17	15.76	15.39	19.71	15.08	14.17
38.11	13.37	7.55	6.84	16.02	15.59	18.31	12.6	11.21
26.74	12.87	6.71	5.67	12.88	13.09	15.17	11.74	10.06
32.89	11.52	6.99	5.87	13.87	12.04	15.44	10.89	9.55
33.18	11.33	6.74	5.28	13.69	13.12	15.41	11.32	10.07
31.5	9.96	7.05	5.11	12.05	10.78	14.44	14.57	13.31
48.38	10.84	6.44	5.95	17.61	16.98	16.08	13.91	14.94
38.29	9.27	6.03	4.94	12.69	12.5	13.44	11.89	10.93
39.37	11.44	7.59	5.62	14.65	13.4	16.19	12.63	12.07
39.94	11.74	8.03	5.95	13.46	12.41	16.4	11.73	10.01
42.05	13.75	8.21	5.72	13.91	12.74	16.34	12.92	10.84
77.15	24.41	14.37	12.79	27.73	26.22	35.46	20.19	18.66
65.86	16.95	10.91	9.15	22.92	19.76	26.37	16.31	16.04
66.9	18.1	11.9	9.2	23.84	22.54	24.41	17.36	15.9
64	18.76	11.4	9.64	22.16	21.76	27.78	15.94	14.45
67.77	19.37	11.53	10.57	21.45	21.69	27.99	16.44	15.61
62.32	18.26	12.29	11.55	22.92	21.07	28.61	18.16	17.27
67.06	18.4	11.12	9.48	20.08	18.48	26.95	18.46	17.36
71.4	21.76	13.95	11.38	26.53	25.27	30.97	19.35	18.82
79.46	21.16	13.49	11.69	24.94	24.37	31.33	17.31	16.11
74.96	17.41	10.74	9.74	24.04	22.95	25.3	19.84	18.14
60.86	16.87	10.87	9.68	22.51	21.17	25.04	19.21	17.62
69.6	17	11.96	10.95	24.75	22.9	26.38	18.09	16.8
81.89	32.82	15.51	11.83	33.79	31.14	34.1	25.72	23.69
64.2	17.5	12.35	9.64	24.24	22.94	26.04	15.61	19.96
50.43	11.52	8.25	6.79	16.15	15.36	17.8	14.59	14.57
38.05	11.5	7.13	5.7	16.03	13.78	16.06	12.29	11.7
38.23	12.46	7.96	6.23	15.13	13.89	17.04	17.17	11.5
43.5	12.82	8.85	6.48	18.78	18.08	18.77	14.28	13
44.9	13.66	8.95	6.68	19.61	18.46	18.67	15.68	13.77
71.5	22.39	15.34	12.46	36.55	31.39	33.43	24.46	23.09
113.9	32.66	22.09	18.56	42.87	40.92	45.69	35.69	30.12
65.17	19.56	13.12	11.07	28.42	25.15	28.46	21.66	19.85
58.13	15.96	9.56	8.77	23.28	21.55	23.32	18.89	17.66
121.41	46.07	25.91	23.52	57.33	57.3	59.38	45.71	39.39
107.19	39.65	20.83	18.7	49.3	42.69	50.08	38.7	36.72
130.36	45.05	29.04	23.79	64.05	55.37	59.66	47.38	41.88
101.26	37.56	22.94	19.56	48.16	44.4	49.85	35.38	31.17
120.3	45.47	27.73	24.84	60.32	52.74	59.49	43.09	38.28
50.54	15.07	10.21	7.8	21.18	19.13	22.42	15.32	14.39
76.1	26.11	17.53	13.24	37.53	33.92	36.76	30.8	25.57
117.47	36.42	25.51	20.46	49.55	44.1	52.44	38.72	33.12

113.61	37.13	22.62	19.37	50.58	45.75	51.67	35.57	32.11
78.76	24.7	16	15.96	36.22	30.07	33.16	24.92	22.09
111.12	38.07	21.38	19.13	48.46	41.93	47.18	35.79	31.73
110.59	46.86	26.25	22.09	57.07	46.89	55.79	41.23	36.96
128.46	51.33	28.45	27.64	60.22	52.28	63.89	45.41	36.13
110.95	45.14	25.38	22.88	54.77	44.33	55.66	34.88	28.74
.	.	.	.	.	.	.	.	.
.	.	.	.	.	.	.	.	.
.	.	.	.	.	.	.	.	.
.	.	.	.	.	.	.	.	.
153.59	65.95	41.73	35.46	81.25	72.96	83.27	62.89	49.91
.	.	.	.	.	.	.	.	.
51.39	17.78	10.14	8.18	21.47	19.6	22.62	16.11	12.65
45.1	15.91	10.55	8.17	20.14	18.51	22.35	14.78	12.59
55.93	24.57	12.02	10.27	26.99	24.38	27.56	20.75	17.5
53.51	14.81	9.78	7.42	19.34	17.86	21.71	15.67	14.31
33.17	10.22	6.12	4.95	12.64	11.62	13.89	10.57	10.11
38.86	10.47	6.76	5.39	12.72	11.95	14.63	16.11	12.65
45.32	13.01	9.46	6.94	18.62	16.65	19.95	14.29	12.21
56.62	17.65	9.37	7.75	22.96	21.82	24.74	18.08	17.43
52.73	14.78	9.73	8.44	21.03	19.04	22.97	15.01	14
69.68	19.23	12.88	9.88	26.9	24.02	26.52	22.05	19.83
51.6	13.23	9.9	7.4	19.41	19.32	19.96	16.24	16.03
51.08	13.51	9.25	7.48	17.19	17.82	19.65	14.61	13.89
63.88	17.8	10.98	9.49	24.81	23.47	25.08	20.12	18.98
74.81	23.01	15.26	12.35	34.64	30.99	33.02	24.9	22.91
62.54	15.75	11.2	9.23	21.87	20.88	24.14	18.99	16.17
52.68	14.74	10.03	7.77	22.35	19.47	22.31	14.33	12.89
79.89	23.53	15.22	12.29	33.49	30.87	33.31	24.18	23.29
78.47	24.32	15.93	11.69	32.9	28.26	34.24	22.8	19.39
145.42	57.75	35.26	29.99	74.72	65.2	75.63	47.85	42.28
.	.	.	.	.	.	.	.	.
.	.	.	.	.	.	.	.	.
149.05	59.64	43.09	30.14	64.11	62.13	87.94	58.33	50.86
.	.	.	.	.	.	.	.	.
.	.	.	.	.	.	.	.	.
139.92	42.75	27.57	23.58	57.32	49.49	61.67	42.31	39.17
141.39	56.5	32.16	26.45	61.89	59.03	72.44	46.72	36.87
.	.	.	.	.	. .	.	.	
.	.	.	.	.	. .	.	.	
.	.	.	.	.	. .	.	.	
.	.	.	.	.	. .	.	.	
.	.	.	.	.	. .	.	.	
.	.	.	.	.	. .	.	.	
.	.	.	.	.	. .	.	.	
.	.	.	.	.	. .	.	.	
.	.	.	.	.	. .	.	.	

.	.	.	.	.	. .	.	.	
.	.	.	.	.	. .	.	.	
.	.	.	.	.	. .	.	.	
.	.	.	.	.	. .	.	.	
.	.	.	.	.	. .	.	.	
.	.	.	.	.	. .	.	.	
.	.	.	.	.	. .	.	.	
.	.	.	.	.	. .	.	.	
.	.	.	.	.	. .	.	.	
.	.	.	.	.	. .	.	.	
.	.	.	.	.	. .	.	.	
.	.	.	.	.	. .	.	.	
.	.	.	.	.	. .	.	.	
.	.	.	.	.	. .	.	.	
.	.	.	.	.	. .	.	.	
.	.	.	.	.	. .	.	.	
.	.	.	.	.	. .	.	.	
.	.	.	.	.	. .	.	.	
.	.	.	.	.	. .	.	.	
.	48.1	29.03	26.9	59.09	52.93	61.49 .	.	
128.75	53.38	28.3	27.2	60.6	54.37	65.94	45.27	39.04
144.13	57.73	31.28	28.27	62.66	56.39	66.73	38.47	32.31
.	.	.	.	.	.	.	.	.
.	.	.	.	.	.	.	.	.
156.58	57.97	34.8	31.28	70.07	62.64	75.46	52.17	51.56
162.1	58.93	33.9	30.85	70.92	62.3	74.6	47.04	45.89
120.99	37.47	23.52	21.94	48.96	46.15	50.89	34.74	33.4
157.94	57.92	34.1	30.58	69.68	62.29	74.51	46.04	45.03
.	.	.	.	.	.	.	.	.
.	.	.	.	.	.	.	.	.
.	.	.	.	.	.	.	.	.
.	.	.	.	.	.	.	.	.
.	.	.	.	.	.	.	.	.
.	.	.	.	.	.	.	.	.
.	.	.	.	.	.	.	.	.
.	.	.	.	.	.	.	.	.
.	.	.	.	.	.	.	.	.
.	.	.	.	.	.	.	.	.
.	.	.	.	.	.	.	.	.
.	.	.	.	.	.	.	.	.
.	.	.	.	.	.	.	.	.
121.03	45.17	23.62	21.22	54.48	47.43	53.64		.
174.69	69.31	40.08	37.32	83.9	75.45	89.31	54.81	45.54
164.24	66.05	38.99	33.73	74.12	69.15	81.45	55.82	53.3
.	.	.	.	.	.	.	.	.
146.4	63.66	39.32	39.22	83.03	76.23	91.15	48.35	46.96
186.41	63.22	33.62	32.36	76.37	69.44	78.85	45.39	42.3

160.45	62.24	34.21	36.68	80.7	72.11	82.61	56.34	51.29
171.11	61.34	41.19	39.29	79.33	74.55	95.49	51.68	48.54

F24	T1	T4	T5	T6	T7	T8	T9	T10
12.83	189.7	10.345	45.93	8.82	21.21	21.91	16.29	8.35
8.83	67.21	3.575	16.83	3.28	8.09	9.28	6.11	3.99
14.31	127.48	9.44	38.14	3.64	17.14	18.9	13.16	7.94
11.41	126.19	7.795	34.79	7.4	19.78	18.81	12.42	7.72
8.94	130.48	8.62	31.74	5.62	20.8	18.87	12.93	5.96
9.34	108.17	5.91	24.8	3.09	16.55	15.59	11.22	5.57
6.55	108.95	7.41	28.38	5.39	16.55	15.16	10.43	5.73
8.47	106.89	6.595	23.94	4.45	16.13	15.25	10.74	5.64
10.96	99.63	5.275	21.81	6.08	16.11	13.79	9.78	4.96
13.11	102.17	6.375	26.9	6.75	15.91	15.61	11.82	5.52
9.41	94.01	5.145	23.05	4.1	13.25	12.97	11.54	5.54
8.94	117.13	6.935	31.42	5.15	17.3	15.62	11.19	5.63
8.06	107.84	5.84	25.29	4.29	17.18	14.8	11	5.11
9.16	116.93	6.79	26.6	7.44	17.98	15.36	10.3	5.61
23.39	155.1	13.12	47.12	8.54	28.18	33.86	22.86	12.02
14.49	.	.	.	.	.	.	.	.
13.96	120.73	9.46	44.95	5.88	24.11	25.69	17.93	9.98
14.63	121.24	9.375	43.46	4.14	20.77	26.39	18.08	9.2
14.62	127.64	11.1	43.82	7.13	22.86	26.83	18.55	9.02
16.2	138.22	10.91	38.36	5.19	24.67	26.89	17.36	11.87
16.12	133.86	9.545	42.49	4.82	21.48	24.68	18.54	10.41
18.17	145.19	12.45	52.91	7.43	23.1	29.06	21.97	10.34
15.99	136.72	9.88	52.35	6.31	23.74	30.53	19.94	9.78
16.6	166.1	10.14	52.73	7.52	24.78	26.09	15.7	9.05
14.9	159.56	10.155	41.92	5.79	25.34	24.64	15.36	8.48
16.52	.	.	.	.	.	.	.	.
23.6	226.21	12.955	61.21	8.62	31.88	30.98	18.37	9.7
14.36	189.7	10.345	45.93	8.82	27.52	25.66	14.27	9.13
11.9	126.09	6.57	40.11	7.32	16.44	17.58	10.88	6.57
10.46	127.93	7.205	27.03	4.58	17.61	15.58	10.17	5.33
9.51	118.52	8.17	25.88	7.54	16.58	15.68	10.57	6.22
12.04	145.09	7.195	38.22	6.06	20.4	20.43	12.98	5.7
12.48	153.15	7.46	37.17	9.87	23	18.1	12.52	6.13
20.71	247.59	13.78	61.32	13.22	38.34	33.89	22.05	11.47
25.62	359.26	16.535	77.99	16.86	52.11	47.93	29.53	15.95
16.98	201.98	12.015	49.16	8.86	29.73	26.91	19.13	9.16
14.9	159.98	10.06	44.52	8.28	25.5	22.39	15.29	7.28
35.07	545	25.56	151.09	28.75	70.13	60.05	38.42	20.54
27.3	480	23.345	98.08	18.51	58.87	48.6	29.9	17.92
36.75	517	26.755	131.37	15.08	70.41	56.49	35.22	22.04
27.13	401.2	24.155	102.58	15.14	55.64	47.6	32.78	17.17
36.02	530	24.365	142.9	23.84	69.89	59.96	36.64	20.63
12.82	145.77	8.46	35.44	6.47	19.76	20.31	14.32	7.81
21.59	261.69	14.69	57.7	9.12	39.24	34.86	24.92	12.23
27.57	410	19.455	105.22	14.9	53.72	52.45	31.32	16.06

27.51	507	24.005	89.1	16.66	57.64	49.97	32.14	16.68
18.88	233.84	13.49	56.17	9.46	36.14	35.07	23.04	12.93
25.49	421	18.865	103.06	20.34	53.87	46.12	28.46	16.71
27.64	486.81	22.07	103.48	12.62	60.47	54.12	33.81	20.88
34.6	509	28.775	132.13	17.79	71.65	63.51	38.76	21.03
29.6	500	25.305	128.06	21.06	62.59	55.85	32.61	19.05
.	625	40.135	.	.	93.93	74.28	.	.
.	590	39.165	.	29.24	.	.	47.68	23.03
.	.	.	.	.	.	.	.	.
.	.	.	.	.	.	.	.	.
47.09	.	.	.	.	.	.	.	.
.	.	.	.	.	.	.	.	.
12.33	172.4	8.855	37.64	7.99	24.78	22.26	13.96	8.5
10.48	155.29	8.505	42.4	6.39	22.95	21.49	14.44	6.91
15.25	205.18	11.135	45.07	9.57	31.68	27.11	17.94	8.29
12.01	156.09	9.47	39.53	5.14	21.54	20.83	12.69	8.01
8.53	90.03	5.43	26.99	4.83	12.46	13.7	8.99	4.86
12.33	.	.	.	.	.	.	.	.
11.07	151.72	9.89	35.93	4.84	21.19	20.01	10.93	7.35
15.07	173.7	10.085	41.24	8.29	24.98	24.11	14.22	8.27
12.22	152.48	8.04	36.68	5.41	22.71	20.59	16.59	9.39
16.89	225.93	14.34	63.02	8.57	31.55	30.42	18.98	9.63
13.15	118.47	7.77	34.8	5.73	20.34	18.9	12.81	6.16
12.12	119.7	8.02	34.39	6.47	19.49	18.93	8.98	7.01
17.03	174.93	11.18	47.74	9.21	27.72	24.32	14.96	9.06
19.9	144	12.56	63.91	10.41	37.21	33.33	18.6	8.9
14.59	184.4	9.62	52.92	9.27	25.1	23.39	14.33	8.06
12.85	135.93	7.555	34.64	6.1	21.69	20.96	14.22	7.16
20.53	243.1	13.63	59.08	11.38	32.69	33.82	21.88	13.49
19.23	.	.	.	.	.	.	.	.
40.42	501	32.17	161	41.12	93.83	84.31	45.32	25.54
.	.	.	.	.	.	.	.	.
.	483	27.24	126.15	14.02	67.83	65.48	39.83	20.4
35.22	.	.	.	.	.	.	41.11	24.57
.	.	.	.	.	.	.	37.76	18.59
.	.	.	.	.	.	.	.	.
34.26	.	.	.	.	.	.	.	.
29.52	.	.	.	.	.	.	.	.
.	493	28.5	199	14.1	67.68	64.86	42	21.81
.	.	.	.	.	.	.	.	.
.	.	.	.	.	.	.	.	.
.	.	.	.	.	.	.	.	.
.	.	.	.	.	.	.	.	.
.	.	.	.	.	.	.	.	.
.	.	.	.	.	.	.	.	.
.	.	.	.	.	.	.	.	.
.	.	.	.	.	.	.	.	.

.	.	.	.	.	.	.	.	.
.	.	.	.	.	.	.	.	.
.	.	.	.	.	.	.	.	.
.	.	.	.	.	.	.	.	.
.	.	.	.	.	.	.	.	.
.	.	.	.	.	.	.	.	.
.	.	.	.	.	.	.	.	.
.	.	.	.	.	.	.	.	.
.	.	.	.	.	.	.	.	.
.	.	.	.	.	.	.	.	.
.	.	.	.	.	.	.	.	.
.	575	41.58	159.85	20.86	92.12	98.32	57.93	31.59
.	.	.	.	.	.	.	.	.
.	.	.	.	.	.	.	.	.
.	.	.	.	.	.	.	.	.
.	.	.	.	.	.	.	.	.
.	341	25.065	87.66	13.76	56.98	51.65	34.07	17.29
.	320	21.64	79.11	17.98	53.72	49.55	30.61	16.51
.	315	22.765	91.02	11	54.05	46.6	30.62	15.94
.	.	.	.	.	.	.		
29.47	383	30.045	116.37	15.29	68.53	61.83	37.98	22
	304	21.265	95.49	18.54	53.76	49.97	32.57	22.71
28.77	412	31.17	124.75	11.14	72.63	68.82	41.67	25.76
.	.	.	.	.	.	.	.	.
.	.	.	.	.	.	.	.	.
36.98	.	.	.	.	.	.	.	.
33.12	.	.	.	.	.	.	.	.
28.48	.	.	.	.	.	.	.	.
34	.	.	.	.	.	.	.	.
.	400	29.86	112.31	15.32	73.2	68.97	44.44	24.41
.	434	29.42	124.24	13.97	74.76	69.88	45.95	23.43
.	437	30.085	132.66	16.32	80.53	72.72	46.87	23.36
.	472	35.21	124.41	16.03	85.84	85.84	49.34	26.91
.	.	.	.	.	.	.	.	.
.	.	.	.	.	.	.	.	.
.	.	.	.	.	.	.	.	.
.	.	.	.	.	.	.	.	.
.	.	.	.	.	.	.	.	.
.	.	.	.	.	.	.	.	.
.	.	.	.	.	.	.	.	.
.	.	.	.	.	.	.	.	.
.	.	.	.	.	.	.	.	.
.	.	.	.	.	.	.	.	.
38.33	552	38.355	158.5	17.77	90.6	87.05	52.9	24.44
39.49								
.	513	34.77	203	20	90.92	76.35	40.8	21.27
33.86	535	34.765	191.8	40	73.8	83.46	47.41	23.88
42.17	502	35.115	131.09	23.81	76.33	82.7	47.59	23.9

36.48	500.1	37.575	153.55	41.38	84.89	88.15
39.33						

T11	T12	T13	T14	T15	A1	A2	A3	A4
10.06	8.51	3.1	5.31	10.63	15.85	17.29	6.8	5.25
4.59	4	2.69	2.6	5.1	5.76	6.38	3.45	2.19
7.74	7.07	5.08	3.58	9.66	.	.	.	.
8.76	7.65	4.22	3.02	9.27	9.87	13.61	5.57	3.62
10	7.13	4.37	3.72	7.94	10.09	13.02	7.09	3.94
7.68	5.45	3.29	3.03	6.56	8.03	8.42	5.42	3.17
6.17	5.58	4.4	2.99	7.21	7.34	10.51	5.06	3.26
7.09	5.99	3.98	3.13	6.9	7.71	10.38	4.92	2.58
6.33	5.04	3.14	2.47	5.89	6.65	9.22	4.73	2.12
6.61	5.95	5.97	3.41	6.99	9.99	11.86	6.07	3.55
5.74	4.93	3.21	2.46	6.57	7.27	9.24	4.8	2.35
6.87	7.9	4.1	3.46	6.55	7.89	10.9	5.31	3.4
6.36	5.72	3.25	3.11	6.01	8.05	11.36	5.11	2.89
6.82	5.72	3.29	2.6	6.2	7.44	10.71	5.22	2.84
13.38	12.17	7.04	5.95	13.63	18.84	27.04	10.44	6.44
.	.	.	.	.	.	.	.	.
11.42	8.86	6.01	4.77	11.27	14.39	20.91	8.43	5.4
12.01	9.59	6.65	6.13	11.04	15.22	21.14	8.67	5.33
12.66	9.78	4.03	4.69	12.92	13.18	21.41	10.44	7.51
12.82	9.84	5.85	4.78	12.04	16.12	22.19	12.38	7.74
11.46	9.54	5.32	4.64	12.27	14.59	20.03	7.57	5.3
16.03	12.3	7.77	7.49	11.79	17.76	25.57	10.6	6.09
13.77	10.96	6.71	5.08	11.77	15.19	22.32	10.11	5.95
9.68	9.94	5.8	4.9	11.64	12.74	16.21	8.33	3.86
8.46	8.9	5.04	3.66	11.14	12.45	15.94	7.64	3.19
	.	.	.	.	.	.	.	.
10.95	11.53	7.37	4.95	12.03	16.83	19.31	10.72	4.86
11.51	8.95	5.9	4.72	12.28	12.95	16.49	9.53	4.6
9.25	6.45	3.97	2.84	7.81	9.52	10.4	6.88	2.88
5.77	5.83	3.4	3.73	7.45	9.33	12	6.4	2.84
7.63	7.53	3.54	4.13	6.79	8.37	11.56	5.75	3.85
7.25	7.17	3.95	3.74	7.59	10.12	12.94	6.34	3
7.79	7.94	4.21	3.28	8.84	10.81	12.28	6.94	3.16
14.81	12.26	7.32	6.85	14.19	20.22	23.67	12.27	5.84
20.24	19.51	10.04	8.68	18.9	26.38	31.02	17.18	7.35
11	10.7	6.16	6.61	12.65	16.75	19.63	9.56	4.77
10.54	8.54	5.17	4.97	9.28	13.18	15.47	7.44	4.42
22.23	20.47	10.79	9.76	24.51	31.71	39.87	17.94	9.33
17.38	15.67	9.34	10.63	19.95	26.94	30.13	16.04	7.33
23.51	22.79	11.81	11.76	24.02	30.84	37.83	20.02	9.62
21.28	19.79	12.2	9.19	22.26	28.78	30.93	15.51	7.54
22.54	22.08	13.91	12.43	23.02	33.2	38.86	18.79	9.37
8.78	8.54	4.39	5.77	9.32	11.92	14.96	6.69	3.86
14.99	14.02	7.16	9.16	15.04	22.57	25.83	14.52	6.93
19.36	18.38	12.09	9.38	21.26	27.17	33.75	15.56	7.48

20.19	18.17	11.17	9.22	20.1	27.27	32.79	16.2	7.51
14.03	13.19	8.2	6.83	13.56	19.26	23.29	11.96	6.04
17.85	17.39	9.39	9.32	18.92	28.46	31.6	14.2	7.64
23.48	24.21	12.79	10.77	24.28	32.21	36.96	18.05	8.16
22.98	23.88	13.93	9.98	25.65	36.39	39.43	18.58	9.88
19.11	21.82	12.5	8.43	22.45	31.91	34.1	17.15	8.53
.	.	.	.	.	.	.	.	.
30.57	20.73	16.84	15.39	30.76	45.94	50.67	28.67	12.53
.	.	.	.	.	43.14	45.63	26.01	11.79
.	.	.	.	.	49.06	55.83	31.82	16.74
.	.	.	.	.	43.67	47	21.47	14.41
.	.	.	.	.	44.35	52.65	25.63	12.07
9.17	8.69	4.58	4.02	9.17	12.04	15.05	7.33	3.52
9.73	9.03	5.18	4.29	10.6	9.67	15.72	6.73	4.1
11.8	10.59	5.07	5.3	12.28	17.1	18.79	9.53	5.13
8.39	8.39	4.51	4.38	9.65	14.2	13.87	6.97	3.67
5.21	5.76	3.09	2.32	6.5	.	.	.	.
.	.	.	.	.	.	.	.	.
8.01	7.86	4.2	2.67	8.67	11.73	13.04	6.69	3.8
12.84	9.07	4.98	4.68	10.71	15.18	13.69	8.2	4.4
9.79	8.5	4.9	5.43	11.1	15.91	16.49	8.07	4.43
12.68	12.03	6.96	6.33	12.83	16.67	18.47	9.3	4.43
7.63	7.15	4.38	4.24	7.93	10.82	13.77	6.92	3.88
8.6	7.29	4.26	3.85	9.44	11.19	14.22	6.2	3.52
10.01	9.17	6.15	5.29	12.11	14.15	17.33	7.88	4.15
11.97	8.71	6.92	7.44	12.86				
9.15	8.79	5.06	3.76	9.54	12.32	15.9	9.39	4.46
9.08	7.88	4.62	6.04	9.1	12.74	15.34	8.57	3.58
15.08	12.92	7.17	7.16	14	19.47	22.93	13.48	5.48
.	.	.	.	.	17.09	20.66	10.12	5.48
26.62	28.2	15.54	15.47	29.27	40.39	48.25	26.04	10.5
.	.	.	.	.	.	.	.	.
25.52	23.51	14.22	9.97	24.29	38.35	33.34	20.53	9.2
27.33	21.49	16.92	15.55	26.9	36.98	43.6	25.61	9.96
22.16	21.36	13.34	12.6	23.74	.	.	.	.
.	.	.	.	.	37.14	42.97	25.1	12.44
.	.	.	.	.	.	.	.	.
.	.	.	.	.	.	.	.	.
27.41	20.22	11.14	11.13	27.51	.	.	.	.
.	.	.	.	.	34.85	38.85	21	11.52
.	.	.	.	.	38.67	45.42	25.14	13.66
.	.	.	.	.	.	.	.	.
.	.	.	.	.	.	.	.	.
.	.	.	.	.	.	.	.	.
.	.	.	.	.	.	.	.	.
.	.	.	.	.	.	.	.	.
.	.	.	.	.	.	.	.	.

.	.	.	.	.	.	.	.	.
.	.	.	.	.	.	.	.	.
.	.	.	.	.	.	.	.	.
.	.	.	.	.	.	.	.	.
.	.	.	.	.	.	.	.	.
.	.	.	.	.	.	.	.	.
.	.	.	.	.	.	.	.	.
.	.	.	.	.	.	.	.	.
.	.	.	.	.	.	.	.	.
.	.	.	.	.	.	.	.	.
.	.	.	.	.	26	30.46	16.38	8.82
32.77	31.98	15.58	22.08	45.24	57.04	61.59	32.58	19.6
.	.	.	.	.	51.51	53.98	33.48	18.32
.	.	.	.	.	33.11	34.07	18.73	12.1
.	.	.	.	.	.	.	.	.
.	.	.	.	.	.	.	.	.
18.7	18.49	9.67	12.73	21.75	.	.	.	.
22.85	18.04	7.74	11.91	22.96	.	.	.	.
19.79	17.37	7.08	10.4	22.83	.	.	.	.
					.	.	.	.
22.51	22.2	12.21	17.69	29.67	.	.	.	.
23.19	17.5	7.33	10.41		.	.	.	.
28.88	25.68	11.11	17.21	34.34	41.61	43.64	25.49	15.15
.	.	.	.	.	.	.	.	.
.	.	.	.	.	.	.	.	.
.	.	.	.	.	.	.	.	.
.	.	.	.	.	.	.	.	.
.	.	.	.	.	.	.	.	.
.	.	.	.	.	.	.	.	.
26.07	24.35	11.84	16.52	32.59	.	.	.	.
25.16	25.71	11.47	14.59	33.76	.	.	.	.
28.36	26.58	11.92	18.56	33.31	.	.	.	.
27.77	28.32	13.91	19.06	37.79	.	.	.	.
.	.	.	.	.	40.77	37.95	24.74	14.77
.	.	.	.	.	40.56	41.25	24.71	16.79
.	.	.	.	.	39.17	41.81	.	.
.	.	.	.	.	.	.	.	.
.	.	.	.	.	40.5	41.89	26.35	15.52
.	.	.	.	.	.	.	.	.
.	.	.	.	.	.	.	.	.
.	.	.	.	.	.	.	.	.
.	.	.	.	.	.	.	.	.
.	.	.	.	.	28.82	32.3		
32.2	31	14.44	18.93	31.49	52.57	53.57	27.98	15.51
23.26	28.9	12.43	17.05	34.84	41.47	45.24	23.27	14.39
33.23	27.27	11.81	17.07	35.11	43.09	48.91	26.31	15.56
33.59	27.94	12.44	16.27	33.36	41.95	46.16	26.48	14.98
					45.33	52.51	29.26	15.79

43.62	47.04	20.14	15.54

A5	A6	A7	A8	A9	A10	C1	C2	C3
9.61	10.54	11.21	6.01	10	11.4	30.35	32.07	30.15
4.39	4.17	5.44	1.84	4.72	5.69	8.7	10.92	10.1
.	.	.	.	.	.	18.45	19.5	18.88
7.53	7.59	6.53	3.2	8.45	9.68	18.33	22.31	20.98
6.33	8.34	7.5	5.07	9.06	9.14	22.22	23.65	22.53
6.23	7.61	6.06	2.44	6.99	6.93	17.86	18.79	16.65
5.31	7.48	3.53	3.4	6.52	6.75	16.9	19	16.59
6.15	6.77	5.9	3.73	7	7.14	16.73	19	16.52
5.8	6.81	4.67	2.3	6.33	6.57	15.68	17.43	16.08
6.88	7.06	6.9	4.34	7.91	8.11	20.48	21.76	20.38
5.48	5.49	7.23	3.26	6.3	6.05	17.71	16.66	15.56
6.13	8.19	4.03	3.16	7.84	7.09	18.48	21.35	18.5
6.04	7.64	4.89	3.35	6.86	6.79	17.97	21.03	19.22
5.79	7.3	5.68	3.22	7.3	6.32	20.07	21.04	19.26
13.78	15.72	11.39	6.31	17.55	16.63	33.93	35.28	34.93
.	.	.	.	.	.	.	.	.
11.3	13.34	8.55	6	13.56	13.11	25.5	30.18	27.06
11.1	13.46	9.73	5.77	13.29	13.73	25.5	27.8	24.74
12.98	11.2	11.63	7.25	14.53	12.96	31.05	33.92	31.72
10.35	13.11	10.59	7.14	13.57	12.72	27.3	29.36	29
10.07	12.03	9.12	7.17	12.92	12.05	24.01	26.55	23.91
12.47	15.37	12.5	9.16	15.75	13.28	33.14	33.7	32.32
11.68	14.96	8.28	5.4	14.05	14.5	29.47	32.52	29.07
8.72	10.53	7.58	5.06	11.56	12.22	28	27.53	25.75
8.77	10.04	6.68	4.59	10.84	11.24	24.48	26.26	25.55
.	.	.	.	.	.	.	.	.
13.91	12.04	9.73	7.83	15.5	15.73	35.78	37.51	34.6
10.87	6.81	6.5	3.71	13.11	10.67	26.41	29.38	26.98
7.75	7.27	6.16	3.49	9.28	9.47	19.72	21.61	19.7
7.27	7.08	7.27	3.89	7.13	7.59	17.92	22.12	20.02
6.57	8.91	6.02	2.47	6.37	7.61	18.47	21.12	19.35
7.32	8.38	6.23	3.49	7.75	8.35	22.02	25.05	22.56
8.15	9.02	6.63	4.63	8.46	9.49	23.66	26.9	24.79
15.02	14.53	11.69	6.07	14.99	19.41	37.08	43.34	38.16
21.26	18.02	14.86	10.96	21.87	21.11	63.13	68.01	64.89
12.65	13.12	9.36	5.42	14.3	15.71	34.77	37.71	35.67
10.04	9.77	7.57	4.39	10.8	11.61	28.85	29.6	27.72
25.15	24.28	17.84	13.23	26.37	29.52	75.04	85.88	81.85
21.6	19.98	14.34	8.59	21.89	22.05	63.07	67.77	64.04
26.05	23.3	19.3	12.28	29.83	31.92	74.25	81.46	78.45
22.8	20.16	19.1	8.87	21.16	25.56	63.57	70.39	66.25
26.05	25.56	17.45	11.88	25.72	28.88	55.34	62.48	57.75
9.83	10.03	7.38	4.61	10.05	10.76	26.44	29.45	27.07
17.51	16.6	15.27	7.8	17.33	19.4	43.64	51.02	48.08
23.26	19.63	15.56	9.04	21.79	25.37	60.8	71.8	66.72

21.3	21.46	15.31	9.88	25.81	26.4	60.27	67.81	64.8
14.13	13.28	10.46	6.15	17.15	18.38	38.96	46.47	40.47
21.39	19.09	17.26	10.62	23.25	24.87	58.83	69.5	65.11
27.96	21.19	22.21	13.11	26.52	27.04	70.3	79	73.9
31.19	21.16	22.35	13.08	30.65	31.09	85.21	93.16	89.01
24.57	19.69	20.29	12.3	22.83	28.59	55.34	62.48	57.75
.	.	.	.	.	.	92.41	.	.
33.57	34	27.37	13.74	37.9	41.68	96.18	104.48	98.94
33.85	30.99	22.33	11.45	37.22	37.45	101.61	113.39	103.95
39.66	43.18	24.28	14.43	43.28	45.1	.	.	.
34.6	36.74	17.63	17.52	31.1	40.73	96.88	107.14	95
34.22	31.85	23.39	19.57	36.44	37.69	99.42	112.3	104.55
7.8	7.89	8.92	4.42	9.46	10.13	27.06	30.5	28.06
8.81	10.96	6.83	5.36	9.18	10.15	25.01	26.64	25.06
12.09	11.95	8.23	5.38	12.35	11.4	34.95	38.59	35.63
9.72	8.49	7.58	5.22	10.21	10.57	25.09	27.79	26.44
.	.	.	.	.	.	.	.	.
.	.	.	.	.	.	.	.	.
9.53	7.98	6.83	3.8	8.69	10.49	24.84	26.99	24.94
11.35	8.09	5.6	3.04	12.7	11.44	31.13	31.65	30.51
11.55	9.85	9.27	4.59	11.28	12.86	29.75	33	32.32
12.51	10.68	9.42	4.45	13.21	13.42	35.34	38.15	35.75
8.06	7.9	6.78	4.67	8.39	9.97	24.67	26.27	25.04
8.41	8.78	7.59	5.04	9.46	9.88	24.96	26.35	25.34
10.69	10.13	8.37	6.12	12.61	12.93			
10.08	7.46	5.81	3.28	12.6	11.74	23.32	29.33	26.69
9.37	9.15	9.79	4.62	10.83	10.76	24.71	26.33	24.54
15.31	12.75	9.87	5.96	17.18	18.2	40.73	46.12	42.27
11.35	11.51	6.11	13.57	14.35	14.91	38.46	42.53	39.21
30.21	30.86	21.9	13.05	35.65	36.16	84.75	94.37	89.09
.	.	.	.	.	.	103.02	113.77	108.56
25.45	24.95	18.67	11.24	29.61	29.82	74.08	78.47	75.36
32.05	28.99	24.41	17.51	30.11	31.47	85.12	98.2	90.38
.	.	.	.	.	.	.	.	.
27.58	30.64	22.1	9.75	29.78	30.62	.	.	.
.	.	.	.	.	.	.	.	.
.	.	.	.	.	.	.	.	.
.	.	.	.	.	.	.	.	.
25.64	26.72	21.41	8.73	24.67	27.84	.	.	.
32.18	28.31	24.04	10.23	30.59	34.36	.	.	.
.	.	.	.	.	.	70.64	79.69	75.42
.	.	.	.	.	.	71.07	78.16	75.67
.	.	.	.	.	.	.	.	.
.	.	.	.	.	.	.	.	.
.	.	.	.	.	.	.	.	.
.	.	.	.	.	.	.	.	.

.	.	.	.	.	.	.	.	.
.	.	.	.	.	.	.	.	.
.	.	.	.	.	.	.	.	.
.	.	.	.	.	.	.	.	.
.	.	.	.	.	.	.	.	.
.	.	.	.	.	.	.	.	.
.	.	.	.	.	.	.	.	.
.	.	.	.	.	.	.	.	.
.	.	.	.	.	.	.	.	.
.	.	.	.	.	.	.	.	.
19.56	18.38	15.92	11.33	20.43	21.9	50.97	59.15	53.1
41.67	44.68	27.14	12.08	42.03	50.99	93.26	105.89	104.56
41.69	40.34	24.68	12.67	37.51	47.26	.	.	.
23.71	23.63	17.37	7.7	25.91	29.39	58.84	66.67	63.69
.	.	.	.	.	.	.	.	.
.	.	.	.	.	.	.	.	.
.	.	.	.	.	.	.	.	.
.	.	.	.	.	.	.	.	.
.	.	.	.	.	.	.	.	.
.	.	.	.	.	.	.	.	.
.	.	.	.	.	.	.	.	.
.	.	.	.	.	.	.	.	.
34.19	31.6	24.67	13.07	31.95	40.02	68.54	77.39	75.72
.	.	.	.	.	.	.	.	.
.	.	.	.	.	.	.	.	.
.	.	.	.	.	.	.	.	.
.	.	.	.	.	.	.	.	.
.	.	.	.	.	.	.	.	.
.	.	.	.	.	.	.	.	.
.	.	.	.	.	.	.	.	.
.	.	.	.	.	.	.	.	.
.	.	.	.	.	.	.	.	.
.	.	.	.	.	.	.	.	.
29.62	28.83	20.84	9.01	30.39	35.46	.	.	.
29.18	29.06	19.84	10.87	34.42	35.9	.	.	.
30.03	29.89	21.75	10.86	35.74	36.06	.	.	.
.	.	.	.	.	.	.	.	.
28.79	20.4	20.2	9.09	30.75	35.5	.	.	.
.	.	.	.	.	.	61.39	71.7	66.22
.	.	.	.	.	.	60.85	69.8	67.31
.	.	.	.	.	.	60.6	71.04	69.86
.	.	.	.	.	.	55.68	65.66	62.47
19.62	22.8	15.57	4.57	25.61	26.54	55.37		
33.98	36.31	22.75	11.7	33.9	40.4	81.91	95.76	91.2
30.31	31.65	18.91	9.14	34.41	36.84	77.82	86.8	75.35
40.32	34.68	20.7	12.6	34.76	37.41	75.25	84.85	82.69
30.22	34.14	19.32	12.75	31.28	37.49	75.31	84.75	82.29
34.1	34.8	20.13	13.67	35.88	41.75	86.42	96.34	93.1

31.81	35.04	20.2	9.3	33.73	38.99			
						79.91	88.87	87.76

C4	C5	C6	C7	C8	C9	M1	M 2	M3
9.83	6.2	11.62	11.09	4.11	5.61	48.7	9.27	8
3.11	1.74	4.8	3.01	1.81	1.32	21.68	2.08	2.44
7.69	4.42	8.41	5.42	3.23	3.96	10.82	5.33	6.37
8.69	6.36	8.07	7.79	3.24	3.89	52.26	4.68	6.27
8.33	4.39	8.94	7.86	3.45	3.17	56.21	5.33	6.92
6.72	3.66	6.03	5.89	2.15	3.67	48.63	4.41	5
6.12	3.52	6.75	6.42	1.33	2.29	43.46	4.9	5.52
6.58	3.52	6.75	5.85	2.03	2.68	48.43	5.38	5.31
6.28	3.75	5.87	5.95	2.84	3.74	42.37	4.57	4.26
6.18	3.59	6.86	6.94	2.94	2.91	38.13	4.1	4.95
6.31	4.58	5.6	5.84	2.58	3.66	31.56	4.2	4.22
5.86	3.76	7.32	6.84	3.02	2.29	44.36	4.5	5.23
6.59	3.39	7.83	6.25	3.66	3	44.81	4.24	5.03
5.82	3.24	6.95	6.23	2.49	3.61	42.24	4.49	4.95
12.2	12.05	15.28	13.76	7.71	4.96	52.63	6.66	10.02
.	.	.	.	.	.	.	.	.
9.68	8.23	12.62	15.16	5.02	4.13	37.12	5.58	7.21
9.15	8.87	9.42	11.58	5.32	4.29	36.26	5.09	7.2
9.81	9.54	13.85	12.46	6.15	5.82	41.72	4.89	8.17
9.56	8.84	11.29	11.79	4.41	7.9	42.33	5.17	8.12
7.81	8.16	9.95	12.36	4.01	4.37	36.24	5.25	7.45
10.97	9.58	13.89	16.63	5.26	4.89	47.6	6.45	9.05
10.02	8.59	14.35	17.95	3.16	4.06	40.47	5.72	8.37
9.86	7.99	11.4	10.08	4.64	4.7	47.51	6.22	7.63
8.68	7.53	8.7	10.67	3.65	5.75	47.22	6.33	6.91
.	.	.	.	.	.	.	.	.
11.88	7.85	10.9	13.29	5.58	6.12	61.14	8.78	8.9
9.3	7.51	9.17	11.42	5.54	7.45	31.72	6.83	6.47
6.76	4.04	8.5	7.73	2.31	2.38	.	.	.
6.35	4.21	6.3	6.09	2.79	3.62	53.27	4.27	4.2
6.25	3.67	7.45	6.77	2.92	3.63	42.64	4.63	5.22
6.83	3.99	8.01	7.83	2.84	3.04	61.8	4.48	5.39
7.83	4.89	8.73	8.54	3.48	2.78	64.58	5.68	6.58
13.09	8.16	14.59	13.81	5.72	9.39	97.37	10.77	10.02
22.02	10.19	19.81	18.96	8.03	8.42	127.61	12.07	14.17
10.13	6.18	11.26	12.02	3.78	4.6	77.75	7.23	8.59
8.22	5.82	9.16	10.19	3.48	3.75	58.6	6.35	7.53
24.94	14.83	25.44	24.46	10.98	12.57	175.16	16.88	17.61
20.47	11.39	20.6	19.27	7.59	8.29	148.99	14.52	13.81
26.16	15.2	27.84	24.43	10.24	10.92	164.16	14.6	19.62
19.98	11.72	20.51	19.24	8.66	9.52	144.56	17.14	13.96
16.33	6.75	16.31	17.57	6.88	7.44	169.37	16.77	19
8.03	6.13	8.93	8.84	3.64	5.02	57.82	5.73	6.46
13.6	7.67	15.8	14.88	7.16	7.33	103.62	12.5	10.99
20.4	13.43	22.99	21.2	7.07	10.01	134.05	14.46	15.84

18.74	12.43	21.01	21.71	8.45	9.19	128.58	13.49	15.5
14.99	9.78	16.03	13.69	5.16	5.33	89.59	11.29	10.64
19.56	9.5	20.46	19.8	7.16	7.43	142.94	13.66	13.82
21.63	9.2	21.67	21.01	7.68	8.99	169.08	14.88	18.52
24.95	11.22	23.72	25.33	15.57	10.37	177.54	17.53	18.02
16.33	6.75	16.31	17.57	7.44	6.88	161.09	15.81	16.48
35.72	19.76	34.44	.	.	.	200	23.1	24.55
35.3	22.33	38.24	35.24	13.75	13.86	.	.	.
33.55	21.26	36.57	31.96	14.76	15.62	.	.	.
.	.	.	.	.	.	.	.	.
34.94	23.57	35	34.98	9.61	14.23	188.34	28.46	24.96
38.69	24.58	37.06	36.18	13.31	14.7	207	24.73	24.63
9.36	6.33	9.74	10.68	3.05	3.63	59.91	6.83	6.98
7.42	5.67	8.55	8.99	3.68	3.29	55.68	5.7	6.27
11.63	5.38	10.04	11.48	4.11	3.77	76.93	8.33	9.22
8	4.95	9.24	8.35	3.65	4.52	55.34	5.15	6.56
.	.	.	.	.	.	.	.	.
.	.	.	.	.	.	.	.	.
7.1	5.09	8.97	8.1	2.41	2.33	49.25	5.11	5.99
10.44	7.64	10.99	10.23	3.94	7.72	56.32	5.55	7.96
7.71	5.25	8.8	8.22	5.12	4.78	57.08	5.35	6.55
10	6.72	11.17	10.73	4.55	3.16	69.49	8.43	8.83
7.89	6.64	9.85	9.21	2.94	3.24	39.42	5.46	6.71
7.06	6.89	8.25	9.86	2.03	3.17	41.81	5	6.31
10.02	7.41	8.99	9.52	3.87	9.11	55.41	5.69	6.61
7.81	4.61	9.25	8.57	3.9	4.08	52.45	5.22	6.06
13.24	8.59	13.39	15.52	5.29	5.4	84.38	8.32	9.55
13.15	8.14	13.15	13.59	5.77	5.63	73.91	10.37	8.35
32.78	23.18	39.8	33.59	13.99	14.86	118.58	19.94	23.08
34.09	24	36.86	37.53	16.04	13.01	201.52	22.74	23.06
21.76	12.8	22.97	23.46	8.47	9.01	103	17.16	20.91
32.41	23.06	33.6	29.05	12.46	14.37	121.24	19.68	22.26
.	.	.	.	.	.	.	.	.
.	.	.	.	.	.	.	.	.
.	.	.	.	.	.	.	.	.
.	.	.	.	.	.	.	.	.
.	.	.	.	.	.	.	.	.
.	.	.	.	.	.	.	.	.
.	.	.	.	.	.	.	.	.
25.78	13.76	25.58	27.22	12	11.45	.	.	.
25.28	16.97	25.47	30.58	10.56	8.83	.	.	.
.	.	.	.	.	.	156.63	17.82	20.04
.	.	.	.	.	.	160.59	19.64	19.81
.	.	.	.	.	.	163.23	18.56	20.98
.	.	.	.	.	.	150.84	16.14	20.95

.	.	.	.	.	.	.	.	.
.	.	.	.	.	.	.	.	.
.	.	.	.	.	.	.	.	.
.	.	.	.	.	.	.	.	.
.	.	.	.	.	.	.	.	.
.	.	.	.	.	.	.	.	.
.	.	.	.	.	.	.	.	.
.	.	.	.	.	.	.	.	.
.	.	.	.	.	.	.	.	.
.	.	.	.	.	.	.	.	.
17.45	10.9	19.57	20.65	8.14	8.22	90.88	12.38	13.96
38.37	26.5	43.58	44.87	9.22	14.87	201	37.9	29.48
.	.	.	.	.	.	.	.	.
26.14	16.27	24.98	29.1	7.81	4.69	.	.	.
.	.	.	.	.	.	135.83	17.63	16.75
.	.	.	.	.	.	.	.	.
.	.	.	.	.	.	.	.	.
.	.	.	.	.	.	.	.	.
.	.	.	.	.	.	.	.	.
.	.	.	.	.	.	148	21.3	15.77
.	.	.	.	.	.	.	.	.
.	.	.	.	.	.	.	.	.
30.87	19.81	33.9	32.38	11.16	13.02	135.04	23.77	20.3
.	.	.	.	.	.	.	.	.
.	.	.	.	.	.	.	.	.
.	.	.	.	.	.	.	.	.
.	.	.	.	.	.	.	.	.
.	.	.	.	.	.	.	.	.
.	.	.	.	.	.	.	.	.
.	.	.	.	.	.	.	.	.
.	.	.	.	.	.	.	.	.
.	.	.	.	.	.	.	.	.
.	.	.	.	.	.	.	.	.
.	.	.	.	.	.	.	.	.
.	.	.	.	.	.	125.87	19.64	21.87
.	.	.	.	.	.	131.41	24.66	20.08
.	.	.	.	.	.	132.21	24.15	19.49
.	.	.	.	.	.	.	.	.
31.14	22.79	30.79	30.8	13.21	8.5	.	.	.
28.9	21.39	30.6	29.46	13.67	8.08	.	.	.
28.11	21.31	29.13	28	12.49	9.36	.	.	.
25.18	18.16	26.98	26.25	11.93	9.81	.	.	.
23.17	19.42	23.75	23.48	8.61	19.91	133.18	20.04	14.96
34.57	22.53	37.18	42.23	10.96	12.51	174.93	29.48	23.8
28.47	21.91	31.28				161.54	27.26	20.35
30.08	22.92	36.6	38.96	9.01	12.07	162.86	29.03	20.79
29.55	21.18	36.11	38.55	9.16	12.13	162.33	24.9	20.63
35.29	21.3	38.41	41.69	10.39	10.93	183.72	31.8	22.03

						154.93	24.55	20.46
30.57	18.81	30.52	32.82	12.61	10.89			

M4	M5	M5 1	M5 2	M5 3	Ph1 1	Ph1 2	Ph2 1	Ph2 2	Ph3 1
11.7	12.9	42.1	6.2	5	20.5	5.5	13.1	8.3	12.1
3.4	4.07	20.06	2.08	2.26	10.81	3.9	6.26	3.14	5.08
9.67	9.2	.	.	.	.	.	.	.	.
11.17	10.79	43.39	4.24	3.27	27.92	9.72	14.57	8.14	8.98
10.02	9.15	43.48	3.68	3.53	27.94	5.7	14.96	6	17.94
10.29	7.6	42.42	3.2	2.47	25.29	4.35	11.06	4.35	12.66
9.78	7.4	36.45	3.35	2.53	19.41	4.2	11.71	4.15	13.67
7.53	6.52	44.7	3.57	2.72	23.1	4.44	11.84	4.32	13.8
9.29	6.31	37.46	3.21	2.22	19.55	4.37	11.96	3.56	13.26
8.12	7.2	33.27	3.24	3.54	18.8	4.52	12.07	4.36	13.5
6.82	6.92	28.38	3.19	2.76	16.5	3.7	10.23	3.88	10.28
9.54	6.9	39.19	3.52	3.11	20.1	4.17	10	4.5	16
7.71	7.14	37.83	2.55	3.09	19.26	3.84	11.98	3.91	12.2
9.17	7.16	38.48	3.9	2.48	19.8	4.42	12.01	4.06	11.39
10.41	14.07	45.28	6.31	7.88	24.26	9.76	16.33	9.45	26.42
.	.	.	.	.	.	.	.	.	.
11.43	10.28	35.06	5.39	6.25	14.92	7.64	10.89	6.46	26.36
9.52	9.47	34.27	5.16	6.44	15	7.31	12	9	26
12.22	12	39.67	5.21	5.64	19.34	6.57	15.26	6.4	15.17
12.75	12.3	40.67	5.3	5.56	21.65	7.5	14.57	8.3	15
10.8	10.37	33.59	5.91	6.58	15.59	7.26	12.21	6.43	21.91
13.23	12.19	41.51	6.3	7.08	24.4	9.15	14.85	9.23	19.53
11.93	11.22	38.2	6.03	6.51	.	.	.	.	.
11.83	9.5	39.92	5.84	5.77	18.4	6.77	8.81	7.71	.
10.51	9.12	47.05	5.9	4.37	19.43	6.48	9.67	8.71	12.98
.	.	.	.	.	.	.	.	.	.
14.31	11.98	56.39	7.41	5.74	23.57	8.3	15.33	8.03	25.65
12.71	10.57	25	5	5	21.39	6.76	10	8.5	13.5
.	.	.	.	.	.	.	.	.	.
9.81	6.47	53	4	2.1	18.88	3.95	11.17	3.62	20.1
7.97	7.44	37.66	3.27	2.56	19.57	4.45	11.59	4.23	20.1
8.87	7.68	55.54	3.88	2.25	20.17	5.24	10.81	4.81	16.7
9.5	8.23	58.54	6.29	4.3	21.84	6.17	12.61	6.95	17.52
15.94	17.1	88.42	8.1	6.1	28.01	15.28	19.98	11.72	24.65
21.31	23.42	115.71	12.9	5.02	40.49	22.1	25.32	17.9	33.06
14.95	12.23	67.46	7.51	4.35	22.35	7.1	14.5	6.72	22
11.62	9.67	54.31	5.15	3.88	22.04	6.17	14.84	6.06	25
29.06	24.42	154.07	11.61	8.24	45.62	16.3	31.1	16.34	28
22.75	19.73	132.91	10.1	5.89	40.15	13.58	26.86	13.08	27
28.57	30.05	143.2	15.02	11.12	46.58	22.58	31.7	23.8	34.17
21.45	20.13	128.34	11.99	8.32	45.09	13.31	32.63	12.37	33
27.47	23.73	144.72	16.1	8.37	48.9	16.43	33.61	16.08	44.4
9.86	9.64	50.62	5.6	4.63	22.4	5.91	13.42	5.46	25
20.08	17.6	92.1	7.85	5.15	32.12	17.22	21.01	12.68	29.88
24.97	22.81	120.06	9.7	7.7	42.97	12.01	23.62	12.22	27.13

23.91	21.43	115.53	10.1	7.41	40.1	12.82	23.85	14.67	30
15.66	13.73	78.24	7.44	6.29	28.12	13.14	19.47	12.54	25.16
23.09	18.86	127.66	8.8	4.8	40.29	11.21	22.13	11.5	28.41
27.45	22.96	152.94	16.14	12.06	51.72	21.04	27.85	13.42	39.47
28.08	22.3	154	17	13	45.52	15.38	26.69	14.17	30.54
25.81	22.66	151	16	11.5	41.23	13.48	23.26	13.86	27.22
35.45	42.17	177.77	19.43	13.62	54.27	21.07	41.08	20.92	45.72
.	.	.	21.17	14.02	.	.	.	.	.
.	.	.	.	.	.	.	.	.	.
.	.	.	.	.	.	.	.	.	.
36.54	37.37	162.61	23.08	15.05	57.63	35.7	41.57	29.89	44.44
34.91	31.88	173.53	22.94	13.28	64.22	23.99	44.5	23.71	38.75
11.47	9.95	54.27	3.84	2.28	25.42	5.95	16	5.8	16
11.5	9.85	52.52	3.04	1.5	24.86	5.86	15.11	5.34	14.29
15.07	12.49	69.38	3.66	1.4	33.29	7.49	20.17	7.77	24
9.99	9.28	46.41	5.51	3.29	23.11	5.99	16.3	5.08	10.92
.	.	.	.	.	.	.	.	.	.
.	.	.	.	.	.	.	.	.	.
10.4	8.49	40.64	4.76	3.2	20.79	5.41	14.84	4.07	9.5
10.91	7.3	49.63	5.49	4.35	25.38	6.18	18.16	5.19	14
11.92	11.64	50.62	3.98	3.22	24.53	6.12	15.83	4.75	9.78
11.46	12.1	58.07	5.63	3.84	28.28	7.1	19.21	6.03	15
9.44	7.73	32.89	5.53	5.02	18.07	5.59	10.21	5.65	15.85
9.85	9.33	35.9	5.01	5.03	18.51	8.78	11.21	7.75	13.38
12.19	11.86	48.72	6.13	4.81	20.11	6.35	12.51	6.21	15
11.46	8.85	45.56	4.74	3.77	18.47	8.47	11.42	4.85	13.72
15.16	15.35	70.71	8.89	5.86	32.06	14.6	18.2	12.81	25.52
14.55	13.07	67.5	8.25	6.52	31.74	8.86	15.16	8.07	22.32
28.82	31.9	99.69	20.69	14.04	46.79	24.26	30.01	23.71	42.27
32.74	29	175.49	22.35	13.07	59.16	24.05	40.13	24.29	45.5
26.61	26.49	.	.	.	35.73	22.32	22.99	20.28	.
39.11	38.08	94.23	17.84	11.55	47.36	19	29.27	27.47	41.06
.	.	.	.	.	.	.	.	.	.
.	.	.	.	.	.	.	.	.	.
.	.	.	.	.	.	.	.	.	.
.	.	.	.	.	.	.	.	.	.
.	.	.	.	.	.	.	.	.	.
.	.	.	.	.	.	.	.	.	.
.	.	.	.	.	.	.	.	.	.
.	.	.	.	.	.	.	.	.	.
.	.	.	.	.	.	.	.	.	.
29.06	27.07	.	.	.	.	.	.	.	.
31.56	30.54	.	.	.	.	.	.	.	.
33.42	29.12	.	.	.	.	.	.	.	.
29.83	28.41	.	.	.	.	.	.	.	.

.	.	135.38	19.77	12.89	.	.	.	.	.
.	.	.	.	.	53.6	17.91	.	.	.
.	.	.	.	.	52.85	17.61	.	.	.
.	.	.	.	.	50.52	14.94	.	.	.
.	.	.	.	.	51.97	14.45	.	.	.
.	.	.	.	.	56.62	17.91	.	.	.
.	.	.	.	.	.	.	31.64	20.02	.
.	.	.	.	.	.	.	32.14	21.4	.
.	.	.	.	.	.	.	30.74	18.75	.
.	.	.	.	.	.	.	30.1	18.77	.
21.22	18.18				33.06	10.84	24.04	13.67	19.34
40.98	41.44	109.12	17.87	14.97	55.07	22.16	33.44	24.22	22.68
.	.	.	.	.	.	.	.	.	.
.	.	.	.	.	.	.	.	.	.
25.79	26.31	.	.	.	38.52	16.78	35.19	17.76	30.67
.	.	.	.	.	.	.	.	.	.
.	.	.	.	.	.	.	.	.	.
.	.	.	.	.	.	.	.	.	.
.	.	.	.	.	.	.	.	.	.
24.75	27.67	.	.	.	.	.	.	.	.
.	.	.	.	.	.	.	.	.	.
.	.	.	.	.	.	.	.	.	.
35.41	33.44	44.32	7.95	4.71	52.78	21.63	38.7	21.4	41.46
.	.	.	.	.	.	.	.	.	.
.	.	.	.	.	.	.	.	.	.
.	.	.	.	.	.	.	.	.	.
.	.	.	.	.	.	.	.	.	.
.	.	.	.	.	.	.	.	.	.
.	.	.	.	.	.	.	.	.	.
.	.	.	.	.	.	.	.	.	.
.	.	.	.	.	.	.	.	.	.
.	.	.	.	.	.	.	.	.	.
.	.	.	.	.	.	.	.	.	.
.	.	.	.	.	.	.	.	.	.
32.3	31.84	.	.	.	.	.	.	.	.
28.86	34.79	.	.	.	.	.	.	.	.
32.25	35.69	.	.	.	.	.	.	.	.
.	.	.	.	.	.	.	.	.	.
.	.	.	.	.	.	.	.	.	.
.	.	.	.	.	.	.	.	.	.
.	.	.	.	.	.	.	.	.	.
.	.	.	.	.	.	.	.	.	.
23.2	27.42				41.24	26.8	37.07	26.02	32.08
40.1	37.79	109.12	17.87	14.97	58.82	19.75	54.6	18.7	46.5
34.45	32.07	86.88	17.92	13.65	53.46	18.25	50.8	17.78	40.32
36.3	34.48	109	17.87	14.97	54.56	18.74	50.68	18.09	42.25
35.02	34.13	109.12	17.87	14.97	53.42	18.46	50.33	16.98	43.26
38.14	40.37	61.17	21.13	16	61.04	20.69	56.16	19.57	47.35

34.06	34.8				51.97	18.68	47.25	18.51	41.67

Ph3 2

7.22
2.51
.
4.09
6.72
5.5
5.09
5.1
2.53
5.5
4.66
3
2.41
5.44
7.41
.
6.46
9
7.75
9
5.77
7.97
.
.
3.76
.
11
4
.
6.18
6.18
3.33
4.12
11.16
7.07
7.97
12
8
7
17.18
10
19.79
12
6.23
7.08

8.5
12.24
7.17
9.57
15.12
15.51
20.55
.
.
.
22.29
21.52
5.5
4.11
6
5.51
.
.
5
4
2.76
5
4.27
7.1

5.5
6.11
11.86
11.56
25.81
26.6
19.09
16.52
.
.
.
.
.
.
.
.
.
.
.
.
.

.
.
.
.
.
.
.
.
.
.
9.9
61.88
.
.
15.52
.
.
.
.
.
.
.
25.39
.
.
.
.
.
.
.
.
.
.
.
.
.
.
.
.
.
.
.
16.82
18.91

21.25
16.93
17.89
19.94

16.32
