# Supplementary material for: Locomotion in Extinct Giant Kangaroos: Were Sthenurines Hop-Less Monsters?
Source: PLoS One. 2014 Oct 15;9(10):e109888. doi: 10.1371/journal.pone.0109888 (PMC4198187; doi:10.1371/journal.pone.0109888)
Supplement: Table S6 — Additional data on the calcaneum. (PDF) [file pone.0109888.s011.pdf]

TABLE 6. ADDITIONAL DATA ON THE CALCANEUM

FAMILY	SUBFAMILY	GENUS	
Balbaridae		Nambaroo gillespieae	
Hypsiprymnodontidae		Hypsimprymnodon moschatus	
Macropodidae	incertae sedis	Ngamaroo archeri	
Macropodidae	Potoroinae	Aepyprymnus rufescens	1
Macropodidae	Potoroinae	Aepyprymnus rufescens	1
Macropodidae	Potoroinae	Bettongia giamardi cuniculus	1
Macropodidae	Potoroinae	Bettongia pencillata	1
Macropodidae	Potoroinae	Potorous tridactylus	1
Macropodidae	Potoroinae	Potorous tridactylus	1
Macropodidae	Lagostrophinae	Lagostrophus fasciatus	1
Macropodidae	Macropodinae	Dendrolagus bennettianus	1
Macropodidae	Macropodinae	Dendrolagus dorianus	1
Macropodidae	Macropodinae	Dendrolagus scottae	1
Macropodidae	Macropodinae	Dendolagus lumholtzi	1
Macropodidae	Macropodinae	Dendolagus lumholtzi	1
Macropodidae	Macropodinae	Dendrolagus matschiei	1
Macropodidae	Macropodinae	Dorcopsis luctosa	1
Macropodidae	Macropodinae	Dorcopsis muelleri	1
Macropodidae	Macropodinae	Dorcopsulus vanheuri	1
Macropodidae	Macropodinae	Lagorchestes hirsutus	1
Macropodidae	Macropodinae	Lagorchestes conspicilatus	1
Macropodidae	Macropodinae	Macropus agilis	1
Macropodidae	Macropodinae	Macropus agilis	1
Macropodidae	Macropodinae	Macropus eugenii	1
Macropodidae	Macropodinae	Macropus irma	1
Macropodidae	Macropodinae	Macropus fuliginosis	1
Macropodidae	Macropodinae	Macropus giganteus1	1
Macropodidae	Macropodinae	Macropus giganteus2	1
Macropodidae	Macropodinae	Macropus giganteus3	1
Macropodidae	Macropodinae	Macropus giganteus4	1
Macropodidae	Macropodinae	Macropus robustus	1
Macropodidae	Macropodinae	Macropus rufogriseus1	1
Macropodidae	Macropodinae	Macropus rufogriseus2	1
Macropodidae	Macropodinae	Macropus rufus1	1
Macropodidae	Macropodinae	Macropus rufus2	1
Macropodidae	Macropodinae	Macropus rufus3	1
Macropodidae	Macropodinae	Macropus ferragus	1
Macropodidae	Macropodinae	Onychogalea freanata	1
Macropodidae	Macropodinae	Onychogalea freanata	1
Macropodidae	Macropodinae	Petrogale assimilis	1
Macropodidae	Macropodinae	Petrogale lateralis	1
Macropodidae	Macropodinae	Petrogale penIcillata	1
Macropodidae	Macropodinae	Setonix brachyurus	1
Macropodidae	Macropodinae	Thylogale billardierii	1
Macropodidae	Macropodinae	Thylogale stigmatica	1
Macropodidae	Macropodinae	Thylogale thetis	1

Macropodidae	Macropodinae	Wallabia bicolor	1
Macropodidae	Macropodinae	Dorcopsoides	44
Macropodidae	Macropodinae	Dorcopsoides	
Macropodidae	Macropodinae	Dorcopsoides	
Macropodidae	Macropodinae	Dorcopsoides	
Macropodidae	Macropodinae	Protemnodon anak	
Macropodidae	Macropodinae	Protemnodon cf brevus	
Macropodidae	Macropodinae	Protemnodon snewini	
Macropodidae	Macropodinae	Protemnodon sp1	
Macropodidae	Macropodinae	Protemnodon sp2	
Macropodidae	Macropodinae	Protemnodon sp3	
Macropodidae	Macropodinae	Protemnodon sp4	
Macropodidae	Stenurinae	Archaeosimus cegsai	
Macropodidae	Stenurinae	Hadronomas puckridgi	
Macropodidae	Stenurinae	Hadronomas puckridgi	
Macropodidae	Stenurinae	Hadronomas puckridgi	
Macropodidae	Stenurinae	Rhizosthenurus flanneryi	
Macropodidae	Stenurinae	Procoptodon sp.1	
Macropodidae	Stenurinae	Procoptodon sp.2	
Macropodidae	Stenurinae	Simosthenurus occidentalis	
Macropodidae	Stenurinae	Sthenurus stirlingi	
Macropodidae	Stenurinae	Sthenurus stirlingi	
Macropodidae	Stenurinae	Sthenurus stirlingi	
Macropodidae	Stenurinae	Sthenurus tindalai	

SPECIMEN NUMBER	C1	C2	C3	C4	C5	C6	C7
QM F35432	30.35	32.07	30.15	9.83	6.2	11.62	11.09
SAM M11940	8.7	10.92	10.1	3.11	1.74	4.8	3.01
SAM P23821	18.45	19.5	18.88	7.69	4.42	8.41	5.42
QM JM5580	22.22	23.65	22.53	8.33	4.39	8.94	7.86
AMNH 22788	21.29	22.32	21.34	7.85	3.88	7.5	7.18
AMNH 65268	17.41	19.39	18.07	6.53	3.35	7.84	6.83
SAM M22661	15.51	17.9	16.51	5.7	3.27	6.25	5.27
AMNH 65309	16.05	16.52	15.84	5.58	3.16	5.74	6.3
SAM M7381	15.2	16.16	15.16	5.41	3.18	6.32	5.59
AM M40303	20.07	21.04	19.26	5.82	3.24	6.95	6.23
SAM M5530	33.28	35.85	34.13	12.85	10.56	16.74	13.08
AM M9109	27.29	30.18	27.04	9.68	8.23	12.62	15.16
AM M24424	29.47	32.52	29.67	10.02	8.59	14.35	17.95
AMNH 65258	31.05	33.92	31.72	9.81	9.54	13.85	12.46
AMNH 65263	26.43	29.07	27.24	10.45	8.98	13.21	11.35
AMNH 194793	19.37	21.24	19.53	6.93	5.76	9.45	8.3
SAM M15178	24.48	26.26	25.55	8.68	7.53	8.7	10.67
AM M32341	35.78	37.51	34.6	11.88	7.85	10.9	13.29
AMNH 194790	19.72	21.61	19.7	6.76	4.04	8.5	7.73
AM M40038	18.47	21.12	19.35	6.25	3.67	7.45	6.77
AMNH 197659	23.95	27.49	25.14	7.83	4.69	8.06	8.39
AMNH 184582	49.43	54.56	50.38	15.16	8.47	19.29	15.84
AMNH 35621	51.9	55.8	54.04	17.66	11.27	18.05	18.39
AMNH 193947	28.98	33.17	30.97	10.02	6.14	10.35	10.6
AMNH 150319	35.93	40.78	37.79	10.65	6.64	11.55	10.95
AMNH 200826	76.63	82.69	78.47	27.59	13.8	24.58	24.36
QM J11525	78.427	87.71	82.11	25.9	14.74	26.41	26.19
AMNH 90136	75.15	84.37	78.16	24.26	13.42	25.34	24.81
AMNH 35747	74.25	81.46	78.45	26.16	15.2	27.84	24.43
AMNH 42905	11.58	68.28	65.58	62.47	10.83	18.46	20.18
AMNH 65029	46.54	50.52	48.44	12.922	8.52	14.7	14.45
AMNH 14154	41.8	45.06	42.94	13.76	8.16	15.37	14.14
AMNH 273247	41.24	46.33	42.89	13.17	6.36	15.15	14.38
AMNH 70284	85.21	93.16	89.01	24.95	11.22	23.72	25.33
AMNH 200473	70.3	79	73.9	21.63	9.2	21.67	21.01
QM J22115	55.34	62.48	57.75	16.33	6.75	16.31	17.57
SAM P43040	94.09	103.32	99.5	34.38	21.91	29.1	32.03
AMNH 42959	25.01	26.64	25.06	7.42	5.67	8.55	8.99
NMV C6500	27.06	30.5	28.06	9.36	6.33	9.74	10.68
QM J4470	25.09	27.79	26.44	8	4.95	9.24	8.35
AM M24183	24.84	26.99	24.94	6.93	5.05	8.97	8.16
AMNH 35758	28.46	29.93	28.91	7.63	5.85	9.74	10.31
AMNH 196399	24.65	26.74	25.04	7.4	5.72	8.28	8.24
AMNH 65215	31.13	32.82	31.29	10.69	6.44	13.06	12.81
AMNH 65140	22.71	25.49	24.3	7.98	4.95	8.37	8.74
AM M51512	24.71	26.33	24.54	7.81	4.61	9.25	8.57

AMNH 65125	43.93	47.98	44.34	14.47	9.62	13.86	16.22
NT P890	38.46	42.53	39.21	13.15	8.14	13.15	13.55
NT P648-7	34.55	39.72	36.82	12.56	7.51	12.66	12.87
NT P878-6	37.91	41.61	37.82	13.05	7.85	12.96	13.74
NT unnumbered	38.58	42.06	40.42	13.53	7.25	11.55	10.93
QM F14675	103.02	113.77	108.56	34.09	24	36.86	37.53
SAM P20810	91.95	100.65	91.11	37.15	26.33	43.6	37.63
QM F9075/9076	74.08	78.47	75.36	21.76	12.8	22.97	23.46
AMNH/SIAM 37 (smaller)	85.75	96.55	90.53	33.38	11.8	32.35	29.28
AMNH/SIAM 37 (larger)	87.1	95.04	91.01	33.54	17.69	38.15	33.61
FU 1661	83.51	97.34	94.45	35.45	31.92	40.24	37.43
FU 40-01-19	87.47	95.05	89	38.02	22.34	37.33	34.71
FU Unnumbered	93.27	106.02	100.99	39.03	27.12	43.88	45.6
NT MPUD-07/72	70.94	79.69	75.42	25.78	15.76	25.58	27.22
NT P9336	71.09	78.16	75.67	25.28	16.97	25.47	30.58
NT P8745-2	85.27	93.01	86.27	30.43	20.24	36.67	36.39
QM F31456	50.97	59.15	53.1	17.45	10.9	19.57	20.65
AMNH 19272	87.57	99.6	95.13	36.85	22.7	35.4	41.24
AMNH 11749	76.33	86.09	82.37	32.22	19.93	36.41	38.54
SAM P20820	68.54	77.39	75.77	30.87	19.81	33.9	32.38
AMNH 17494A	86.42	96.34	93.1	35.29	21.3	38.41	41.69
AMNH/ SIAM 81	78	85.69	82.47	30.51	18.07	35.56	33.77
AMMH 117473	77.26	85.16	82.86	29.76	17.84	31.6	33.01
AMNH 117493	79.91	88.87	87.76	30.57	18.81	30.52	32.82

C8	C9	C10	C11	C12	C13	C14	C15	C16
4.11	5.61	12.94	6.48	25.99	14.11	14.9	7.73	7.41
1.81	1.32	6.92	3.69	7.67	4.84	5.35	3.13	1.92
3.23	3.96	10.82	4.39	13.12	10.08	10.91	6.63	5.03
3.17	3.45	10.64	5.58	19.92	11.05	11.71	6.49	4.42
3.68	3.68	9.77	5.76	17.67	10.11	11.57	6.55	4.01
2.34	2.32	8.16	3.88	14.54	8.31	9.4	5.47	4.14
2.7	3	7.22	3.58	13.96	7.92	8.35	4.49	3.42
2.06	2.58	7.19	4.55	13.65	8.17	8.41	4.42	4.08
2.71	2.25	6.9	3.39	13.15	8.022	9.16	4.69	3.21
3.01	2.49	8.45	5.86	15.6	8.67	10.46	5.22	4.43
4.54	5.87	16.78	8.31	29.65	21.3	23.97	11.34	12.93
4.13	5.02	14.34	7.17	22.15	17.33	18.95	9.08	12.2
4.06	3.16	15.71	7.61	24.57	18.27	20.53	9.4	10.46
6.15	5.82	26.61	9.72	31.53	18.32	22.06	10.75	9.61
3.48	4.6	14.19	8.9	19.8	16.72	17.81	8.61	9.91
3.51	4.1	11.17	6.47	16.07	14.03	14.84	7.85	7.24
3.65	5.75	11.01	6.17	21.16	13.45	16.25	6.26	8.35
5.58	6.12	14.18	7.68	29.98	15.88	19.87	7.89	11.38
2.31	2.38	8.61	5.2	15.51	9.34	10.81	4.95	4.97
3.63	2.92	8.45	3.89	15.76	10.1	10.76	5.05	5.56
3.8	3.85	9.56	4.63	20.07	10.28	12.09	4.8	5.18
7.32	6.94	16.62	9.33	39.98	19.97	22.6	9.33	13.74
7.4	8.76	48.7	11.64	44.24	23.53	29.1	11.22	16.36
4	4.45	12.99	6.25	24.11	14.3	16.09	5.79	7.52
4.77	4.77	15.29	7	29.59	15.57	16.98	7.5	7.98
10.95	11.35	28.62	17.34	61.54	30.38	34.38	14.83	18.09
12.32	11.33	28.17	11.95	75.58	30.32	35.03	13.65	17.45
10.4	10.77	28.3	16.8	64.15	28.78	33.64	13.5	18.72
10.24	10.92	27.43	15.55	64.69	30.16	34.89	12.96	18.91
19.02	7.52	21.5	10.99	54.13	24.67	27.11	23.67	13.41
5.92	5.45	18.72	10.21	36.99	19.44	23.35	10.22	9.35
6.4	7.38	15.31	8.96	34.77	18.29	21.2	8.37	9.7
7.35	7.04	17.54	9.16	35.66	20.25	22.1	9.18	8.97
15.57	10.37	31.21	19.74	75.75	31.69	34	14.4	15.63
7.68	8.99	28.8	21	62.11	26.34	32.15	13.14	13.49
6.88	7.44	24.74	18.13	47.8	21.97	23.62	11.41	9.28
11.4	10.5	33.33	21.56	81.12	41.8	42.32	16.69	28.07
3.68	3.29	10.69	6.43	19.66	12.83	13.73	5.22	7.07
3.05	3.63	12.08	5.83	22.9	14.17	15.62	5.84	7.24
4.52	3.65	11.13	5.81	20.95	11.08	12.39	5.5	6.5
2.33	2.41	8.67	3.86	20.88	10.32	12.37	5.09	5.85
3.68	4.1	13.09	6.57	22.55	12.39	12.84	5.73	7.83
3.27	2.95	10.86	5.86	20.66	10.91	12.33	5.94	6.3
4.48	5.38	13.57	7.5	23.55	15.62	16.84	6.86	7.78
3.45	3.63	10.36	5.45	19.38	11.45	12.41	6.26	6.2
4.08	3.96	10.69	6.41	19.81	11.92	13.31	6.89	6.07

6.13	6.25	17.94	10.35	36.67	19.6	20.72	9.38	10.76
8.63	5.77	16.59	11.75	33.56	17.88	20.81	6.97	8.73
4.91	5.5	15.44	9.02	32.45	17.26	18.01	8.24	9.09
5.96	5.7	16.61	8.73	32.84	17.72	18.88	8.67	8.66
2.99	5.66	16.37	6.79	33.91	17.71	19.7	8.68	10.23
16.04	13.01	38.75	20.89	92.95	39.7	43.99	19.52	33.86
12.7	14.88	38.94	19.37	80.86	48.32	54.28	25.97	32.94
9.01	8.47	28.32	18.72	61.76	28.8	31.75	15.04	18.05
12.61	11.59	33.7	22.58	75.46	33.45	40.34	18.63	20.31
12.41	13.87	35.83	22.78	74.35	38.47	47.21	32.41	24.77
10.68	16.66	39.92	24.75	80.38	43.34	50.16	22.69	33.27
12.26	14.71	36.38	18.5	77.25	45.82	49.92	24.13	30.04
15.06	20.28	35.74	19.24	86.44	47.32	50.39	28.31	34.8
11.45	12	29.29	18.03	63.89	29.85	34.11	15.1	20.05
8.83	10.56	30.13	20.01	64.79	34.72	36.71	18.84	17.34
12.57	12.04	34.11	21.53	70.47	37.01	43.38	18.76	22.76
8.14	8.22	21.55	14.82	43.91	24.75	28.53	12.09	15.23
12.55	10.5	33.29	20.86	77.49	39.99	47.6	31.96	34.3
11.56	9.39	28.99	16.36	67.77	34	40.41	24.12	30.97
13.02	11.16	31	15.01	60.5	34.64	37.95	19.42	22.37
10.39	10.93	31.33	19.19	75.23	36.6	45.12	31.76	33.42
11.27	10.18	29.85	14.09	66.96	37.07	39.33	22.83	27.01
9.64	8.97	28.34	17.25	67.19	35.05	37.53	27.46	25.59
12.61	10.89	29.71	21.69	74.27	34.11	38.38	30.11	30.13

C17	C18	C19	C20	C21	C22	C23	C24	C25
4.13	7.02	8.38	13.12	10.46	15.12	5.71	7.42	23.78
2.02	2.21	4.85	3.96	3.51	4.3	2.11	2.38	6.53
2.32	5.5	9.1	9.2	6.92	11.45	3.9	5.82	18.45
5.36	4.2	8.81	9.46	8.12	10.34	4.61	5.6	15.68
5.75	4.84	7.13	9.65	7.89	11.66	4.78	5.68	16.12
3.77	3.81	6.68	7.66	6.5	8.73	3.26	3.77	13.47
3.68	2.78	13.35	6.69	5.79	8.6	3.1	7.18	3.13
3.32	3.74	6.15	6.56	5.24	7.81	2.36	2.61	12.07
2.43	3.85	6.68	6.37	5.05	8.22	2.82	3.32	11.4
3.87	4.05	7.05	7.29	5.71	8.13	3.19	3.7	15.58
3.13	7.32	12.71	15.16	9.93	19.84	5.82	7.46	26.42
6.19	7.32	12.45	12.62	8.49	17.11	6.23	6.8	22
6.98	8.64	12.19	11.95	9.04	16.9	5.78	6	21.07
3.23	7.68	9.82	15.09	9.46	17.14	6.23	6.89	30.5
4.05	7.27	10.02	12.16	8.54	15.02	7.45	6.23	20.42
5.22	4.66	9.08	10.4	7.35	13.59	5.04	5.31	13.39
4.41	6.31	9.52	10.59	8.8	11.63	4.65	5.98	22.67
5.91	7.67	13.98	13.52	10.87	17.84	6.39	6.8	30.68
3.68	4.22	6.64	7.89	6.68	9.77	3.43	3.44	15.85
3.19	5.19	8	7.72	5.86	9.11	3.06	3.9	15.82
6.13	5.12	7.47	9.63	8.32	10.48	4.02	4.81	21.48
8.57	8.66	19.22	16.54	15.56	18.11	7.96	7.54	42.05
8	8.57	21.48	19.26	17.13	20.65	9.75	10.85	46.52
5.39	5	10	11.5	9.67	11.99	5.19	6.05	26.92
6.25	5.81	13.09	13.14	11.13	14.26	5.19	34.71	6.59
12.95	11.32	30.81	27.99	24.16	32.34	12.89	14.7	71.15
12.13	9.69	30.44	27.32	23.83	28.48	14.14	15	75.61
13.46	13.32	27.7	28.66	24.37	29.05	14.03	16.18	71.97
14.26	10.06	27.31	25.45	23.63	29.89	11.62	13.44	67.25
10.73	8.02	22.5	22.74	18.16	8.12	22.5	11.18	61.55
7.84	6.07	19.2	17.8	15.63	19.98	6.57	9.04	44.55
7.78	7.32	17.64	16.75	14.24	19.63	6.89	6.92	38.53
8.71	7.42	16.13	16.65	13.26	17.24	7.4	8	36.78
15.22	7.37	33.33	27.7	24.9	27.3	13.41	12.29	85.05
12.33	8.6	27.93	25.89	23.76	27.1	11.54	13.58	67.05
11.99	7.27	22.88	20.74	19.5	20.33	10.5	9.24	52.98
13.54	9.56	34.47	33.26	29.4	34.12	15.2	20.26	88.89
5.16	4.9	9.04	10.44	9.35	11.25	4.59	7.14	22.63
6.1	4.87	10.17	11.33	9.86	13.97	5.46	7.15	25.19
4.31	4.9	12.68	9.58	7.57	11.58	4.37	5.5	24.65
4.18	3.97	10.6	9	7.05	10.15	3.91	4.98	23.85
4.49	5.07	9.96	10.7	8.5	12.17	4.76	6.19	26.31
3.92	5.51	7.81	9.38	7.26	10.5	4.78	4.7	22.54
5.08	6.7	9.77	13.48	10.67	15.89	5.58	6.03	28.57
4.81	5.17	10.63	9.43	8.3	11.09	4.56	4.22	22.21
6.98	5.53	10.21	10.21	8.41	11.02	4.03	4.9	21.6

6.39	8.8	21.39	16.82	14.3	19.22	7.16	7.93	38.98
6.73	7.73	14.33	14.26	12.87	15.11	6.11	7.3	36.58
6.68	7.01	13.89	14.39	11.93	16.66	5.43	7.5	31.95
6.72	8.05	14.7	13.93	12.94	16.68	6.41	6.8	35.88
6.72	8.18	14.19	14.12	12.44	15.79	6.22	7.23	32.45
19.69	15.52	40.33	36.07	31.81	40.69	17.58	17.2	100.65
10.98	16.64	41.53	41.02	32.17	51.1	17.05	19.61	84.38
12.95	9.48	28.92	25.3	27.33	27.75	13.28	12	65.18
14.23	12.59	32.9	31.3	29.22	31.2	15.53	15.57	80.56
16.18	15.89	36.24	36.39	31.55	44.76	18.74	17.25	84.28
10.89	14.75	32.06	38.05	28.23	46.19	17.93	18.06	81.26
11.43	13.29	42.21	37.32	31.84	45.19	16.16	17.31	82.77
15.52	15.5	45.33	43.48	40.49	44.94	21.15	25.23	91.76
14.51	9.68	30.88	31.27	26.75	35.88	13.76	15.1	65.69
14.07	10.77	31.62	31.3	25.66	36	12.63	15	65.05
15.41	10.75	32.12	36.38	30.13	38.08	15.27	17.28	84.06
7.76	8.85	16.42	22.72	19.05	26.11	8.75	11.9	43.77
13.79	13.16	35.22	38.38	36.22	42.24	22.7	22.2	78.18
10.73	9.61	28.8	33.67	30.09	37.74	16.24	16.5	69.07
11.07	11.26	37.31	31.49	32.59	34.64	13.46	15.41	62.63
17.22	14.89	32.48	39.39	35.84	43.99	20.97	24.16	81.89
11.75	10.29	28.31	30.16	29.15	33.63	18.9	19.55	71.84
12.89	11.36	29.29	29.95	30.36	34	16.75	21.05	69.41
14.25	13.82	34.02	34.59	33.01	38.96	20.04	17.25	84.28
