# Supplementary material for: Locomotion in Extinct Giant Kangaroos: Were Sthenurines Hop-Less Monsters?
Source: PLoS One. 2014 Oct 15;9(10):e109888. doi: 10.1371/journal.pone.0109888 (PMC4198187; doi:10.1371/journal.pone.0109888)
Supplement: Text S1 — Description of bivariate plots. (DOC) [file pone.0109888.s012.doc]

**Description of Bivariate Plots**

The text here describes the bivariate plots in more detail than in the main text, but the main text (section on descriptive anatomy) should be consulted for a discussion of the functional implications of these plots.

Ilium morphology

**Tuber coxa (Figure S1A).** Sthenurines clearly fall above the regression line, showing relatively broader tuber coxae (the dorsal tip of the ilium), especially compared with the species of *Macropus*, which fall on or below the line. The species of *Dendrolagus* (tree-kangaroos) also show broad tuber coxae. The position of the Miocene balbarid *Nambaroo gillespieae* in Figure S1A, as well as that of the New Guinea forest-wallabies (primitive macropodines, *Dorcopsis* and *Dorcopsulus* spp.), suggests that relatively broad tuber coxae are the more generalized macropodid condition.

**Length of ischium (Figure S1B).** Sthenurines mainly fall on or below the regression line, reflecting a relatively short ischium, while the larger species of *Macropus* fall above the line, reflecting a long ischium. Other smaller cursorial macropodids, such as species of *Lagostrophus* (hare-wallabies), *Onychogalea* (nail-tail wallabies) and *Petrogale* (rock-wallabies) also fall above the regression line, while species of *Dendrolagus* fall below it.

**Length of pubo-ischiatic symphysis (Figure S1C).** This figure echos the results in Figure S1B, but shows more clearly the separation between the cursorial macropodines on the one hand (above the line), with the sthenurines and *Dendrolagus* spp. (below the line). The pademelons (*Thylogale* spp., forest-dwelling macropodines that are not specialized hoppers) also fall below the line.

**Length of the epipubic bone (Figure S1D).** Sthenurines fall above the regression line, showing a long epipubis, but in fact most of the macropodines (with the exception of the larger species of *Macropus*) tend to fall above the line. Falling below the line are the potoroines (with the exception of the recently extinct desert rat-kangaroo, *Caloprymnus campestris*) and smaller cursorial macropodines (e.g., the nail-tail wallabies) *Onychogalea* spp.)

Femoral morphology

**Anterior-posterior length of the femoral head (Figure S2A).** Sthenurines, with the exception of the Miocene *Hadronomas puckridgi*, fall above the regression line, showing higher values of femoral head width than large species of *Macropus* (as do *Protemnodon* spp.). The smaller extinct genera cluster with the extant kangaroos. Tree- kangaroos (*Dendrolagus* spp.) also have larger than average femoral heads.

**Distance from femoral head to the base of the lesser trochanter (Figure S2B).** Sthenurines, with the exception of the smaller individuals of *S. occidentalis* (but including the Miocene *Hadronomas puckridgi*), fall above the regression line, with higher values than large species of *Macropus* (as does one species of *Protemnodon*). The smaller extinct genera largely cluster with the extant kangaroos, although *Ngamaroo* *archeri* has rather high values, and *Namburoo gillespieae* has rather low ones. Tree-kangaroos (*Dendrolagus* spp.) also have higher values, as do some of the New Guinea forest-wallabies (*Dorcopsis* and *Dorcopsulus* spp.).

**Distance from proximal femur to the base of the adductor scar (Figure S2C).** Sthenurines, with the exception of the Miocene *Hadronomas puckridgi*, fall above the regression line, with slightly higher values than large species of *Macropus*, showing a more distally-positioned adductor scar. The smaller extinct genera cluster with the extant kangaroos, although *Ngamaroo archeri* appears to have a relatively distally-placed adductor scar, resembling the potoroine *Potorous longipes* (the very low position of the recently extinct desert rat-kangaroo, *Caloprymnus campestris,* possibly relates to it being extremely cursorial).

**Width across the distal femoral condyles (Figure S2D).** The larger Pleistostene sthenurines fall above the regression line, thus having broader knees than large species of *Macropus* (as do the species of *Protemnodon*). The smaller extinct genera cluster with the extant kangaroos. Tree-kangaroos (*Dendrolagus* spp.) also have relatively broad knees.

Tibial morphology

**Length of the tibial tuberosity (Figure S3A).** Medium to large-sized species of *Macropus* (but not the extinct “giant” *M. ferragus*) fall above the regression line, as do other highly cursorial kangaroos such as nail-tail wallabies, *Onychogalea* spp. and hare-wallabies, *Lagorchestes* spp.). Sthenurines follow the normal macropodid pattern. Of the other extinct species, *Protemnodon anak* has relatively high values, and *Ngamaroo archeri* relatively low ones.

Pedal morphology

**Width of the fibular facet on the lateral trochlear ridge of the astragalus (Figure S3B).** Pleistocene sthenurines (i.e., excluding the genera *Hadronomas* and *Rhizosthenurus*) fall above the regression line, with a broader fibular facet than large species of *Macropus*. However, the largest of the extinct “giant” *Macropus*, individuals of *M. ferragus*, also has a relatively broad fibular facet, as does *Protemnodon anak*. The tree-kangaroos (*Dendrolagus* spp.) cluster with the other macropodines, except for the species *D. lumholtzi*, which has a broader than average facet.

**Length of the calcaneal tuber (Figure S3C).** Large species of *Macropus* (including the extinct “giant” species) fall above the regression line, with elongated calcaneal tubers, as does *Protemnodon snewini*. Sthenurines retain the general macropodid condition. Tree-kangaroos (*Dendrolagus* spp.) have relatively short calcaneal tubers.

**Length of the fourth metatarsal (Figure S3D).**  Large species of *Macropus* tend to fall above the regression line, having longer fourth metatarsals than most of the sthenurines and *Protemnodon* spp. Among the other macropodines, more cursorial forms (e.g., nail-tail wallabies, *Onycogalea* spp., and hare- wallabies, *Lagorchestes* spp.) have somewhat longer metatarsals, while tree kangaroos (*Dendrolagus* spp.) have notably shorter ones. The position of the extinct species *Nambaroo gillespieae* and *Ngamaroo archeri* (and the extant musky rat kangaroo, *Hypsiprymnodon moschatus*) suggests that relatively a relatively short fourth metatarsal was the primitive condition for macropodoids. The New Guinea forest-wallabies (*Dorcopsis* and *Dorcopsulus* spp., not labeled) also fall below the regression line.

Long bone scaling relationships

In the plot for femur length versus femur diameter (Figure S4A) the labeled *Simosthenurus occidentalis* is a small individual; other individuals of this species are within the cluster of larger sthenurines (this is true for the other plots in which a small *Si. occidentalis* is identified). The smaller extinct genera cluster with the extant kangaroos. Sthenurines, with the exception of the late Miocene *Hadronomas puckridgi*, have higher values of femoral width than extant macropodines (as do some of the species of *Protemnodon*). The extinct *Macropus titan* has a relatively larger femoral width than most of the individuals of the extant large species of *Macropus.* In the plot of tibia length versus tibia diameter (Figure S4B) the large species of *Macropus* (but not the extinct *M. ferragus*) have relatively gracile tibiae. Sthenurines, with the exception of the Miocene *Hadronomas puckridgi*, have higher values of tibial width than extant macropodines (as does *Protemnodon anak,* but not the smaller *Protemnodon snewini*: this may reflect the relatively short tibia of *P. anak*).

**Figure Legends**

**Figure S1. Ilium length versus other aspects of ilial morphology.** (A) Ilium length versus width of the tuber coxa. (B) Ilium length versus the dorsal length of the ischium. (C) Ilium length versus the ventral length of the puboischiatic symphysis. (D) Ilium length versus the length of the epipubic bone.

**Figure S2. Femur length versus other aspects of femoral morphology. (**A) Femur length versus the anterior-posterior width of the femoral head. (B) Femur length versus the distance of femoral head to the base of the lesser trochanter. (C) Femur length versus the length of the femur from the proximal end to the base of the adductor scar. (D) Femur length versus the width of the femur across the distal condyles.

**Figure S3. Other morphological variables from the tibia and pes:** (A) Tibia average midshaft diameter versus the anterior-posterior length of the tibia tuberosity. (B) Width (medio-lateral) of the base of the astragalus versus the width (dorso-plantar) of the fibular facet on the lateral trochlear ridge of the astragalus. (C) Width of the base of the astragalus versus the length of the calcaneal tuber. (D) Length of the femur versus the length of the fourth metatarsal.

**Figure S4: Scaling of long bone length versus diameter (shown in Figure 11) with labeled taxa. (**A) Femur length versus average femur cross-sectional diameter. (B) Tibia length versus average tibial midshaft cross sectional diameter.
