# Supplementary material for: Locomotion in Extinct Giant Kangaroos: Were Sthenurines Hop-Less Monsters?
Source: PLoS One. 2014 Oct 15;9(10):e109888. doi: 10.1371/journal.pone.0109888 (PMC4198187; doi:10.1371/journal.pone.0109888)
Supplement: Text S2 — Additional discriminant analyses, using all of the taxa. Table A, Coefficient loadings for additional Discriminant Analysis for all of the taxa: all hind limb bones. Table B, Coefficient loadings for additional Discriminant Analysis for all taxa: without the pelvis. (DOC) [file pone.0109888.s013.doc]

**Additional Discriminant Analyses, Using all of the Taxa**

All hind limb bones

Three groups were defined: Three groups were defined: potoroines (plus *Hypsiprymnodon*), other macropodines (including *Lagostrophus* but excluding *Dendrolagus* spp.), and species of *Dendrolagus* (tree-kangaroos). The extinct taxa were entered as unknowns. This analysis yielded two significant functions. For the first function, the value of the Wilks’ lambda statistic was close to zero and highly significant (λ = 0.043; χ2 = 155.255; d.f. = 16 ; P < 0.001). Similarly, the value of the Wilks’ lambda statistic for the second function was also significant (λ = 0.414; χ2 = 43.670; d.f. = 7; P < 0.001). Both functions allowed a 91.2% of correct reclassifications using the leave-one-out method of cross-validation together, which suggests that they combined a set of skeletal traits that accurately distinguishes the three groups compared. The morphospace depicted from the scores of the specimens on both discriminant functions are shown in Figure S5A and the loadings of the variables on each function in Table A.

The first function clearly separates the larger sthenurines, and the tree kangaroos (*Dendrolagus* spp.), with negative scores, from all other macropodoids, with largely positive scores. The small sthenurine *“Procoptodon” gilli*, which was around the mass of a male grey kangaroo, *Macropous giganteus* (~ 60 kg, [2]), clusters with the macropodines. The variables loading with negative values are primarily the width of the astragalus, with some contributions from the length of the second phalanx on digit 4 (this being a sthenurine feature, rather than one typical of *Dendrolagus* spp.), and the length of the iliac blade. The broad astragalus is for different functional reasons in sthenurines and *Dendrolagus* spp.: in sthenurines it relates to the weight-bearing capacity of the foot, while in *Dendrolagus* spp. it relates to the mobility of the ankle joint (a secondary adaptation for climbing). The relatively small size of *“Procopdoton” gilli* may be the reason why it does not cluster with the larger sthenurines.

The variables loading with positive scores are the distal width of the fourth metatarsal, the medio-lateral width of the medial astragalar facet on the tibia, and the dorsal length of the ischium (with smaller contributions from the length of the fourth metatarsal and the length of the epipubic bone. It is challenging to interpret these variables in terms of the functional morphology distinguishing most macropodoids from sthenurines and *Dendrolagus* spp. As noted previously (and see Figure 6B) the distal width of metatarsal IV is considerably broader in *Sthenurus* than in *Macropus*. However, it is possible that *Dendrolagus* spp. have relatively narrow fourth metapodials: as they are either in trees, or when on the ground they usually employ a quadrupedal bound, they would experience less weight-bearing on the hind feet. Thus this feature of the distal metapodial might be distinguishing most macropodoids (but not sthenurines) from *Dendrolagus* spp. The dorsal length of the ischium is an obvious feature, as a longer ischium is seen in more cursorial kangaroos, and both sthenurines and *Dendrolagus* spp. have short ischia (see Figure S1B), but the anatomy of the distal tibia is more perplexing: it may be the case that this is a distinctive potoroine feature (see below), and as potoroines were grouped with other macropodoids (excluding *Dendrolagus* and *Macropus*) for the purposes of this analysis, this might be a potoroine feature that is pulling the other taxa over into the positive area of the morphospace.

The distribution of taxa along the second function is also difficult to interpret. This appears, at least in part, to be distinguishing potoroines (plus *Hypsiprymnodon moschatus*) from other macropodoids (note that the non-potoroine macropodids with negative values tend to be the smaller, more cursorial forms, such as *Lagostrophus*, *Lagorchestes*, and *Onychogalea*). However, this is clearly not simply a size axis, as can be shown by the very scattered distribution of the individuals of the red kangaroo, *Macropus* *rufus*. The taxa with high values tend to be the macropodines with more generalized (less gracile) morphologies, such as the forest-wallabies (*Dorcopsis* and *Dorcopsulus* spp.) and the swamp wallaby (*Wallabia bicolor*), and especially the balbarid *Nambaroo gillespieae*; but note that many large species of *Macropus* also have relatively high positive values (perhaps because their larger size renders them more robust). All of the sthenurines have positive scores on this axis, while the species of *Dendrolagus* are scattered around neutrality.

The highest-loading negative variable on the second component is overwhelmingly the width of the medial astragalar facet on the distal tibia: this appears to the anatomical variable that is distinguishing the potoroines. The length of the iliac blade, the distal width of the fourth metatarsal, and the length of the second phalanx on the fourth digit also have slight negative loadings. The variables loading with high positive values are the mediolateral width of the astragalus, and the dorsal length of the ischium (with smaller contributions from the length of the epipubic bone and the length of the fourth metatarsal). A broad astragalus may reflect larger size (which is why the sthenurines have high positive values) or it may also reflect a relatively generalized foot (which would be why taxa such as *Dorcopsis* and *Nambaroo* have high values). A long ischium is a feature of the larger species of *Macropus* (see Figure S1B), as is a long fourth metatarsal, while *Dendrolagus* spp. have exceptionally short fourth metatarsals (see Figure S3D).

Without the pelvis

The groups were defined as in the previous analysis. The value of the Wilks’ lambda statistic for both of the functions was significant (Function I: λ = 0.053; χ2 = 158.284; d.f. = 14; P < 0.001; Function II: λ = 0.628; χ2 = 25.092; d.f. = 6; P < 0.001), and allowed for the analysis performed here allowed a 84.1% of correct reclassifications using the leave-one-out method of cross-validation. This result indicates that both functions combined a set of skeletal traits that accurately distinguishes the three groups compared. The analysis is visually displayed in Figure S5B, and the loadings of the variables are shown in Table B. The distribution of specimens is somewhat similar to that with the CVA with all the bones. The first factor distinguishes sthenurines (including the Miocene *Hadronomas puckridgi*, but with the exception of *“Procoptodon” gilli*) and *Dendrolagus* spp. from other macropodoids. Note here the inclusion of some species of *Protemnodon*: the relatively gracile *Protemnodon anak* clusters with the extant macropodines, while the more robust *Protemnodon* sp. has the greatest positive values of any taxon. In this plot, with the exception of the large and extremely specialized *Procoptodon* sp., the sthenurines have similar scores on the first factor to *Dendrolagus* spp. (in the analysis with all the bones they had much more negative scores).

Variables with high negative scores are, as with the analysis with the pelvis included, the width of the astragalus, but a more important astragalar variable is the width of the lateral trochlear ridge (= fibular facet), which was previously noted as being especially large in sthenurines (see Figures 5B, S3B). The more distal placement of the adductor scar on the femur is of smaller importance on this factor, a feature that was previously noted as being relatively large in sthenurines and *Dendrolagus* spp. (see Figure S2C). Variables with high positive scores include, as previously, the distal width of the fourth metatarsal, but now also of importance are the length of the lateral tibial condyle on the femur, and the length of the calcaneal tuber. The short length of the calcaneal tuber in sthenurines and, especially, *Dendrolagus* spp. (where it reflects a de-emphasis on the role of the gastrocnemius in locomotion) was previously noted (see Figures 5B, S3C). Also it was noted previously that *Sthenurus* had a longer lateral tibial condyle than *Macropus* (see Figure 3B), but this feature might be one that is acting to separate most macropodoids (with longer condyles) from *Dendrolagus* spp.

The second factor again appears to be one that is distinguishing potoroines (with negative scores) from other macropodids (although note that *Hypsiprymnodon moschatus* and *Potorous longipes* now cluster with the macropodines). The larger species of *Macropus* now cluster together with high positive scores: *Wallabia* and various species of *Dorcopsis* still have fairly high scores, but *Nambaroo* is no longer a positive outlier on the analysis, and in fact has a score of around zero. Within the sthenurines: the specimens of *Sthenurus stirlingi* have more positive scores than *Dendrolagus* spp., but *Simosthenurus occidentalis* and *Procoptodon* sp. have more negative scores.

The variable with the highest negative score is the dorsoplantar width of the calcaneal tuber, which may be a potoroine feature. Also important on this factor with negative values are the width of the lateral trochlear ridge of the astragalus and the length of the lateral tibial condyle. These morphological features do not appear to represent any functional morphological complex. The variable with the highest positive score is the length of the calcaneal tuber, which is reflected in the high positive scores of the larger species of *Macropus*. The placement of the adductor scar is also important here, and this may reflect a macropodine versus a potoroine feature. It is not clear why the different genera of sthenurines are divided on this axis, although *Procoptodon* and *Simosthenurus* also cluster apart from the other sthenurines on the PCA of the bones without the pelvis.

Summary

The analyses used three groupings: potoroines (plus *Hypsiprymnodon*); macropodines (plus *Lagostrophus*) with the exception of *Dendrolagus* spp.; *Dendrolagus* spp.; with the extinct taxa added as unknowns. Although these analyses clearly distinguished the larger sthenurines from other macropodoids (*“Procoptodon” gilli* clustered with the macropodines), the results were more difficult to interpret than those of the PCA, especially as the selected variables did not always make sense in terms of a functional morphologically adaptive complex. The first function always separated the larger sthenurines plus *Dendrolagus* spp. from other macropodoids, with the sthenurines having the most negative scores. In the analysis without the pelvis, the Miocene sthenurine *Hadronomas puckridgi* (which was not included in the first analysis) clustered with the larger sthenurines (in contrast to the PCA where it fell close to the similarly-sized *“Procoptodon” gilli*.) In the analysis with all hind limb bones, the second function separated the sthenurines from *Dendrolagus* spp. In the analysis without the pelvis the sthenurines divided into two groups, one with more positive scores than *Dendrolagus* spp., and one with more negative scores (the sthenurines with the negative scores were *Procoptodon* sp. and *Simosthenurus occidentalis*, which were noted to cluster together in other analyses). The significance of the distribution of the other taxa along second factor was difficult to interpret: this factor seems in part to be one distinguishing potoroines from other extant macropodoids.

**Figure Legends**

**Figure S5. Additional Discriminant Analyses.** (A) Using all of the hind limb bones, for all of the taxa. (B) Without the pelvis, for all of the taxa.

**Table A. Coefficient loadings for additional Discriminant Analysis for all of the taxa: all hind limb bones.**

| Loading on Factor 1 | | | Loading on Factor 2 | | |
| --- | --- | --- | --- | --- | --- |
| Var. | Variable Description | Load. | Var. | Variable Description | Load. |
| M5 | Distal lateral width of fourth metatarsal | 0.571 | A2 | ML width of astragalus | 0.294 |
| T13 | ML width of medial astragalar facet | 0.354 | P7 | Dorsal length of ischium | 0.237 |
| P7 | Dorsal length of ischium | 0.253 | P15 | Length of epipubic bone | 0.109 |
| M1 | Length of fourth metatarsal | 0.099 | M1 | Length of fourth metatarsal | 0.012 |
| P15 | Length of epipubic bone | 0.021 | Ph2L | Length of second phalanx on fourth digit | -0.066 |
| P10 | Length of iliac blade | -0.193 | M5 | Distal lateral width of fourth metatarsal | -0.135 |
| Ph2L | Length of second phalanx on fourth digit | -0.247 | P10 | Length of iliac blade | -0.180 |
| A2 | ML width of astragalus | -0.748 | T13 | ML width of medial astragalar facet | -0.789 |

Key: ML = mediolateral.

**Table B. Coefficient loadings for additional Discriminant Analysis for all taxa: without the pelvis.**

| Loading on Factor 1 | | | Loading on Factor 2 | | |
| --- | --- | --- | --- | --- | --- |
| Var. | Variable Description | Load. | Var. | Variable Description | Load. |
| M5 | Distal lateral width of metatarsal IV | 0.486 | C3 | Medial length of calcaneal tuber | 0.062 |
| F19 | Length of lateral tibial condyle | 0.358 | F19 | Length of lateral tibial condyle | 0.061 |
| C3 | Medial length of calcaneal tuber | 0.294 | A2 | ML width of astragalus | 0.013 |
| C4 | DP width of midshaft of calcaneal tuber | 0.040 | M5 | Distal lateral width of metatarsal IV | -0.101 |
| F15 | Length of femur to adductor scar | -0.092 | A4 | AP width of lateral trochlear ridge | -0.161 |
| A2 | ML width of astragalus | -0.984 | F19 | Length of lateral tibial condyle | -0.162 |
| A4 | AP width of lateral trochlear ridge | -1.368 | C4 | DP width of midshaft of calcaneal tuber | -0.589 |

Key: AP = anteroposterior; DP = dorsoplantar; ML = mediolateral.
